# A framework for spontaneous Brillouin noise: unveiling fundamental limits in Brillouin metrology

**DOI:** 10.1038/s41377-025-02115-2

**Published:** 2026-01-03

**Authors:** Simeng Jin, Shuai Yao, Zhisheng Yang, Zixuan Du, Xiaobin Hong, Marcelo A. Soto, Jingjing Xie, Long Zhang, Fan Yang, Jian Wu

**Affiliations:** 1 State Key Laboratory of Information Photonics & Optical Communications, Beijing University of Posts and Telecommunications, Beijing, China; 2 Shanghai Institute of Optics and Fine Mechanics, Chinese Academy of Sciences, Shanghai, China; 3 Department of Electronics Engineering, Universidad Técnica Federico Santa María, Valparaíso, Chile; 4 School of Physical Science and Technology & State Key Laboratory of Advanced Medical Materials and Devices, ShanghaiTech University, Shanghai, China

**Keywords:** Microscopy, Nonlinear optics, Imaging and sensing

## Abstract

Spontaneous Brillouin scattering (SpBS) enables non-contact probing of mechanical and thermodynamic material properties, underpinning transformative technologies such as distributed optical fiber sensing and high-resolution microscopy. Achieving ultimate precision in these systems demands a fundamental understanding of noise limits. Yet, an intrinsic SpBS noise phenomenon proposed over three decades ago has remained largely unexplored, particularly in metrological contexts. Here, we revisit the physical mechanism and stochastic nature of this long-overlooked noise source, developing a comprehensive analytical framework, validated through dedicated experiments. Crucially, we propose, for the first time, that SpBS noise constitutes a universal and fundamental limit capable of surpassing conventional constraints (e.g., the shot-noise limit) in spontaneous Brillouin metrological systems, such as imaging, microscopy and sensing. We experimentally demonstrate the SpBS-noise-limited regime in Brillouin imaging and sensing scenarios. This framework establishes a critical foundation for understanding and optimizing the performance of current and future Brillouin-based technologies across a broad range of applications.

## Introduction

Spontaneous Brillouin scattering (SpBS) is a fundamental inelastic light-matter interaction present in virtually all media^1^. First theorized by Léon Brillouin in 1922, this process arises from the interaction between incident photons and thermally driven density fluctuations—quantized as acoustic phonons—resulting in frequency-shifted scattered photons at Stokes or anti-Stokes wavelengths^2^. The peak frequency shift, linewidth, and intensity of the Brillouin spectrum are inherently linked to material properties, such as elastic and thermal characteristics. This fundamental relationship underpins the versatility of SpBS, enabling its application in a wide range of technologies: from non-intrusive long-distance distributed strain and temperature sensing in optical fibers^3–14^, to photon-phonon interactions in integrated waveguides^15–17^, and non-contact, three-dimensional mechanical imaging with high spatial resolution in biological and medical samples via Brillouin microscopy^18–29^.

The performance of these techniques depends critically on precise spectral measurements, which in turn demand maximizing the system signal-to-noise ratio (SNR). Conventional approaches to SNR enhancement focus on minimizing the impact of detection noise, primarily thermal and shot noise, with the shot-noise limit broadly regarded as the fundamental boundary, where SNR scales with the square root of signal power. However, the stochastic nature of SpBS can introduce additional noise, reducing the SNR of these systems. This issue was first examined in the context of Brillouin optical time-domain analysis (BOTDA)^30,31^, where SpBS acts as an unwanted disturbance. In such a case, SpBS is typically modeled as having constant intensity, with randomness attributed only to its phase and polarization fluctuations^30,31^. More recently, the impact of SpBS has also been studied in Brillouin optical time-domain reflectometry (BOTDR)^13^, a distributed sensing technique in which SpBS itself serves as the signal of interest. Even in this case, SpBS continues to be modeled as a constant-power signal with random phase and polarization variations, and under this assumption, polarization noise has been identified as the main factor limiting sensing performance^13^. So far, no other noise source is considered to be universally present in spontaneous Brillouin measurement systems.

However, in 1990, a seminal theoretical study^32^ proposed an intrinsic noise source: intensity fluctuations in the Brillouin scattered light caused by the stochastic nature of thermally excited phonons. Besides explaining the physical origin of this noise, this work^32^ has also investigated its stochastic features via numerical simulations. In the strongly stimulated regime (with $G\approx 23$ and $70$, where $G={gIL}$ is the single-pass Brillouin gain, for which $g$ is the SBS gain factor, $I$ is the intensity of the incident laser field, and $L$ is the length of the Brillouin medium), these predictions were experimentally verified shortly thereafter^33^. In this high-gain regime, the saturated pump nonlinearly amplifies the SpBS signal and distorts its stochastic evaluation. Yet, in the spontaneous regime ($G\ll 21$)^34^, these fluctuations have remained largely unexplored for over three decades, neither experimentally observed nor accounted for in practical metrological applications. The absence of an analytical treatment and lack of connection to real-world system parameters have limited both theoretical understanding and practical relevance. Critically, the implications of this intrinsic noise for measurement precision have gone unexamined since Brillouin scattering was first proposed more than a century ago^2^, leaving a significant gap in spontaneous Brillouin metrology.

Here, we address this longstanding gap by developing an analytical framework that comprehensively characterizes the intrinsic noise of SpBS in the spontaneous regime. Building on a systematic analysis by revisiting its physical formation mechanism and stochastic properties, we show that this noise imposes a universal, previously unrecognized upper bound on the SNR, one that can exceed the conventional shot-noise limit. We validate our predictions experimentally using both coherent and direct detection schemes with Brillouin gain coefficients $G\approx 0.23$ and $G\approx 3.89$, respectively. These measurements represent the first observation of intrinsic SpBS noise in the spontaneous regime. Finally, we demonstrate the metrological relevance of this noise by re-evaluating the performance of several SpBS-based platforms, including Brillouin imaging, microscopy, and distributed fiber sensing. Our framework reveals that SpBS noise can dominate in many practical systems, offering a fundamental basis for redesigning and optimizing spontaneous Brillouin metrological technologies under intrinsic noise constraints.

## Results

### Revisiting the physical formation mechanism of SpBS noise

**Fig. 1:**
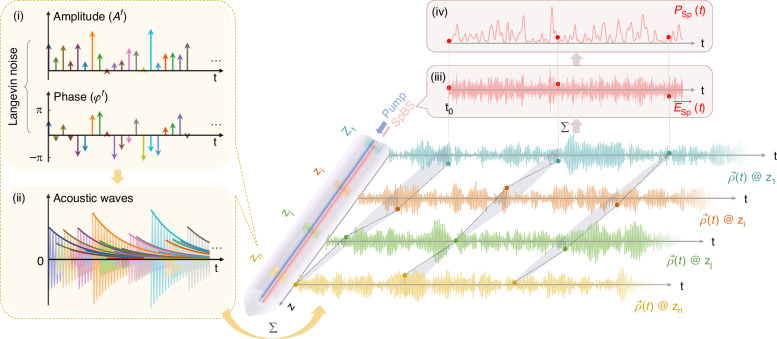
**Schematic description on the physical formation mechanism of spontaneous Brillouin scattering intensity fluctuations**. ${A}^{f}$: Amplitude of Langevin noise. ${\varphi }^{f}\in$ [-π, π]: Phase of Langevin noise. $\mathop{\to }\limits_{P}\left(t\right)$: Complex amplitude of acoustic wave. $\overrightarrow{{E}_{\mathrm{Sp}}}\left(t\right)$: Complex amplitude of SpBS field. ${P}_{{\rm{Sp}}}\left(t\right)$: Time-domain power envelope of SpBS. The light purple irregular cylinder marks the region where light interacts with matter, while the blue and red beams represent the incident pump light and the backward-propagating SpBS light, respectively. Four specific positions (${z}_{1}$, ${z}_{i}$, ${z}_{j}$, and ${z}_{n}$) within the interaction region are highlighted in different colors as examples. (i)**-**(ii) exemplify the local excitation of acoustic waves by Langevin noise at ${z}_{n}$. (i) illustrates ${A}^{f}$ and the corresponding ${\varphi }^{f}$ of Langevin noise in the time domain. (ii) shows the acoustic waves driven by each Langevin noise element, where different light-colored curves denote individual acoustic waves, and the corresponding dark-colored curves depict the corresponding acoustic wave envelopes. $\vec{\rho }\left(t\right)$ at the four example positions are color-matched to their respective locations. (iii) shows $\overrightarrow{{E}_{\mathrm{Sp}}}\left(t\right)$ results from the cumulative effect of these acoustic waves observed at the same moment. (iv) shows ${P}_{{\rm{Sp}}}\left(t\right)$ extracted from $\overrightarrow{{E}_{\mathrm{Sp}}}\left(t\right)$ in (iii)

As the first component of our framework, we revisit the physical formation mechanism of spontaneous Brillouin scattering (SpBS) noise by deriving a stepwise solution to the classical three-wave coupling equations (Supplementary Note S1 Eqs. (S1)–(S3))^1,32,35^. This approach provides a physically intuitive picture of how SpBS noise arises from fundamental thermodynamic interactions, as schematically illustrated in Fig. 1.

To facilitate both intuitive visualization and later mathematical characterization of the stochastic SpBS intensity fluctuations, for which the discrete-domain signal is required, we discretize the light-matter interaction length into small segments of length $\varDelta z$ (Fig. 1). In each segment, thermally induced material vibrations are treated as localized Langevin forces, represented by a series of temporally spaced delta functions with random amplitudes ${A}^{f}$ and phases ${\varphi }^{f}$ separated by time intervals $\varDelta t$ (Fig. 1(i); Supplementary Note S1 Eq. (S1)). These stochastic forces drive localized acoustic fields $\vec{\rho }(t)$, which can be expressed as a superposition of exponentially decaying sinusoidal waves at a common carrier frequency, with random amplitudes and phases dictated by the Langevin excitation (Fig. 1(ii); Supplementary Note S1 Eq. (S9)).

The resulting SpBS field $\overrightarrow{{E}_{\mathrm{Sp}}}\left(t\right)$ is formed by the cumulative interaction of these stochastic acoustic waves with the pump field $\vec{{E}_{P}}$ over the entire interaction length (Fig. 1; Supplementary Note S1 Eq. (S10)). Assuming a constant pump, $\overrightarrow{{E}_{\mathrm{Sp}}}\left(t\right)$ becomes proportional to the spatial summation of time-delayed acoustic wave contributions (Fig. 1(iii); Supplementary Note S1 Eq. (S12)). This results in the SpBS power envelope ${P}_{{\rm{Sp}}}(t)$, which inherently exhibits stochastic intensity fluctuations (Fig. 1(iv)). Assuming ideal condition (i.e., sufficiently large measurement bandwidth and appropriate sampling, as detailed in the next Section), we analytically derive the signal-to-noise ratio (SNR) of ${P}_{{\rm{Sp}}}(t)$, defined as the ratio of its mean to standard deviation (STD). This analysis yields an SNR of unity (detailed in Supplementary Note S2), consistent with the earlier prediction^32^.

The formulation process presented in Fig. 1 offers an intuitive link between microscopic stochasticity and macroscopic noise on ${P}_{{\rm{Sp}}}(t)$, hereafter referred to as SpBS noise.

### Analyzing the stochastic properties of SpBS noise

As the second component of our framework, we extend the analysis to systematically characterize the stochastic properties of SpBS intensity fluctuations and quantify their dependence on system-level parameters. We show that the unity SNR does not generally hold in practice. Instead, the SNR can vary significantly depending on system bandwidth (${B}_{{\rm{m}}}$) and sampling parameters—critical yet previously unaddressed factors that are intrinsic to practical spontaneous Brillouin metrology systems.

**Fig. 2:**
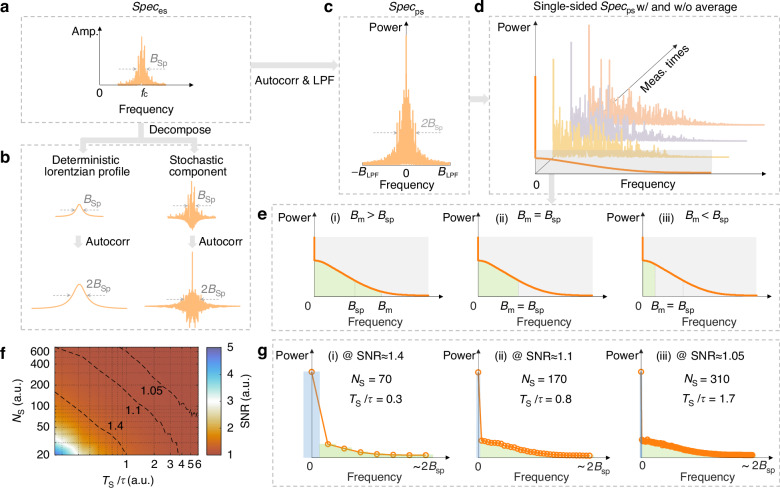
**Analysis on the stochastic behaviors of SpBS intensity fluctuations**. ${B}_{{\rm{Sp}}}$: Full width at half maximum (FWHM) of the SpBS field spectrum. ${B}_{{\rm{m}}}$: System measurement bandwidth. ${T}_{{\rm{S}}}$: Sampling interval. ${N}_{{\rm{S}}}$: Total number of samples. $\tau$: acoustic lifetime. **a** Spectrum of the scattered field $\overrightarrow{{E}_{\mathrm{Sp}}}\left(t\right)$, denoted as ${Spe}{c}_{{\rm{es}}}$, centered at ${f}_{{\rm{c}}}$. The property of ${Spe}{c}_{{\rm{es}}}$ is consistent across detection methods, differing only in the central frequency ${f}_{{\rm{c}}}$ (optical or electrical frequency) depending on whether direct or coherent detection is used. It can be decomposed into a deterministic Lorentzian profile and a stochastic component, illustrated in (**b**), along with their respective autocorrelation results. **c** Double-sided spectrum (${Spe}{c}_{{\rm{ps}}}$) of ${P}_{{\rm{Sp}}}\left(t\right)$, obtained via the autocorrelation of ${Spe}{c}_{{\rm{es}}}$, followed by a low-pass filtering (LPF) process. **d** Single-sided form of ${Spe}{c}_{{\rm{ps}}}$, in the cases with and without averaging. Light yellow, light purple, and light pink curves correspond to three unaveraged power envelope spectra, while dark orange curve denotes the power envelope spectrum after averaging. The averaged spectral segment within the gray box is used for further analysis in (**e**). **e** Effect of different ${B}_{{\rm{m}}}$ on the SpBS power envelope spectrum with a FWHM of ${B}_{{\rm{Sp}}}$. Insets (i), (ii), and (iii) depict the cases of ${B}_{{\rm{m}}} > {B}_{{\rm{Sp}}}$, ${B}_{{\rm{m}}}={B}_{{\rm{Sp}}}$, and ${B}_{{\rm{m}}} < {B}_{{\rm{Sp}}}$, respectively. Green shading indicates the captured part of the AC component, representing the preserved SpBS noise after filtering. **f** Theoretical 2D SNR map as a function of ${T}_{{\rm{S}}}$ and ${N}_{{\rm{S}}}$, with $\tau$ = 6 ns. Dashed contour lines (black dashed lines) on the map indicate SNR levels of 1.4, 1.1, and 1.05. **g** Power envelope spectra of SpBS under different sampling conditions. Inset (i)–(iii) showcase the DC (blue shading) and AC (green shading) contributions within spectral range [0, $2{B}_{{\rm{Sp}}}$], under three sampling conditions associated with the contour lines in (**f**), representing SNR values of ~1.4, ~1.1, and ~1.05, respectively

To elaborate these effects, we here analyze the spectral properties of SpBS fluctuations. The spectrum of the scattered field $\overrightarrow{{E}_{\mathrm{Sp}}}\left(t\right)$ denoted as ${Spe}{c}_{{\rm{es}}}$ (Fig. 2a), with 3 dB bandwidth ${B}_{{\rm{Sp}}}$ (ref.^1,32^.) that approximately equals to the effective bandwidth, is considered to comprise two components (Fig. 2b): 1) a deterministic Lorentzian profile, representing the conventional Brillouin response, and 2) a stochastic contribution arising from intrinsic SpBS noise, the focus of this study.

The power spectrum of envelope signal ${P}_{{\rm{Sp}}}(t)$, denoted as ${Spe}{c}_{{\rm{ps}}}$ (Fig. 2c), is obtained via autocorrelation of ${Spe}{c}_{{\rm{es}}}$, followed by low-pass filtering (LPF). Its single-sided form (Fig. 2d) contains:

1. a realization-dependent alternating-current (AC) component with 3 dB bandwidth of ${B}_{{\rm{Sp}}}$, corresponding to the time-domain intensity fluctuations of ${P}_{{\rm{Sp}}}(t)$. According to Wiener-Khinchin theorem^36^, the total power of this AC component corresponds to the noise variance of ${P}_{{\rm{Sp}}}(t)$.
2. a constant direct-current (DC) component, originating from the stochastic part of ${Spe}{c}_{{\rm{es}}}$ (Fig. 2b), representing the squared mean (signal power) of ${P}_{{\rm{Sp}}}(t)$ (ref.^36^).

In the ideal full-bandwidth case, the DC and AC powers of ${Spe}{c}_{{\rm{ps}}}$ are equal, yielding ${SNR}=1$. However, in practical systems with finite measurement bandwidth ${B}_{{\rm{m}}}$, only a portion of the AC term may be captured while the DC term remains unaffected (Fig. 2e), leading to a bandwidth-dependent SNR as (Supplementary Note S2):

1 $${SN}{R}_{{\rm{Sp}}}=\sqrt{\frac{{\rm{\pi }}}{2\arctan \left(\frac{{B}_{{\rm{m}}}}{{B}_{{\rm{Sp}}}}\right)}}$$

As ${B}_{{\rm{m}}}$ increases beyond ${B}_{{\rm{Sp}}}$, the SNR approaches and eventually saturates at the value of 1. This relation implies that Brillouin systems operating in different bandwidth regimes will exhibit distinct, yet predictable, SNR behaviors when constrained by SpBS noise.

Furthermore, the sampling interval (${T}_{{\rm{S}}}$) and total number of samples (${N}_{{\rm{S}}}$) also strongly impact on the observed SNR. Figure 2f presents a theoretical 2D SNR map (see “Materials and Methods”) as a function of these sampling parameters, with contour lines illustrating how oversampling can artificially inflate the SNR by distorting the relative DC/AC power ratio. Representative spectral decompositions under three sampling conditions (Fig. 2g) illustrate these effects, with more detailed interpretation in Supplementary Fig. S1.

These findings clarify that ${SNR}=1$ is a limiting case, achievable only when both ${B}_{{\rm{m}}} > {B}_{{\rm{Sp}}}$ and sufficiently low sampling are considered. This analysis provides both practical design guidance for capturing intrinsic SpBS noise and a quantitative foundation for predicting and optimizing SNR in diverse spontaneous Brillouin-based metrological platforms.

### Experimental validation of the framework via two experimental setups

Despite its theoretical prediction over three decades ago^32^, the intensity fluctuations in spontaneous Brillouin scattering (SpBS) have yet to be experimentally verified. Here, we present the first experimental analysis and quantitative characterization of SpBS noise. Our framework is validated under controlled conditions using two dedicatedly designed, fundamental detection schemes: coherent detection and direct detection.

#### Coherent detection setup

**Fig. 3:**
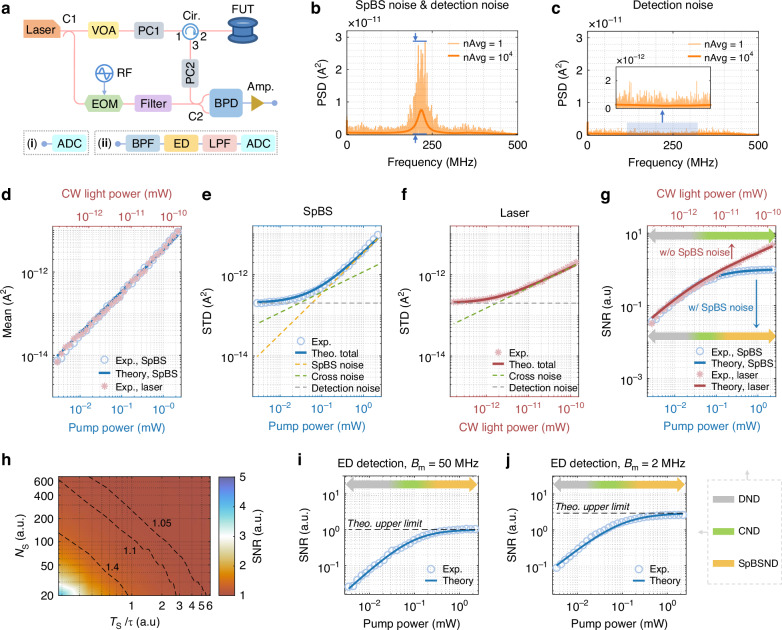
**Experimental validation of the proposed framework via optical fiber-based coherent detection**. **a** Experimental setup for (i) fast Fourier transform (FFT)-based and (ii) envelope detection (ED)-based coherent detection using a 400 m polarization maintaining fiber. **b** Power spectral density (PSD) of the beating signal at CW pump power of 1.8 mW with and without averaging (10,000 averages vs. none). Blue arrows highlight peak-to-peak noise fluctuation at the Brillouin spectrum center. **c** PSD of the noise floor without pump light; inset shows zoomed-in view around the Brillouin frequency. **d** Mean signal vs. pump power ( − 25.5 to 3.5 dBm, 1 dB steps), with experimental results (light blue) and theoretical predictions (dark blue). Pink asterisks indicate CW laser measurements at the same power as SpBS light. **e** Noise standard deviations (STDs) with SpBS signal: experimental (light blue) and theoretical (dark blue), including decomposed contributions from SpBS (yellow), cross-term (green), and detection noise (grey). **f** Noise STDs when SpBS light is replaced by CW laser: experimental (pink) and theoretical (red). **g** SNRs SNR vs. pump power: measured (light blue and pink) and predicted (dark blue and red) values with SpBS and CW light, respectively. Color-gradient arrow (grey → green → yellow) represents shifting dominance from detection noise (DND) to cross-term (CND) to SpBS noise (SpBSND). **h** Experimentally obtained 2D SNR map as a function of sampling interval and total number of sampling points. Contours correspond to SNR values of 1.4, 1.1, and 1.05. **i, j** SNR vs. pump power for system bandwidths of 50 MHz and 2 MHz, respectively, using ED-based measurements and theoretical models. Dashed black lines indicate SpBS-limited SNR ceilings. Color-gradient arrows reflect evolving noise contributions, consistent with (**g**)

Coherent detection is widely adopted for its optimal balance between simplicity and SNR. We implement a fiber-based coherent detection scheme (Fig. 3a), incorporating a 400 m polarization-maintaining fiber (PMF) to mitigate polarization fading (“Materials and Methods”). A continuous-wave (CW) laser with 1.8 mW pump power is launched into the fiber, and the output spectrum is obtained via fast Fourier transform (FFT) analysis (Fig. 3b). The non-averaged spectra reveal pronounced amplitude fluctuations near the Brillouin peak ( ~ 220 MHz), which decay with the spectral envelope and vanish when the pump is turned off (Fig. 3c), strongly implicating SpBS noise as their origin.

We developed an SNR model for the Brillouin peak power ${r}_{{\rm{Co}}}^{{\rm{FFT}}}$, by leveraging our framework to incorporate all relevant noise sources, as (see Supplementary Note S3 for derivation):

2 $$\mathrm{SNR}\left\{{r}_{\mathrm{Co}}^{\mathrm{FFT}}\right\}=\frac{{{{\mathcal{R}}}_{{\rm{p}}}}^{2}{P}_{\mathrm{Lo}}\bar{{P}_{\mathrm{Sp}}}}{\sqrt{\frac{1}{{{SN}{R}_{\mathrm{Sp}}}^{2}}{{{\mathcal{R}}}_{{\rm{p}}}}^{4}{{P}_{\mathrm{Lo}}}^{2}{\bar{{P}_{\mathrm{Sp}}}}^{2}+\frac{{\rm{\pi }}{B}_{\mathrm{Sp}}}{2}{{{\mathcal{R}}}_{{\rm{p}}}}^{2}{P}_{\mathrm{Lo}}\bar{{P}_{\mathrm{Sp}}}{{\sigma }_{e}}^{2}+{\left(\frac{{\rm{\pi }}{B}_{\mathrm{Sp}}}{4}\right)}^{2}{{\sigma }_{e}}^{4}}}\le {SN}{R}_{\mathrm{Sp}}$$

where ${{\mathcal{R}}}_{{\rm{p}}}$ is the photodiode responsivity; ${P}_{{\rm{Lo}}}$ is the local oscillator (OLO) power; $\bar{{P}_{{\rm{Sp}}}}$ is the mean SpBS optical power of the Stokes component entering the photodiode; ${{\sigma }_{e}}^{2}$ is and power spectral density (PSD) of the detection noise; ${B}_{{\rm{Sp}}}$ is the Brillouin linewidth. Note that by default $\bar{{P}_{{\rm{Sp}}}}$ used hereafter corresponds to the Stokes power, which shares same stochastic properties of the anti-Stokes component, as described in detail in Supplementary Note S3 and Fig. S5. Compared to Eq. (1), the SNR in this case is lower than or equal to ${SN}{R}_{{\rm{Sp}}}$ (i.e., the SNR of SpBS intensity fluctuations itself), due to the contribution of three types of noise:

1) *Signal-dependent noise (SpBS noise)*: This corresponds to the first term under the square root in Eq. (2), which shows that the noise standard deviation scales linearly with $\bar{{P}_{{\rm{Sp}}}}$ and dominates at high powers.

2) *Cross-term noise*: This noise is given by the second term under the square root in Eq. (2), indicating that it arises from interaction between signal and detection noise, scaling with $\sqrt{\bar{{P}_{{\rm{Sp}}}}}$.

3) *Detection noise*: This is described by the third term under the square root in Eq. (2), and includes shot and thermal noise, largely independent of $\bar{{P}_{{\rm{Sp}}}}$ due to the normal use of a strong OLO.

At high signal powers, the first term dominates, and the SNR asymptotically approaches the theoretical SpBS noise limit ${SN}{R}_{{\rm{Sp}}}=1$, consistent with FFT-based measurements where ${B}_{{\rm{m}}}\ge {B}_{{\rm{Sp}}}$ always holds (Supplementary Note S3).

Experimental measurements of the Brillouin signal amplitude, noise STD, and SNR over a range of pump powers (-25.5 dBm to 3.5 dBm, blue circles in Fig. 3d-g) show excellent agreement with theoretical predictions, validating the saturation behavior predicted by Eq. (2). As the highest pump power here corresponds to a gain coefficient $G\approx 0.23$, which remains well below the stimulated scattering threshold $G=21$, these results constitute the first experimental confirmation of SpBS fluctuations.

To further isolate the SpBS noise, we have replaced the scattered signal with a CW laser of equivalent power (pink asterisks in Fig. 3d-g)). This eliminates the SpBS signal-dependent noise term while preserving other noise components. The resulting SNR (pink asterisks in Fig. 3g) exceeds unity and deviates from the SpBS-limited case (blue), confirming that SpBS noise indeed defines the saturation ceiling. For a clearer understanding of each noise contribution as a function of the pump power, see Supplementary Fig. S6a, where this is quantified in detail.

The importance of sampling conditions is further evidenced in a 2D experimental SNR map (Fig. 3h). Compared to the idealized model (Fig. 2f), a slight leftward shift is observed, attributable to additional detection noise. This highlights the importance of system optimization, as emphasized in our framework.

Finally, replacing the FFT analysis with an electrical envelope detector (Fig. 3a(ii)) allows direct time-domain observation of SpBS power fluctuations. This approach sets the measurement bandwidth ${B}_{{\rm{m}}}$ by the detector bandwidth. For two representative bandwidths (50 MHz and 2 MHz), the observed SNR (Fig. 3i, j) matches the theoretical curves obtained from Eq. (1), verifying the bandwidth-dependent SNR behavior predicted uniquely by our framework.

#### Direct detection setup

**Fig. 4:**
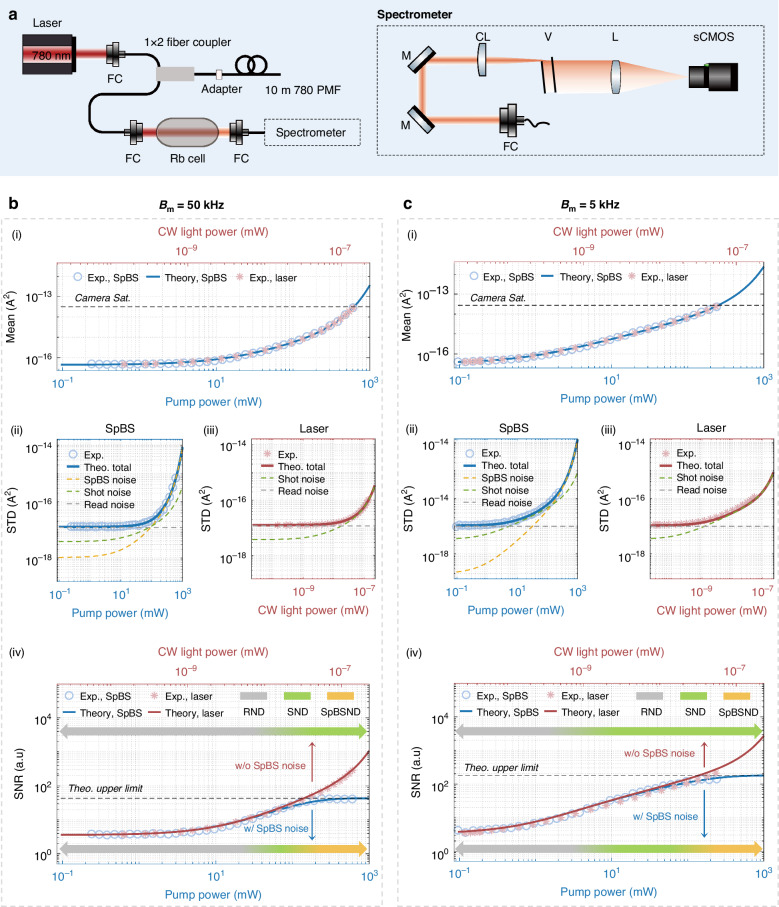
**Experimental validation of the proposed framework via direct detection**. **a** Experimental setup. Rb cell Rubidium cell, FC fiber coupler/collimator, CL cylindrical lens, M mirror, V Virtually Imaged Phased Array (VIPA), L lens. **b** Experimental (blue circles: using SpBS light, pink asterisks: using CW laser light) and theoretical (blue solid lines: using SpBS light, red solid lines: using CW laser light) results with system bandwidth ${B}_{{\rm{m}}}=$ 50 kHz, presented in four subplots: (i) signal mean, (ii) noise STD with SpBS signal, (iii) noise STD with CW laser light replacing SpBS signal, and (iv) SNR. The pump powers range from -6.2 dBm to 27.8 dBm in 1 dB step. The maximum pump power is limited by the camera saturation (black dashed line in (i)). The theoretical total noise STD is obtained by taking the square root on the square sum of all theoretical noise STDs (yellow dashed line: SpBS noise STD; green dashed line: shot noise STD; grey dashed line: thermal noise STD). In (iv), the SNR upper limit constrained by SpBS noise is denoted as the black dashed line. Color-gradient arrow bars, transitioning from grey to yellow, illustrate the evolving domination of thermal noise, shot noise, and SpBS noise with increasing pump power. RND read noise domination, SND shot noise domination, SpBSND SpBS noise domination. **c** Experimental and theoretical results with system bandwidth ${B}_{{\rm{m}}}=$ 5 kHz, following the same illustration approach as in **b**. The pump powers used in experiments (in the presence of the SpBS signal) range from -10.2 dBm to 23.8 dBm in 1 dB increments

To robustly validate the stochastic nature of SpBS noise, we turn to direct detection measurement on a 10 m long fiber sample using a virtually imaged phased array (VIPA)-spectrometer and a camera (Fig. 4a; “Materials and Methods”). Since the Brillouin frequency shift of the fiber ( ~ 22 GHz at 780 nm pump) exceeds one free spectral range (FSR) of the VIPA ( ~ 15.3 GHz), we employ a reflective grating to provide orthogonal dispersion, enabling full spectral separation of the Brillouin signal in combination with VIPA dispersion (Supplementary Fig. S8). Compared to coherent detection, this direct detection scheme offers linear superposition of noise sources and clearer interpretability, enabling a direct expression of the SNR of the camera response ${r}_{{\rm{Di}}}$, which includes contributions from SpBS noise, shot noise, and scientific complementary metal-oxide-semiconductor (sCMOS) camera read noise (background noise):

3 $$\mathrm{SNR}\left\{{r}_{\mathrm{Di}}\right\}=\frac{{{\mathcal{R}}}_{{\rm{c}}}\bar{{P}_{\mathrm{Sp}}}}{\sqrt{\frac{1}{{{SN}{R}_{\mathrm{Sp}}}^{2}}{{{\mathcal{R}}}_{{\rm{c}}}}^{2}{\bar{{P}_{\mathrm{Sp}}}}^{2}+2q{{\mathcal{R}}}_{{\rm{c}}}\bar{{P}_{\mathrm{Sp}}}{B}_{{\rm{m}}}+{{\sigma }_{\mathrm{re}}}^{2}}}\le {SN}{R}_{\mathrm{Sp}}$$

where ${{\mathcal{R}}}_{{\rm{c}}}$ is camera responsivity; $q=1.6\times {10}^{-19}$ is the elementary charge. ${{\sigma }_{{\rm{re}}}}^{2}$ represents the constant variance of the camera read noise, which, unlike thermal noise, remains largely insensitive to bandwidth variations under the low-noise mode of typical sCMOS used in this study^37,38^. The model predicts square-root scaling of SNR at low powers (shot-noise-limited), and saturation at high powers (SpBS-noise-limited) governed by Eq. (1).

Measurements for bandwidths ${B}_{{\rm{m}}}$ = 50 kHz (maximum bandwidth of the sCMOS camera used) and 5 kHz (Fig. 4b, c), both within the ${B}_{{\rm{m}}} < {B}_{{\rm{Sp}}}$ regime, show strong agreement with Eq. (3). For these two bandwidths, the detailed contributions of each individual noise as a function of the pump power are quantified and shown in Supplementary Fig. S6b, c. The maximum gain coefficient $G\approx 3.89$ remains well within the spontaneous scattering regime. Replacing the SpBS light with CW light again yields higher SNR values (pink asterisks), consistent with the model excluding SpBS noise (red solid lines). These results confirm that the observed SNR saturation in direct detection is governed by SpBS noise, in full agreement with our framework.

### Implementation of our Brillouin noise framework on spontaneous Brillouin metrological applications

To underscore the technological relevance of the presented framework, we apply it to evaluate the performance limits of several spontaneous Brillouin-based metrological platforms, incorporating both SpBS noise and conventional noise sources. We here focus on three applications: Brillouin imaging, Brillouin microscopy, and distributed optical fiber sensing.

#### Brillouin imaging and microscopy

**Fig. 5:**
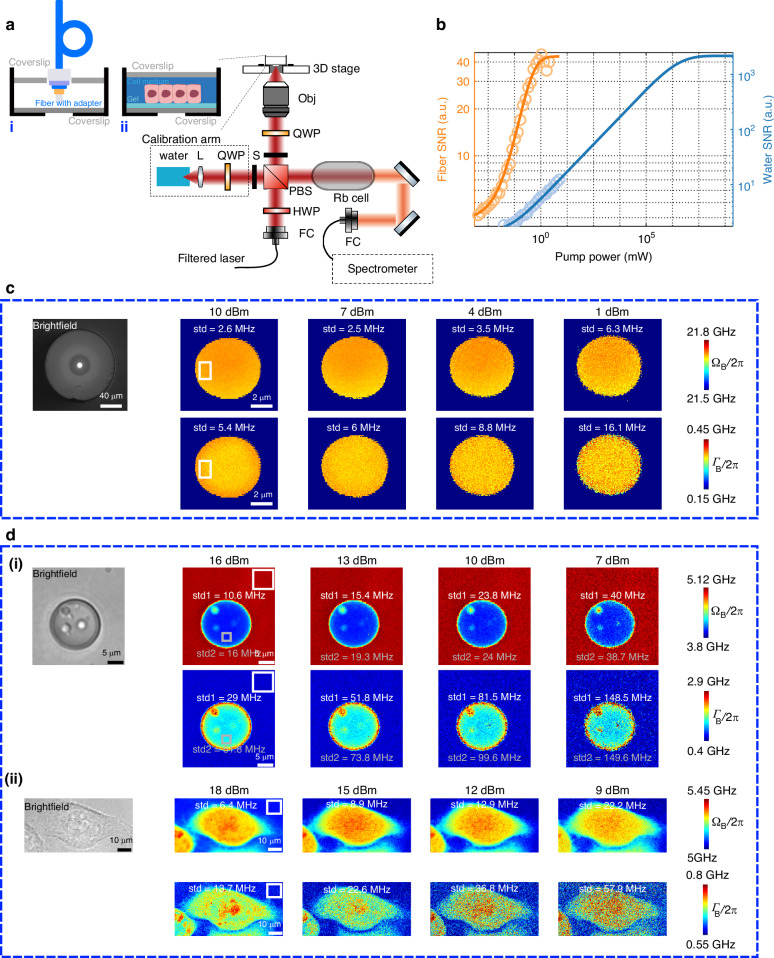
**Implementation of the proposed framework to evaluate the performance of Brillouin imaging and microscopy**. **a** Experimental setup. Obj: objective; QWP: quarter-wave plate; S: optical shutter; PBS: polarizing beam splitter; HWP: half-wave plate. **b** SNR vs. pump power for two samples: a fiber (Brillouin imaging) and double-distilled water (Brillouin microscopy). For the fiber, pump power ranges from −20.8 to 13.2 dBm with 50 kHz system bandwidth; for water, from -4 to 17 dBm with 50 Hz bandwidth. Water sample preparation follows the method described in panel **a**, sample (ii). **c** Brillouin imaging of a fiber sample: brightfield, Brillouin shift, and linewidth images under different pump powers. Scale bars are 40 μm (brightfield) and 2 μm (Brillouin). Colorbars show Brillouin shift (${\varOmega }_{{\rm{B}}}/2{\rm{\pi }}$, see Supplementary Fig. S10 for a detailed analysis) and linewidth (${\varGamma }_{{\rm{B}}}/2{\rm{\pi }}$). White and gray regions of interest (ROIs) indicate regions with high Brillouin amplitude (see Supplementary Fig. S11), with overlaid text showing fitting precision for each. **d** (i) Brillouin microscopy of a phantom bead in agarose: brightfield, Brillouin shift, and linewidth images under varying pump powers. Scale bars: 5 μm. White ROI corresponds to agarose; gray ROI highlights part of the bead. **d** (ii) Brillouin microscopy of a HeLa cell: similar format and power range. Scale bars: 10 μm. White ROI marks culture medium around the HeLa cell

We first consider Brillouin imaging, implemented using a free-space optics setup (Fig. 5a) that builds on the configuration detailed in Fig. 4a, with two modifications. First, a high numerical aperture (NA = 0.7) objective is employed to focus light onto the cleaved end face of an optical fiber (SMF-28e, length = 1 km), which is mounted on a 3D piezo stage for precise scanning (see Supplementary Fig. S9 for the fiber mounting details). The detected SpBS signal results from the integration over the effective nonlinear length of the fiber, providing sufficient Brillouin gain to clearly visualize the SpBS noise and validate the SNR saturation effect in an imaging context. Second, a double-pass Fabry-Pérot interferometer is integrated to suppress amplified spontaneous emission noise (“Materials and Methods”; Supplementary Fig. S10). The SNR model derived from our framework for this imaging approach closely resembles Eq. (3), incorporating imaging-specific parameters such as $\bar{{P}_{{\rm{Sp}}}}$ and ${B}_{{\rm{m}}}$ (“Materials and Methods”). This model predicts a saturation behavior in SNR with increasing pump power (Fig. 5b, yellow curve), which is in good agreement with experimental data (yellow dots). The SNR dependency on ${B}_{{\rm{m}}}$ is also demonstrated to match the anticipation of our SNR model (Supplementary Fig. S12). More illustratively, Fig. 5c presents spatial maps of Brillouin shift and linewidth at pump powers from 1 dBm to 10 dBm in 3 dB increments. The precision of both parameters flattens between 7 dBm and 10 dBm, confirming the predicted SNR saturation regime where SpBS noise dominates. Conversely, below 7 dBm, the precision deteriorates rapidly with decreasing power, consistent with the shot-noise-limited regime observed in our direct detection experiments (Fig. 4b).

We further extend our framework to Brillouin microscopy, using the same setup (Fig. 5a) to investigate polydimethylsiloxane (PDMS) beads as a phantom and HeLa cells as a biological sample. While the underlying SNR model remains the same, the effective interaction lengths in both PDMS and cell samples are reduced to the micrometer scale. Combined with limited optical power delivery, this results in significantly weaker scattered signals compared to the kilometer-long fiber sample used in Brillouin imaging. Consequently, the system does not enter into the SpBS-noise-limited saturation regime, and its performance remains shot-noise-limited across the entire range of pump powers tested. This behavior is evidenced by the square-root scaling of Brillouin shift and linewidth precision with increasing pump power (Fig. 5d), and is similarly observed in measurements on a reference sample of double-distilled water (Fig. 5b, blue curve). These results align with previous reports of shot-noise-limited Brillouin microscopy^27,28,39^. Critically, however, our work introduces the first unified analytical framework that explicitly incorporates SpBS noise in Brillouin microscopy. This framework further elucidates the ultimate SNR limit that will arise when significantly higher optical powers are employed in future implementations.

#### Brillouin distributed optical fiber sensing

We next apply the framework to evaluate the performance of Brillouin optical time-domain reflectometry (BOTDR), a widely adopted sensing technique due to its single-ended operation, long sensing range, and large dynamic range^3–5,7,13,14^. We evaluate two implementations: using polarization-maintaining fiber (PMF) and standard single-mode fiber (SMF), each representing distinct noise regimes.

*BOTDR with polarization-maintaining fiber*. For PMF-based BOTDR, which eliminates polarization-induced fluctuations, the SNR of the single-pulse response ${r}_{{\rm{Sg}}}^{{\rm{PM}}}\left(z\right)$ at position *z* is derived as (see Supplementary Note S4 for derivation):

4 $$\begin{array}{l}\mathrm{SNR}\left\{{r}_{\mathrm{Sg}}^{\mathrm{PM}}\left(z\right)\right\}=\frac{{{{\mathcal{R}}}_{{\rm{p}}}}^{2}{P}_{\mathrm{Lo}}\overline{{P}_{\mathrm{Sp}}\left(z\right)}}{\sqrt{\frac{1}{2}\left(1+\frac{1}{{{SN}{R}_{\mathrm{Sp}}}^{2}}\right){{{\mathcal{R}}}_{{\rm{p}}}}^{4}{{P}_{\mathrm{Lo}}}^{2}{\overline{{P}_{\mathrm{Sp}}\left(z\right)}}^{2}+2{{{\mathcal{R}}}_{{\rm{p}}}}^{2}{P}_{\mathrm{Lo}}\overline{{P}_{\mathrm{Sp}}\left(z\right)}{{\sigma }_{e}}^{2}{B}_{\mathrm{BPF}}+{{\sigma }_{e}}^{4}{{B}_{\mathrm{BPF}}}^{2}}}\\ \le \frac{1}{\sqrt{\frac{1}{2}\left(1+\frac{1}{{{SN}{R}_{\mathrm{Sp}}}^{2}}\right)}}\end{array}$$

where $\overline{{P}_{{\rm{Sp}}}\left(z\right)}$ is the mean SpBS power at position $z$, proportional to the pump pulse duration and peak power, and ${B}_{{\rm{BPF}}}$ is the bandwidth of a bandpass filter (BPF) used before envelope extraction process (“Materials and Methods**”**). For meter-scale spatial resolutions, increasing pump power initially enhances the SNR; however, this improvement saturates as SpBS noise begins to dominate—especially near the fiber input or in short fiber spans, as is typical with PMF, the length of which cannot be extended significantly without compromising linear polarization due to practical manufacturing limitations^40^. Since optimized BOTDR systems require ${B}_{{\rm{m}}}\approx {B}_{{\rm{Sp}}}$, corresponding to ${SN}{R}_{{\rm{Sp}}}=1$, substituting which into Eq. (4) results in a saturated SNR (without averaging) of 1.

**Fig. 6:**
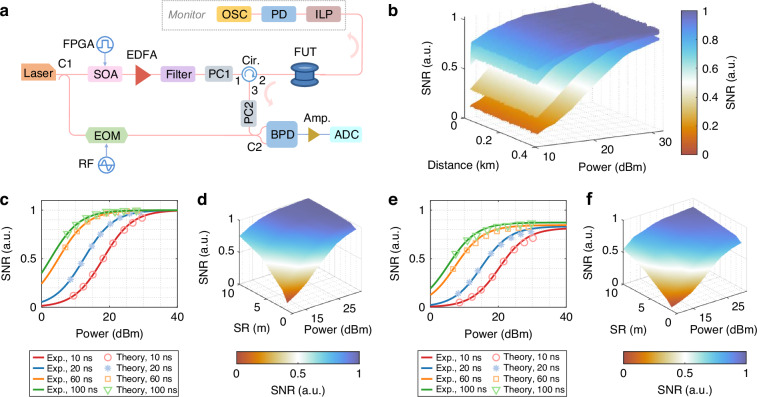
**Single-pulse BOTDR experimental configuration and results**. **a** Experimental configuration of single-pulse BOTDR based on PMF. FPGA: field-programmable gate array; SOA: semiconductor optical amplifier; EDFA: erbium-doped fiber amplifier; EOM: electro-optic modulator; ILP: in-line polarizer; PD: photodetector; OSC: oscilloscope. **b** Measured SNR surfaces representing SNR distribution along a 400 m-long PM fiber for spatial resolutions of 1 m, 2 m, 6 m, and 10 m, respectively. The different values of the SNR are represented by the different colors on the colorbar to the right. **c** Correspondence between the theoretically calculated and experimentally measured SNR values for a 400 m PMF, covering spatial resolutions of 1 m, 2 m, 6 m and 10 m. **d** Theoretical SNR behavior of a 400 m PMF under the combined influence of varying spatial resolutions and pump pulse powers. **e** Correspondence between the theoretically calculated and experimentally measured SNR values for a 1.9 km standard SMF, following the same parameter settings and adjustment logic as in (**c**). **f** Theoretical SNR behavior of a 1.9 km standard SMF under the combined influence of various spatial resolutions and various pump pulse powers

Experimental validation (Fig. 6a; “Materials and Methods”) uses a 400 m PMF and pump powers from 10 to 31 dBm in 3 dB intervals. Measured SNRs (Fig. 6b) show clear saturation trends consistent with theoretical predictions for various spatial resolutions (1 m, 2 m, 6 m, and 10 m). The detailed signal, noise and SNR evaluation for typical cases is illustrated in Supplementary Fig. S14. Note that larger spatial resolutions, which yield higher $\overline{{P}_{{\rm{Sp}}}\left(z\right)}$, require lower pulse peak powers to reach SNR saturation.

Due to the negligible attenuation in the 400 m PMF, the SNR remains nearly uniform along the fiber, allowing us to perform more accurate analysis by averaging the SNR over the fiber length (Fig. 6c), showing that the measured data (circle markers) closely match theoretical predictions (solid lines). These results directly challenge the conventional view that PMF-based BOTDR is inherently shot-noise-limited^3,41–46^. Furthermore, the SNR dependence on both spatial resolution and peak power (Fig. 6d) provides concrete guidance for optimizing BOTDR system to simultaneously guarantee a maximized SNR and a minimized energy consumption.

*BOTDR with standard single-mode fiber*. SMFs are widely used in practical deployments and support long-distance BOTDR operations spanning tens of kilometers^3,13,31,41–45,47–50^. However, they suffer from polarization noise caused by fiber birefringence and polarization scrambling^13,31,47–49^. In the absence of SpBS noise, recent analysis has shown that polarization noise alone imposes an SNR upper bound of 1 in SMF-based standard BOTDR system^13^. Here, we extend that model by incorporating our framework to include SpBS noise. The updated SNR expression (see Supplementary Note S5) becomes:

5 $$\begin{array}{l}\mathrm{SNR}\left\{{r}_{\mathrm{Sg}}^{\mathrm{SMF}}\left(z\right)\right\}=\frac{\frac{1}{2}{{{\mathcal{R}}}_{{\rm{p}}}}^{2}{P}_{\mathrm{Lo}}\overline{{P}_{\mathrm{Sp}}\left(z\right)}}{\sqrt{\frac{3{{SN}{R}_{\mathrm{Sp}}}^{2}\left({{k}_{\mathrm{Pol}}}^{2}+1\right)+{{k}_{\mathrm{Pol}}}^{2}+3}{24{{SN}{R}_{\mathrm{Sp}}}^{2}}{{{\mathcal{R}}}_{{\rm{p}}}}^{4}{{P}_{\mathrm{Lo}}}^{2}{\overline{{P}_{\mathrm{Sp}}\left(z\right)}}^{2}+{{{\mathcal{R}}}_{{\rm{p}}}}^{2}{P}_{\mathrm{Lo}}\overline{{P}_{\mathrm{Sp}}\left(z\right)}{{\sigma }_{e}}^{2}{B}_{\mathrm{BPF}}+{{\sigma }_{e}}^{4}{{B}_{\mathrm{BPF}}}^{2}}}\\ \le \sqrt{\frac{6{{SN}{R}_{\mathrm{Sp}}}^{2}}{3{{SN}{R}_{\mathrm{Sp}}}^{2}\left({{k}_{\mathrm{Pol}}}^{2}+1\right)+{{k}_{\mathrm{Pol}}}^{2}+3}}\end{array}$$

where the polarization scrambling factor ${k}_{{\rm{Pol}}} < 1$ quantifies the self-polarization-scrambling effect induced by the fiber birefringence (Supplementary Note S6). This factor reflects how much polarization fluctuations are averaged over the pump pulse duration—longer pulses result in smaller ${k}_{{\rm{Pol}}}$ (Supplementary Note S6). Notably, due to the average of polarization fluctuations along the sensing fiber, the expected signal (i.e., the numerator of Eq. (5)) is halved compared to Eq. (4).

While Eq. (5) retains the three noise terms from Eq. (4), the joint impact of SpBS noise and polarization noise modifies the first noise term. As a result, the SNR upper bound becomes a function of the spatial resolution, decreasing as the resolution increases (i.e., as ${k}_{{\rm{Pol}}}$ decreases). This revises the previous conclusion that polarization noise alone limits the SNR to 1 (ref.^13^), establishing a new theoretical SNR ceiling that is strictly less than 1 and spatial-resolution-dependent. Importantly, in long-distance scenarios where shot noise dominates at the fiber far-end, the original SNR model and conclusions remain valid^13^.

We experimentally verified the impact of SpBS noise by replicating the short-distance BOTDR measurements reported earlier^13^, using the same system configuration. The measured values of ${k}_{{\rm{Pol}}}$ for spatial resolutions of 1 m, 2 m, 6 m, and 10 m are identified to be 0.88, 0.83, 0.78 and 0.69, respectively. Excluding fiber regions unaffected by polarization noise (Supplementary Note S6), Fig. 6e compares experimentally measured SNRs with theoretical predictions, showing strong agreement and validating the accuracy of Eq. (5). The detailed signal, noise and SNR evaluation for typical cases is illustrated in Supplementary Fig. S14. Finally, Fig. 6f illustrates the dependence of theoretical SNR on both spatial resolution and pump power, revealing a downward-shifted SNR surface with a shallower slope compared to Fig. 6d, due to the joint impact of SpBS noise and polarization noise.

## Discussion

In summary, we have developed and experimentally validated a comprehensive theoretical framework to describe intensity fluctuations arising from spontaneous Brillouin scattering, by revisiting its physical formation mechanism and stochastic properties. This work deepens insight into SpBS noise and establishes the significance of accounting for it in spontaneous Brillouin-based systems, through four key contributions:

1. *Intuitive physical mechanism*. We present an analytical, stepwise explanation of the stochastic generation of Brillouin intensity fluctuations, as visualized in Fig. 1.
2. *Refined stochastic behavior*. Extending prior work that predicted a unity SNR for SpBS signals under ideal assumptions^32^, we incorporate practical system parameters into consideration. We show that the SNR critically depends on the phonon lifetime ($\tau$), measurement bandwidth (${B}_{{\rm{m}}}$), as well as sampling parameters (Fig. 2). This enables us to accurately quantify the impact of SpBS noise on given spontaneous Brillouin-based system.
3. *Experimental validation*. Using both coherent and direct detection experiments, we report the first quantitative analysis of SpBS intensity fluctuations in the spontaneous regime (gain coefficients $G\ll 21$)^34^, with $G\approx 0.23$ (Fig. 3) and $G\approx 3.89$ (Fig. 4), respectively. Our SNR predictions (Eqs. (2)–(5)) match well with experimental observations (Figs. 5, 6) across Brillouin imaging, microscopy, and distributed fiber sensing.
4. *Revised noise limit and optimization principle*. Our framework establishes SpBS noise as a fundamental noise floor in spontaneous Brillouin metrology. Once a system enters the SpBS-noise-limited regime, this intrinsic noise source can exceed conventional shot noise. We experimentally demonstrate this regime in both imaging (Fig. 5b,c) and distributed sensing (Fig. 6). This leads to a crucial optimization principle: due to the SpBS-noise-limited regime, there exists a well-defined pump power threshold beyond which increasing optical power no longer improves SNR. Instead, the performance can only be enhanced by extending the measurement time.

Beyond conventional systems, our framework generalizes to more advanced Brillouin sensing architectures and revises previously held assumptions about their performance limits. For example, polarization diversity coherent detection approach, employed to mitigate polarization noise in SMF-based BOTDR^49^, is shown to offer limited SNR improvement when SpBS noise is dominant (experimentally demonstrated in Supplementary Note S7). Similarly, while pulse coding techniques were developed to improve SNR^46,51^, their effectiveness in BOTDR is significantly constrained by the combined impact of SpBS and polarization noise (Supplementary Note S8). Nevertheless, such coding strategies regain relevance in Brillouin-integrated sensing and communication systems, where the background noise is higher^50^. These insights emphasize the necessity of incorporating SpBS noise into system design. Beyond its applicability to the coherent-detection BOTDR discussed above, we believe our framework can also be applied to direct-detection BOTDR (e.g., single-photon-detection BOTDR^14^), where phase and polarization fluctuations have negligible impact but the intensity fluctuations highlighted in this work may play a critical role. We also believe that similar methodology may be extended to distributed Raman sensors^3,52–54^, where spontaneous Raman scattering is the relevant signal to be measured, which is presumably affected by similar (but much faster) stochastic fluctuations of the spontaneous signal intensity.

Finally, while shot noise dominates in Brillouin microscopy—due to short interaction lengths and low power constraints for biological samples, preventing significant SpBS noise observation—our framework highlights the essential role of SpBS noise in high-power applications such as Brillouin spectroscopy^55,56^ and Brillouin Lidar systems^57,58^, where its impact has also been largely overlooked in the literature.

## Materials and methods

### Data generation for the 2D SNR map shown in Fig. 2f

The 2D SNR map in Fig. 2f is generated using 500 independent realizations of ${P}_{\rho }(n)$, a discrete-time representation of the power envelope of the spontaneous Brillouin field. Due to the equivalence in stochastic properties between $\vec{\rho }\left(t\right)$ and $\vec{{E}_{{\rm{Sp}}}}(t)$, ${P}_{\rho }(n)$ is used in place of ${P}_{{\rm{Sp}}}(n)$ for efficiency. Each realization is computed by squaring the discrete-time form of Eq.(S8), modeling $\vec{\rho }\left(t\right)$, and applying a 30 MHz digital low-pass filter. Key parameters are: carrier frequency: 220 MHz (arbitrarily chosen); phonon decay time: *τ* = 6 ns; delay time $\Delta t$ = 0.5 ns; sampling rate of 1 GSa/s; 35,000 samples per realization.

The resulting dataset (500 × 35,000 matrix) is used to evaluate SNR under different sampling conditions. Specifically, the normalized sampling interval ${T}_{{\rm{S}}}$/$\tau$ varies from 0 to 6 in steps of 1/6, and the number of sampling points ranges from 20 to 700 (step size: 10). For each parameter pair, the SNR is computed across all 500 realizations and averaged to yield each pixel value in the 2D map.

### Experimental setup and data processing

#### Coherent detection

The experimental configuration of coherent detection at 1550 nm is shown in Fig. 3a. A CW light from a distributed feedback laser operating at 1550 nm is split into pump and OLO branches using a PM optical coupler (C1). In the pump branch (upper arm), the CW light serves directly as the pump. A PM VOA controls the optical power of pump light before it is launched into a 400 m-long PM FUT via a non-PM circulator. A PC (PC1) preceding the circulator is adjusted to maximize the incident pump power, ensuring proper alignment of the incident polarization. The pump powers range from −25.5 dBm to 3.5 dBm in 1 dB step. In the OLO branch (lower arm), the CW light is modulated by an EOM driven by an RF signal, to produce a carrier-suppressed dual-sideband OLO wave. A narrowband optical filter selects the lower OLO sideband that interacts with the Stokes SpBS light. The optical power of the selected single-sideband OLO is set to 3 dBm. Another PC (PC2) aligns the polarization direction of the SpBS light with that of the OLO. At the receiver stage, the OLO and SpBS light are mixed via a 50/50 PM coupler (C2) and then detected by a BPD with a 400 MHz bandwidth. The resulting electrical beating signal after BPD is amplified by a low-noise electrical amplifier to overcome the ADC quantization noise, and then directly acquired at 1 GSa/s by the ADC (Fig. 3a(i)).

The beating signal is digitally post-processed to extract the Brillouin spectrum. Specifically, beat signals with a sampling duration of 10.24 μs (i.e., the number of sampling points ${N}_{{\rm{F}}}$ = 10,240) are continuously acquired over 10,000 realizations. Each beat signal undergoes an FFT, followed by modulus extraction, squaring, and normalization by ${N}_{{\rm{F}}}$, yielding the normalized Brillouin power spectral density (PSD). To maintain consistency with the units in Eq. (2), the obtained PSD is divided by the squared BPD conversion gain (${k}_{{\rm{VA}}}$) and by the gain factor of the electrical amplifier (${k}_{{\rm{amp}}}$). The result under a high pump power (2.5 dBm, 1.8 mW) is illustrated in Fig. 3b. The single-sided spectral range spans 500 MHz, determined by the sampling rate of 1 GSa/s. The light orange curve represents the Brillouin spectrum from a single realization, while the dark orange curve corresponds to the average of 10,000 spectra. The Brillouin peak is centered at approximately 220 MHz, consistent with the frequency offset between the OLO and SpBS signals. The noise spectrum shown in Fig. 3c is obtained by applying the same procedure in the absence of the pump light as that in Fig. 3b. For each pump power, the same post-processing procedure is applied as previously described, followed by subtraction of the noise baseline (Fig. 3c) to obtain a bias-free Brillouin spectrum. The mean peak amplitude across the 10,000 bias-free spectra is taken as the signal, the STD of the peak amplitude as the noise level, and their ratio as the SNR. These are respectively represented by the light blue circles in Fig. 3d, e, g. As a control experiment, CW laser output (without Brillouin scattering) with power matched to that of the SpBS light is used instead, yielding the pink asterisks in Fig. 3d, e, g. The parameters used to obtain the theoretical curves in Fig. 3d-g based on Eq. (2) are as follows: the responsivity of the photodiode ${{\mathcal{R}}}_{{\rm{p}}}$ = 0.95 A/W; the BPD conversion gain ${k}_{{\rm{VA}}}$ = 5000 V/A; the gain factor of the electrical amplifier ${k}_{{\rm{amp}}}$ = 316; the mean SpBS optical power (including Stokes and anti-Stokes components) $\bar{{P}_{{\rm{Sp}}}}={\beta }_{1}\left({{\rm{e}}}^{{\beta }_{2}{P}_{{\rm{p}}}}-1\right)$, where ${\beta }_{1}$ = 0.25, ${\beta }_{2}$ = 101.86, and ${P}_{{\rm{p}}}$ is the pump power; the dual-sideband OLO power ${P}_{{\rm{Lo}}}$ = 3 dBm; ${SN}{R}_{{\rm{Sp}}}$ = 1 (Supplementary Note S3); the STD of squared detection noise ${\rm{\pi }}{B}_{{\rm{Sp}}}{{\sigma }_{e}}^{2}/4$ = 2×10^-13^ A^2^. Note that the parameters may require slight empirical adjustment (typically within a factor of 1.4 of their theoretical values) to compensate for real-world system imperfections, such as unstable loss, non-ideal filter shape, and imprecise knowledge of ${B}_{{\rm{Sp}}}$. These adjustments ensure a closer alignment between the theoretical model and experimental results.

The pseudocode for the FFT-based post-processing described above is as follows:

**Input:**

number of pump powers ${{\boldsymbol{N}}}_{{\bf{p}}}$;

various pump powers ${{\boldsymbol{P}}}_{{\bf{p}}}$;

number of sampling points per realization ${{\boldsymbol{N}}}_{{\bf{F}}}$;

number of realizations ${{\boldsymbol{N}}}_{{\bf{Loop}}}$;

beating signal dataset of size ${\bf{2}}{{\boldsymbol{N}}}_{{\bf{Loop}}}\times {{\boldsymbol{N}}}_{{\bf{F}}}$, beat_noise, acquired in the absence of pump signal;

${N}_{{\rm{p}}}$ beating signal datasets ***beat_signal***, each of size ${{\boldsymbol{N}}}_{{\bf{Loop}}}{{\times }}{{\boldsymbol{N}}}_{{\bf{F}}}$, acquired under pump with varying powers;

BPD conversion gain ${{\boldsymbol{k}}}_{{\bf{VA}}}$;

gain factor of the electrical amplifier ${{\boldsymbol{k}}}_{{\bf{amp}}}$.

1 Load dataset ***beat_noise*** with size of

${\boldsymbol{2}}{{\boldsymbol{N}}}_{{\bf{Loop}}}\times {{\boldsymbol{N}}}_{{\bf{F}}}$ (${\boldsymbol{2}}{{\boldsymbol{N}}}_{{\bf{Loop}}}$ rows by ${{\boldsymbol{N}}}_{{\bf{F}}}$ columns);

2 **for** realization index i = 1:${\boldsymbol{2}}{{\boldsymbol{N}}}_{{\bf{Loop}}}$, do

3 *PSD_noise_i* = *Abs*(*FFT*(the *i*^th^ row of dataset *beat_noise*)^2^) / ${N}_{F}$ / ${{k}_{{\rm{VA}}}}^{2}$ /${k}_{{\rm{amp}}}$;

4 **end for**

5 Noise_baseline = Mean(PSD_noise_i over ${\bf{2}}{{\boldsymbol{N}}}_{{\boldsymbol{Loop}}}$ realizations);

6 **for** pump power index j = 1:${{\boldsymbol{N}}}_{{\bf{p}}}$, do

7 Load the j^**th**^ dataset beat_signal_j with size

of ${{\boldsymbol{N}}}_{{\bf{Loop}}}{{\times }}{{\boldsymbol{N}}}_{{\bf{F}}}$ (${{\boldsymbol{N}}}_{{\bf{Loop}}}$ rows by ${{\boldsymbol{N}}}_{{\bf{F}}}$ columns);

8 **for** realization index i = 1:${{\boldsymbol{N}}}_{{\bf{Loop}}}$, do

9 PSD_i = *Abs*(*FFT*(the i^**th**^ row of dataset ***beat_signal_j***)^2^) / ${{\boldsymbol{N}}}_{{\bf{F}}}$ / ${{{\boldsymbol{k}}}_{{\bf{VA}}}}^{{\boldsymbol{2}}}$ /${{\boldsymbol{k}}}_{{\bf{amp}}}$;

10 ***Biasfree_PSD_i*** = ***PSD_i*** - ***Noise_baseline***;

11 ***Peak_amplitude_i*** = *Peak*(***Biasfree_PSD_i***);

12 **end for**

13 ***Signal_j*** = *Mean*(***Peak_amplitude_i*** over ${{\boldsymbol{N}}}_{{\bf{Loop}}}$ realizations);

14 ***Noise_j*** = *STD*(***Peak_amplitude_i*** over ${{\boldsymbol{N}}}_{{\bf{Loop}}}$ realizations);

15 ***SNR_j*** = ***Signal_j*** / ***Noise_j***;

16 **end for**

**Output results:** mean signal ***Signal***, noise STD ***Noise***, and their ratio ***SNR*** of the bias-free Brillouin spectrum for each pump power.

Under a pump power of 2.5 dBm, a 30 μs-long beat signal is sampled at 1 GSa/s sampling rate with 500 realizations. A corresponding noise signal is recorded under identical conditions without pump light. Both the beat signal and noise undergo the same digital envelope detection as the simulated case (Fig. 2f). The true experimental envelope signal is derived by subtracting the noise envelope baseline from the directly obtained envelope (i.e., LPF output). The resulting envelope signal is evaluated for SNR with the same sampling intervals and sampling points as the simulated case. Each SNR value is obtained from 500 repeated measurements, yielding the results in Fig. 3h. The phonon energy decay time *τ* in the experiment is estimated to be 5.4 ns.

Additionally, an experimental envelope detection scheme shown by Fig. 3a(ii) is implemented to physically examine the impact of system bandwidth on SNR. In this approach, the amplified beating signal passes through a physical BPF (188 MHz center frequency, 50 MHz bandwidth), selecting the desired spectral components of both signal and noise. Then, a commercial logarithmic envelope detector (ED) is utilized to directly extract the temporal envelope signal. To explore the effects of system bandwidth, physical LPFs with two bandwidths of 50 MHz and 2 MHz are employed before ADC conversion. For each LPF configuration, the pump power is swept from 3.5 dBm to −25.2 dBm in 1 dB steps. At each pump power level, 10,000 realizations of the envelope signal (10 μs duration, sampled at 1 GSa/s) are recorded. Noise measurements (without pump light) are subsequently performed under identical sampling conditions. The unbiased envelope signal at each power level is obtained by subtracting the noise floor from the directly measured envelope signal. The mean values and fluctuation STD of the 10,000 unbiased envelope signals are extracted as signal mean and noise STD, respectively. Their ratio defines the SNR, as presented in Fig. 3i, j).

#### Direct detection

The experimental setup for direct detection of SpBS signal is shown in Fig. 4a. A frequency-locked, CW tapered amplifier laser operating at 780 nm is injected into one of the two output ports of a 1 × 2 50:50 PMF coupler. The 10 m-long PMF is connected to the input port of the coupler, and the backscattered SpBS signal is collected from the second port of the coupler and directed through a custom-built Rubidium vapor gas-cell filter, which provides ~50 dB extinction to suppress residual elastic scattering, and finally entering the VIPA-based spectrometer.

The VIPA-based spectrometer firstly includes a fiber collimator to collimate the SpBS light. The light is line-focused by a cylindrical lens. Different frequency components of the input light emerge at different angles and are spatially dispersed. The spectrally resolved signal is then imaged onto different position of a scientific complementary metal oxide semiconductor (sCMOS) camera. The Brillouin frequency shift and linewidth are extracted based on the known angular dispersion characteristics of the VIPA.

An example image acquired by the sCMOS camera is shown in Supplementary Fig. S7a, where the two resolved peaks (from top to bottom) correspond to the anti-Stokes and Stokes Brillouin signals of the 10 m-long fiber sample. A gray value intensity profile along the longitudinal direction of the selected region in the image is shown in Supplementary Fig. S7b. Lorentzian fitting is applied to each of the two Brillouin peaks to extract their spectral characteristics. The extracted Brillouin spectrum is corrected by subtracting the offset introduced by the camera’s background setting. To ensure consistency with the units in Eq. (3), the resulting grayscale spectrum is divided by the conversion gain coefficient of camera ${k}_{{\rm{IG}}}$. We analyze the STD of the Stokes peak intensity as a function of pump power to quantify the noise behavior and signal stability under varying excitation conditions.

The theoretical curves in Fig. 4b and c are obtained based on below parameters: the camera responsivity ${{\mathcal{R}}}_{{\rm{c}}}$ = 0.5 A/W, the camera conversion gain ${k}_{{\rm{IG}}}$ = 2 × 10^18^, the Brillouin linewidth ${B}_{{\rm{Sp}}}$ = 120 MHz, the noise-induced bias grey value is 1500, and the constant STD of camera background noise ${\sigma }_{{\rm{re}}}$ = 25/${k}_{{\rm{IG}}}$. In addition to these common parameters, in Fig. 4b, we use ${\beta }_{1}$ = 1.25 × 10^3^, ${\beta }_{2}$ = 6.47, and measurement bandwidth ${B}_{m}$ = 50 kHz, while in Fig. 4c we use ${\beta }_{1}$ = 2.24 × 10^4^, ${\beta }_{2}$ = 5.6, and ${B}_{{\rm{m}}}$ = 5 kHz.

#### Brillouin imaging and microscopy setup with VIPA system

The experimental setup for Brillouin imaging and microscopy at 780 nm is shown in Fig. 5a. A frequency-locked, CW tapered amplifier laser at 780 nm is spectrally filtered using an ASE-suppressed module (as described in Supplementary Fig. S10) and delivered to the main optical path via a PM fiber. The output laser is collimated using a fiber collimator and focused onto the sample mounted on a high-precision XYZ piezoelectric stage using a 60×, 0.7 NA objective lens. The backscattered Brillouin signal is collected along the same optical path, filtered by a Rubidium vapor cell (to suppress elastic scattering), and coupled into the VIPA-based spectrometer for spectral analysis.

A separate calibration arm, incorporating the standard water sample, is used to correct thermal drift of the VIPA dispersion. The Brillouin shift of water at room temperature is well known to be 5.07 GHz at 780 nm and serves as a reference for calibration. A computer-controlled shutter alternates between the main imaging arm and the calibration arm, enabling scheduled corrections throughout the imaging process to ensure measurement accuracy.

For fiber sample scanning in Fig. 5c, the XYZ three-axis input ports of the piezoelectric stage are independently controlled by three analog output channels from a data acquisition module, using a custom-built LabVIEW program. The data acquisition module also generates its internal clock signal of the created output voltage task, which serves as an external trigger for the sCMOS camera to ensure synchronized image acquisition. Due to the strong scattering signal from the fiber, the camera is operated in low gain mode. The scanning area is set to 18 µm × 18 µm with XY step size of 0.1 µm and an exposure time of 20 µs per pixel. To accommodate the limited scanning speed of the piezo stage, a dwell time of 10 ms is implemented via the LabVIEW program. Additionally, to ensure positional stability after each scan step, a 5 ms trigger delay is configured in Micro-Manager for the sCMOS camera.

For phantom bead and HeLa cell sample scanning in Fig. 5d(i) and Fig. 5d(ii), the sCMOS camera provides the trigger signal to the data acquisition module, enabling synchronized operation of sample scanning (via piezo control voltages) and image acquisition. The camera is operated in high gain mode to compensate for the weaker scattering signal from these samples. For the phantom bead sample, the scan area is 28 µm × 28 µm with XY step size of 0.25 µm and an exposure time of 20 ms per pixel. For the HeLa cell sample, the scanning area is 60 µm × 30 µm with the same step size and exposure time.

In addition to the parameters shared with Fig. 4b, c, the theoretical fiber curve in Fig. 5b is calculated using ${k}_{{\rm{IG}}}$ = 2 × 10^18^, ${\beta }_{1}$ = 1.39 × 10^3^, ${\beta }_{2}$ = 178, and ${B}_{{\rm{m}}}$ = 50 kHz; while for the water curve in Fig. 5b, the parameters are ${k}_{{\rm{IG}}}$ = 1 × 10^20^, ${\beta }_{1}$ = 6.63×10^8^, ${\beta }_{2}$ = 1.5 × 10^−4^, and ${B}_{{\rm{m}}}$ = 50 Hz.

#### Single-pulse BOTDR based on PMF

The experimental setup for single-pulse BOTDR based on PMF is shown in Fig. 6a. A CW light from a distributed feedback laser operating at 1550 nm is split into pump pulse and OLO branches by a PM coupler (C1). Unlike the fundamental coherent detection at 1550 nm, the pump pulse branch (upper arm) employs a high-extinction-ratio SOA driven by an FPGA to intensity-modulate the light into a single optical pulse. Before being launched into a 400 m-long PM fiber under test (FUT), the optical pulse successively passes through an EDFA (for peak power adjustment from 10 dBm to 31 dBm in 3 dB increment), a narrowband optical filter (for suppression of the broadband amplified spontaneous emission (ASE) noise), and a PC (PC1, to align the incident polarization with the principal axis of the PM FUT). The duration of pump pulse is set to 10 ns, 20 ns, 60 ns, and 100 ns, corresponding to the spatial resolutions of 1 m, 2 m, 6 m, and 10 m, respectively. After propagating through the PM FUT, the pump pulse light subsequently passes through an in-line polarizer before being detected by a PD with a 400 MHz bandwidth. The PD output is routed to a commercial oscilloscope (OSC) for monitoring the pulse shape and power. PC1 is adjusted to maximize the monitored pulse peak power, confirming proper alignment of the incident polarization. The OLO branch (lower arm) follows the same configuration as in Fig. 3a, except that both sidebands are retained. The optical power of dual-sideband OLO is set to approximately 3 dBm. PC2 aligns the polarization of the SpBS light with that of the OLO. The configuration used at the receiver stage is the same as that in Fig. 3a(i). The uncorrelated Stokes and anti-Stokes signals beat with the lower and upper sidebands of OLO, respectively, resulting in an electrical beating signal in the distance domain merged with zero-mean additive noise (detection noise). For each combination of pulse power and duration, 10,000 realizations are recorded within a 6.144 μs sampling window. Instead of a physical ED, digital post-processing is employed to extract the intensity envelope signal (similar with the method used for Fig. 3h). Specifically, the acquired 10,000 beating signals under every parameter set are first filtered by a digital BPF, with a bandwidth approaching the FWHM of the Brillouin spectrum. The filtered signal is then squared, followed by low-pass filtering with a bandwidth on the same order of magnitude as the FWHM of the Brillouin spectrum, to yield the intensity envelope signal. After correcting for the noise-induced bias by applying the identical processing procedure to the ADC output recorded without the pump pulse, the mean values and STD across 10,000 measurements are computed per pulse parameter configuration. The resulting SNR surface along the fiber is shown in Fig. 6b.

The parameters required in the theoretical calculations of Fig. 6 include: dual-sideband OLO power ${P}_{{\rm{Lo}}}$ = 3 dBm, backscattering coefficient ${k}_{{\rm{Sp}}}$ which is medium-dependent and the common value in silica is about -95 dB/m, effective group index of the propagating mode ${n}_{{\rm{eff}}}$ = 1.44 which is medium-dependent, mode group velocity in vacuum $c$ = 3 × 10^8 ^m/s, pump pulse duration ${D}_{{\rm{p}}}$ = 10, 20, 60, 100 ns. The pump pulse power ${P}_{{\rm{p}}}$, ranging from 10 dBm to 31 dBm with an interval of 3 dB, are merely theoretical reference power and may deviate from the actual values due to the influence of nonlinear effects (e.g., stimulated Brillouin scattering) and the limited performance of the devices (e.g., EDFA).

#### Single-pulse BOTDR based on SMF

The single-pulse BOTDR experiment based on SMF adopts a standard polarization-scrambled coherent detection scheme^13^. The FUT used is a 1.9 km-long SMF. The OLO power, pump pulse power and duration, as well as the detection components (BPD, amplifier, ADC), are identical to those in the PMF-based BOTDR setup. The electrical beating signal is recorded with a duration of 25.088 μs (25,088 samples at 1 GSa/s), repeated 10,000 times for each pulse configuration. The envelope extraction is performed digitally using the same processing and filtering parameters as in the PMF case.

### Sample preparations

#### Water sample preparation

The water sample is prepared by sandwiching 10 µL double-distilled water between two #1.5 coverslips, separated by a 120 µm thick imaging spacer (Grace Bio-Labs, SecureSeal).

#### PDMS beads sample preparation

To prepare polydimethylsiloxane (PDMS) beads embedded in agarose gel, 5 µL 1% (w/v) low-melting-point agarose solution in water is first deposited onto a #1.5 coverslip pre-mounted with a 120 µm thick imaging spacer. After gelation, PDMS beads are applied onto the gel surface, followed immediately by the addition of 7 µL of the same agarose solution. A second #1.5 coverslip is placed on top to seal the sample. This three-layer configuration effectively reduces pump light reflection.

#### Fiber sample preparation for Brillouin imaging

The fiber sample (see Supplementary Fig. S9 for details) is assembled by attaching a fiber adaptor (Thorlabs, SM1FCA) and a #1.5 coverslip with one imaging spacer with thickness of 120 µm. To prevent strong reflections of pump light at the fiber-air interface, an index-matching refractive index liquid (Cargille, Series AA, 1.414) is applied to fill the gap. A 1 km SMF is then slowly connected to a fiber adaptor to avoid the formation of air bubbles. The output power at the distal end of the fiber is monitored to verify the focal point location and determine the scan range and center.

#### Cells

HeLa cells (CBP60232, Cobioer) are cultured according to the American Type Culture Collection (ATCC) guidelines. Cells are maintained in DMEM (Gibco, 11960044) supplemented with 10% (v/v) FBS (Gibco, 30044333) and 100 U/mL penicillin-streptomycin (Gibco, 15140122). For imaging, cells are seeded at a density of 5000 cells/cm^2^ onto polyacrylamide (PAA)-coated imaging dishes (MATRIGEN, SV3510-EC-12) and allowed to adhere overnight.

## Supplementary information

Supplementary Information for A Framework for Spontaneous Brillouin Noise: Unveiling Fundamental Limits in Brillouin Metrology

## Data Availability

The dataset and code files that support the experiments of this study are available in Zenodo (10.5281/zenodo.17398879).

## References

[1] Boyd, R. W. Nonlinear Optics. 3rd edn. (Boston: Academic Press, 2008).

[2] Brillouin, L. Diffusion de la lumière et des rayons X par un corps transparent homogène: influence de l’agitation thermique. *Annales de. Phys.* **9**, 88–122 (1922).

[3] Hartog, A. H. An introduction to distributed optical fibre sensors (CRC Press, 2017).

[4] Kurashima, T. et al. Brillouin optical-fiber time domain reflectometry. *IEICE Trans. Commun.* **E76-B**, 382–390 (1993).

[5] Shimizu, K. et al. Coherent self-heterodyne detection of spontaneously Brillouin-scattered light waves in a single-mode fiber. *Opt. Lett.* **18**, 185–187 (1993). 19802078 10.1364/ol.18.000185

[6] Mizuno, Y. et al. Proposal of Brillouin optical correlation-domain reflectometry (BOCDR). *Opt. Express* **16**, 12148–12153 (2008). 18679490 10.1364/oe.16.012148

[7] Motil, A., Bergman, A. & Tur, M. [INVITED] State of the art of Brillouin fiber-optic distributed sensing. *Opt. Laser Technol.* **78**, 81–103 (2016).

[8] Mizuno, Y. et al. Ultrahigh-speed distributed Brillouin reflectometry. *Light Sci. Appl.* **5**, e16184 (2016). 30167136 10.1038/lsa.2016.184 PMC6059889

[9] Yang, F., Gyger, F. & Thévenaz, L. Intense Brillouin amplification in gas using hollow-core waveguides. *Nat. Photonics* **14**, 700–708 (2020). 33824683 10.1038/s41566-020-0676-z PMC7610518

[10] Merklein, M. et al. 100 years of Brillouin scattering: Historical and future perspectives. *Appl. Phys. Rev.* **9**, 041306 (2022).

[11] Huang, L. J. et al. Single-end hybrid Rayleigh Brillouin and Raman distributed fibre-optic sensing system. *Light Adv. Manuf.* **4**, 171–180 (2023).

[12] Youn, J. H., Kim, J. H. & Song, K. Y. Brillouin optical correlation domain analysis at MHz sampling rates using lock-in-free orthogonally polarized probe sidebands. *J. Lightwave Technol.* **42**, 6312–6317 (2024).

[13] Jin, S. M. et al. Analytical signal-to-noise ratio model on frequency-scanned Brillouin optical time-domain reflectometry. *J. Lightwave Technol.* **42**, 5786–5796 (2024).

[14] Romanet, M. et al. Extended-range and faster photon-counting Brillouin optical time domain reflectometer. *Optica* **12**, 564–569 (2025).

[15] Eggleton, B. J. et al. Brillouin integrated photonics. *Nat. Photonics* **13**, 664–677 (2019).

[16] Gyger, F. et al. Observation of stimulated Brillouin scattering in silicon nitride integrated waveguides. *Phys. Rev. Lett.* **124**, 013902 (2020). 31976733 10.1103/PhysRevLett.124.013902

[17] Yao, B. C. et al. Interdisciplinary advances in microcombs: bridging physics and information technology. *eLight* **4**, 19 (2024).

[18] Scarcelli, G. & Yun, S. H. Confocal Brillouin microscopy for three-dimensional mechanical imaging. *Nat. Photonics* **2**, 39–43 (2008). 10.1038/nphoton.2007.250 PMC2757783 19812712

[19] Scarcelli, G. et al. In vivo biomechanical mapping of normal and keratoconus corneas. *JAMA Ophthalmol.* **133**, 480–482 (2015). 25611213 10.1001/jamaophthalmol.2014.5641 PMC4698984

[20] Scarcelli, G. et al. Noncontact three-dimensional mapping of intracellular hydromechanical properties by Brillouin microscopy. *Nat. Methods* **12**, 1132–1134 (2015). 26436482 10.1038/nmeth.3616 PMC4666809

[21] Elsayad, K. et al. Mapping the subcellular mechanical properties of live cells in tissues with fluorescence emission–Brillouin imaging. *Sci. Signal.* **9**, rs5 (2016). 27382028 10.1126/scisignal.aaf6326

[22] Prevedel, R. et al. Brillouin microscopy: an emerging tool for mechanobiology. *Nat. Methods* **16**, 969–977 (2019). 31548707 10.1038/s41592-019-0543-3

[23] Margueritat, J. et al. High-frequency mechanical properties of tumors measured by Brillouin light scattering. *Phys. Rev. Lett.* **122**, 018101 (2019). 31012711 10.1103/PhysRevLett.122.018101

[24] Bailey, M. et al. Viscoelastic properties of biopolymer hydrogels determined by Brillouin spectroscopy: a probe of tissue micromechanics. *Sci. Adv.* **6**, eabc1937 (2020). 33127678 10.1126/sciadv.abc1937 PMC7608813

[25] Bevilacqua, C. et al. High-resolution line-scan Brillouin microscopy for live imaging of mechanical properties during embryo development. *Nat. Methods* **20**, 755–760 (2023). 36997817 10.1038/s41592-023-01822-1 PMC10172129

[26] Zhang, J. T. et al. Rapid biomechanical imaging at low irradiation level via dual line-scanning Brillouin microscopy. *Nat. Methods* **20**, 677–681 (2023). 36894684 10.1038/s41592-023-01816-z PMC10363327

[27] Kabakova, I. et al. Brillouin microscopy. *Nat. Rev. Methods Prim.* **4**, 8 (2024). 10.1038/s43586-023-00286-z PMC11465583 39391288

[28] Keshmiri, H. et al. Brillouin light scattering anisotropy microscopy for imaging the viscoelastic anisotropy in living cells. *Nat. Photonics* **18**, 276–285 (2024).

[29] Bevilacqua, C. & Prevedel, R. Full-field Brillouin microscopy based on an imaging Fourier-transform spectrometer. *Nat. Photonics* **19**, 494–501 (2025). 40352679 10.1038/s41566-025-01619-y PMC12058527

[30] Wang, S. et al. Study on the signal-to-noise ratio of Brillouin optical-time domain analyzers. *Opt. Express* **28**, 19864–19876 (2020). 32680057 10.1364/OE.393928

[31] Gao, X. et al. Impact of optical noises on unipolar-coded Brillouin optical time-domain analyzers. *Opt. Express* **29**, 22146–22158 (2021). 34265986 10.1364/OE.426655

[32] Boyd, R. W., Rza̧ewski, K. & Narum, P. Noise initiation of stimulated Brillouin scattering. *Phys. Rev. A* **42**, 5514–5521 (1990). 9904689 10.1103/physreva.42.5514

[33] Gaeta, A. L. & Boyd, R. W. Stochastic dynamics of stimulated Brillouin scattering in an optical fiber. *Phys. Rev. A* **44**, 3205–3209 (1991). 9906321 10.1103/physreva.44.3205

[34] Smith, R. G. Optical power handling capacity of low loss optical fibers as determined by stimulated Raman and Brillouin scattering. *Appl. Opt.* **11**, 2489–2494 (1972). 20119362 10.1364/AO.11.002489

[35] Beugnot, J. C. et al. Distributed Brillouin sensing with sub-meter spatial resolution: modeling and processing. *Opt. Express* **19**, 7381–7397 (2011). 21503049 10.1364/OE.19.007381

[36] Proakis, J. G. & Manolakis, D. G. Digital Signal Processing: Principles, Algorithms, and Applications. 3rd edn. (Upper Saddle River, Prentice Hall, 1996).

[37] Oxford Instruments. Sensitivity and Noise of CCD, EMCCD and sCMOS Sensors. at https://andor.oxinst.com/learning/view/article/sensitivity-and-noise-of-ccd-emccd-and-scmos-sensors. (2023).

[38] Oxford Instruments. Understanding read noise in sCMOS cameras. at https://andor.oxinst.com/learning/view/article/understanding-read-noise-in-scmos-cameras.

[39] Zhang, J. T. & Scarcelli, G. Mapping mechanical properties of biological materials via an add-on Brillouin module to confocal microscopes. *Nat. Protoc.* **16**, 1251–1275 (2021). 33452504 10.1038/s41596-020-00457-2 PMC8218248

[40] Noda, J., Okamoto, K. & Sasaki, Y. Polarization-maintaining fibers and their applications. *J. Lightwave Technol.* **4**, 1071–1089 (1986).

[41] Li, C. L. et al. SNR enhancement in Brillouin optical time domain reflectometer using multi-wavelength coherent detection. *Electron. Lett.* **48**, 1139–1141 (2012).

[42] Lu, Y. G. et al. Influence of non-perfect extinction ratio of electro-optic modulator on signal-to-noise ratio of BOTDR. *Opt. Commun.* **297**, 48–54 (2013).

[43] Lalam, N. et al. Performance improvement of Brillouin ring laser based BOTDR system employing a wavelength diversity technique. *J. Lightwave Technol.* **36**, 1084–1090 (2018).

[44] Lalam, N. et al. Performance analysis of Brillouin optical time domain reflectometry (BOTDR) employing wavelength diversity and passive depolarizer techniques. *Meas. Sci. Technol.* **29**, 025101 (2018).

[45] Bai, Q. et al. Enhancing the SNR of BOTDR by gain-switched modulation. *IEEE Photonics Technol. Lett.* **31**, 283–286 (2019).

[46] Soto, M. A., Bolognini, G. & Di Pasquale, F. Enhanced simultaneous distributed strain and temperature fiber sensor employing spontaneous Brillouin scattering and optical pulse coding. *IEEE Photonics Technol. Lett.* **21**, 450–452 (2009).

[47] van Deventer, M. O. & Boot, A. J. Polarization properties of stimulated Brillouin scattering in single-mode fibers. *J. Lightwave Technol.* **12**, 585–590 (1994).

[48] Zadok, A. et al. Vector analysis of stimulated Brillouin scattering amplification in standard single-mode fibers. *Opt. Express* **16**, 21692–21707 (2008). 19104601 10.1364/oe.16.021692

[49] Jostmeier, T. et al. Long-distance BOTDR interrogator with polarization-diverse coherent detection and power evaluation. Proceedings of Optical Fiber Sensors Conference 2020 Special Edition. T3.21 (Optica Publishing Group, 2020).

[50] Jin, S. M. et al. Single-channel integrated sensing and communication based on spontaneous Brillouin scattering. Proceedings of the 50th European Conference on Optical Communication. 1679-1682 (Frankfurt, VDE, 2024).

[51] Sun, X. Z. et al. Genetic-optimised aperiodic code for distributed optical fibre sensors. *Nat. Commun.* **11**, 5774 (2020). 33188171 10.1038/s41467-020-19201-1 PMC7666181

[52] Soto, M. A. & Di Pasquale, F. Distributed Raman sensing. in Handbook of Optical Fibers (ed Peng, G. D.) 1-55 (Singapore: Springer, 2018).

[53] Li, J. & Zhang, M. J. Physics and applications of Raman distributed optical fiber sensing. *Light Sci. Appl.* **11**, 128 (2022). 35525847 10.1038/s41377-022-00811-x PMC9079107

[54] Li, X. *et al*. Analyzing the coding gain of coded Raman optical time-domain reflectometry. *Proceedings of the 27th International Conference on Optical Fiber Sensors.* Th4.64 (Alexandria, Optica Publishing Group, 2022).

[55] Pan, X. G., Shneider, M. N. & Miles, R. B. Coherent Rayleigh-Brillouin scattering in molecular gases. *Phys. Rev. A* **69**, 033814 (2004). 10.1103/PhysRevLett.89.183001 12398594

[56] Vieitez, M. O. et al. Coherent and spontaneous Rayleigh-Brillouin scattering in atomic and molecular gases and gas mixtures. *Phys. Rev. A* **82**, 043836 (2010).

[57] Wang, Y. Q. et al. Brillouin scattering spectrum for liquid detection and applications in oceanography. *Opto Electron. Adv.* **6**, 220016 (2023).

[58] Zhou, Y. D. et al. Shipborne oceanic high-spectral-resolution lidar for accurate estimation of seawater depth-resolved optical properties. *Light Sci. Appl.* **11**, 261 (2022). 36055999 10.1038/s41377-022-00951-0 PMC9440025

