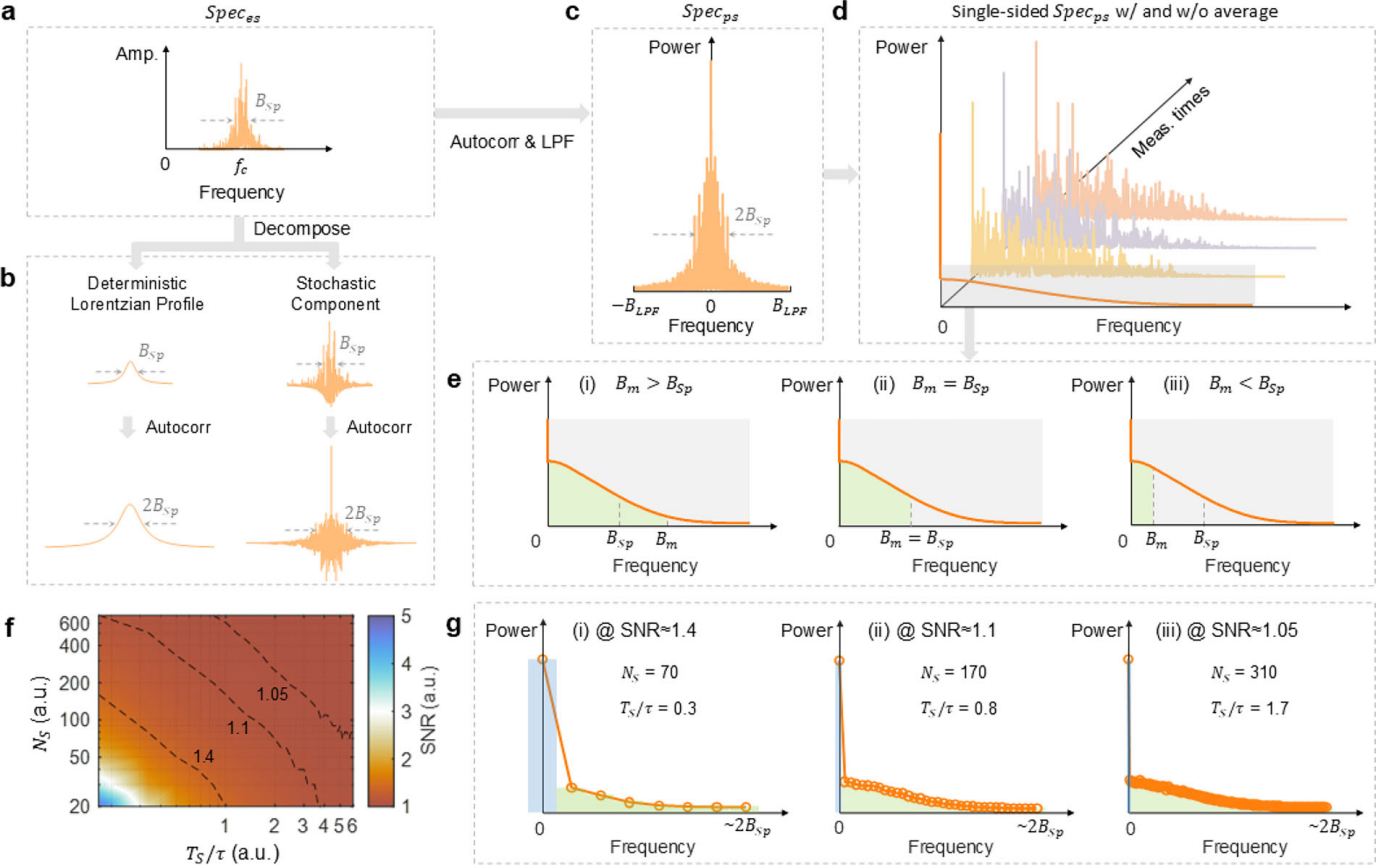# Correction: A framework for spontaneous Brillouin noise: unveiling fundamental limits in Brillouin metrology

**DOI:** 10.1038/s41377-026-02281-x

**Published:** 2026-04-16

**Authors:** Simeng Jin, Shuai Yao, Zhisheng Yang, Zixuan Du, Xiaobin Hong, Marcelo A. Soto, Jingjing Xie, Long Zhang, Fan Yang, Jian Wu

**Affiliations:** 1 State Key Laboratory of Information Photonics & Optical Communications, Beijing University of Posts and Telecommunications, Beijing, China; 2 Shanghai Institute of Optics and Fine Mechanics, Chinese Academy of Sciences, Shanghai, China; 3 Department of Electronics Engineering, Universidad Técnica Federico Santa María, Valparaíso, Chile; 4 School of Physical Science and Technology & State Key Laboratory of Advanced Medical Materials and Devices, ShanghaiTech University, Shanghai, China

**Keywords:** Microscopy, Nonlinear optics, Imaging and sensing

Correction to: Light: Science & Applications

10.1038/s41377-025-02115-2,published online 03 January 2026

After publication of this article, it was brought to our attention that Figure 2 is incorrect and should be replaced. The figures are shown below. The original paper has been updated.

The incorrect figure 2:
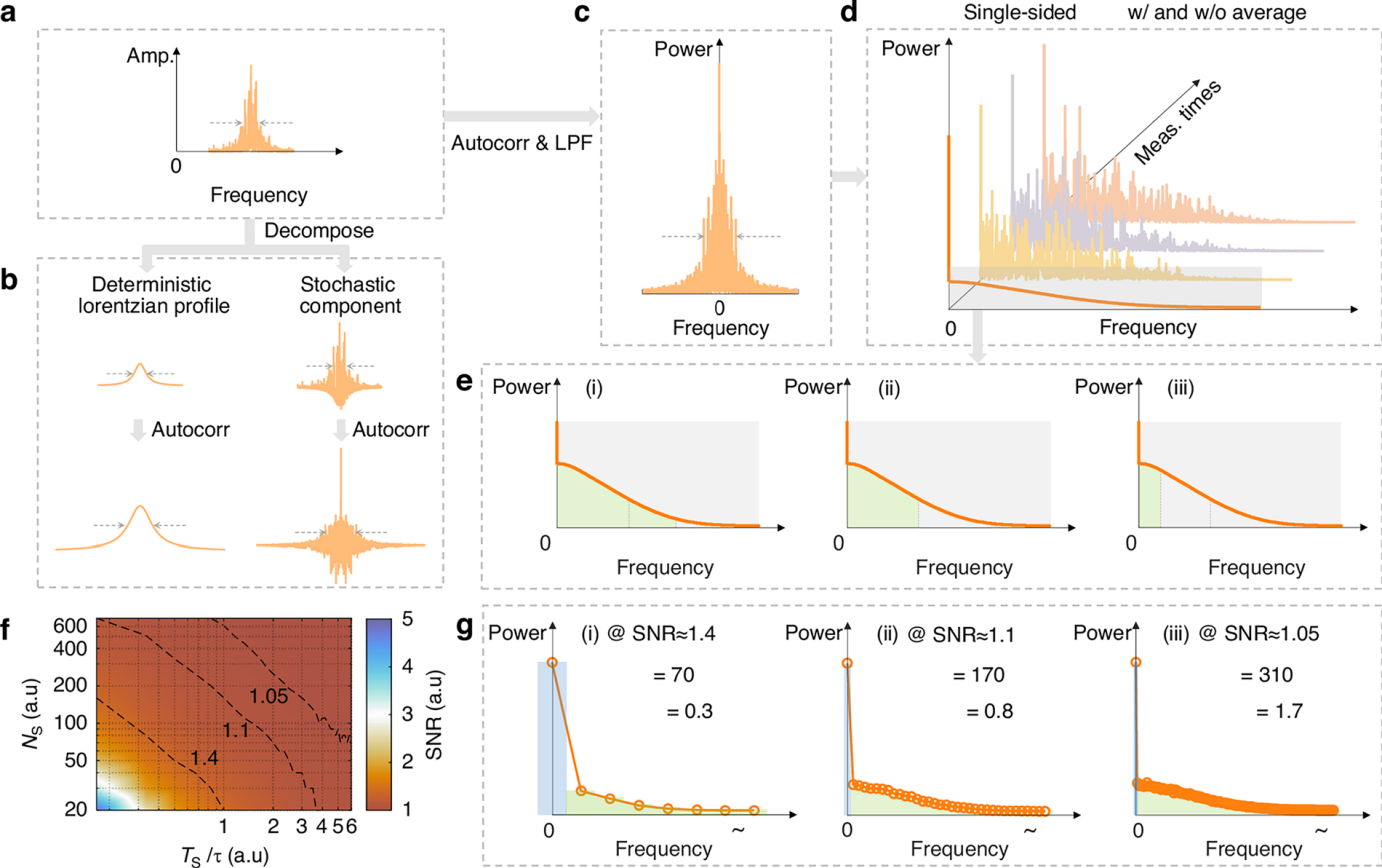


The correct figure 2: