# Supplementary material for: A framework for spontaneous Brillouin noise: unveiling fundamental limits in Brillouin metrology
Source: Light Sci Appl. 2026 Jan 3;15:44. doi: 10.1038/s41377-025-02115-2 (PMC12764778; doi:10.1038/s41377-025-02115-2)
Supplement: Supplementary file 1 — Supplementary Information for A Framework for Spontaneous Brillouin Noise: Unveiling Fundamental Limits in Brillouin Metrology [file 41377_2025_2115_MOESM1_ESM.pdf]

Supplementary Information for

A Framework for Spontaneous Brillouin Noise: Unveiling Fundamental Limits in Brillouin Metrology

Simeng Jin[1]†, Shuai Yao[2]†, Zhisheng Yang[1]*, Zixuan Du[2], Xiaobin Hong[1], Marcelo A. Soto[3], Jingjing Xie[4], Long Zhang[2], Fan Yang[2]*, Jian Wu[1]

[1] State Key Laboratory of Information Photonics & Optical Communications, Beijing University of Posts and Telecommunications, Beijing 100876, China
[2] Shanghai Institute of Optics and Fine Mechanics, Chinese Academy of Sciences 201800, Shanghai, China
[3] Department of Electronics Engineering, Universidad Técnica Federico Santa María, 2390123 Valparaíso, Chile
[4] School of Physical Science and Technology & State Key Laboratory of Advanced Medical Materials and Devices, ShanghaiTech University, Shanghai 201210, China

* Corresponding authors: zhisheng.yang@bupt.edu.cn; yang@siom.ac.cn.
† These authors contributed equally to this work.

This PDF file includes:

Notes S1 to S8
Figs. S1 to S18
Tables S1 to S4
References

Contents

Note S1: Physical derivation
Stepwise description for physical formation mechanism of spontaneous Brillouin scattering intensity fluctuations 4

Note S2: SpBS fluctuation characteristics
Mathematical investigation of the SNR in SpBS intensity fluctuations 6
1. Analytically validation on the conventional assumption of SNR=1 6
2. Updating the SNR to be B_m dependent 9

Note S3: FFT-based coherent detection
Modelling the SNR for FFT-based coherent detection 10

Note S4: PMF-based single-pulse BOTDR
Modelling the SNR of single-pulse BOTDR using polarization-maintaining fiber 17

Note S5: SMF-based single-pulse BOTDR
Modelling the SNR of single-pulse BOTDR using standard single-mode fiber 21

Note S6: Self-polarization-scrambling effect
Analysis of the self-polarization-scrambling effect 23
1. Experimental quantification of k_Pol for different spatial resolutions (SRs) 23
2. Layered SNR behavior with a polarization scrambler (PSc) 23

Note S7: PDCR-based BOTDR
BOTDR based on polarization diversity coherent receiver 24

Note S8: SMF-based coded-pulse BOTDR
Modelling and experimental verification of the SNR in coded-pulse BOTDR using standard SMF 25
1. SNR model of coded-pulse BOTDR 25
2. Experimental verification 30

Fig. S1: Time–frequency characterization across different sampling conditions 32

Fig. S2: Schematic diagram of single-frequency and Lorentzian spectra 33

Fig. S3: Schematic diagram illustrating the equivalent and actual noise power spectral density (PSD) 34

Fig. S4: Spectral interpretation on SNR behavior of FFT-based coherent detection 35

Fig. S5: Results of anti-Stokes signal 36

Fig. S6: Relative contributions of individual noise terms as functions of pump power and system gain G 37

Fig. S7: Raw data and Lorentzian fit of the Brillouin signal from a 10 m fiber acquired via direct detection 38

Fig. S8: Quantitative measurement of fiber frequency shift using VIPA with grating spectrometer 39

Fig. S9: Schematic of the fiber sample used for Brillouin imaging 40

Fig. S10: Setup of the ASE-filtered module with temperature-controlled FP etalon....................41

Fig. S11: Brillouin signal amplitude of a fiber sample using the VIPA spectrometer..................42

Fig. S12: Brillouin signal SNR analysis of a fiber sample using the VIPA spectrometer 43

Fig. S13: Noise standard deviations along the sensing distance 44

Fig. S14: Supplementary results for single-pulse BOTDR 45

Fig. S15: Simulated fluctuation characteristics of $\cos^2(\theta)$ affected by the self-polarization-scrambling effect..46

Fig. S16: Experimental results of the analysis on the self-polarization-scrambling effect...........47

Fig. S17: Experimental results of BOTDR based on polarization diversity coherence receiver (PDCR) 48

Fig. S18: SNR behaviour of coded-pulse BOTDR along a 50 km standard SMF........................49

Table S1: Expectation values and variances of the three terms in Eq. (S46) 50

Table S2: Expectation values and variances of the three terms in Eq. (S78) 51

Table S3: Expectation values and variances of the three terms in an expression similar to Eq. (S78) for the SMF case 52

Table S4: Expectation values and variances of the three terms in an expression similar to Eq. (S78) for the coded-pulse case..53

References...54

Supplementary Note S1. Stepwise description for physical formation mechanism of spontaneous Brillouin scattering intensity fluctuations

This supplementary note details the derivation of the physical formation mechanism of spontaneous Brillouin scattering (SpBS) intensity fluctuations. The analysis is based on solving the standard three-wave coupled partial differential equations for SpBS[1-3]:

$$\left(\frac{1}{v_\mathrm{g}}\frac{\partial}{\partial t}+\frac{\partial}{\partial z}+\frac{\alpha}{2}\right)E_\mathrm{P}(z,t)=0 \tag{S1}$$

$$\left(\frac{1}{v_g}\frac{\partial}{\partial t}-\frac{\partial}{\partial z}+\frac{\alpha}{2}\right)E_\mathrm{S}(z,t)=\mathrm{j}\kappa\rho^*(z,t)E_\mathrm{P}(z,t) \tag{S2}$$

$$\frac{\partial\rho(z,t)}{\partial t}+\frac{1}{2}\Gamma\rho(z,t)=f(z,t) \tag{S3}$$

where $E_\mathrm{P}(\cdot)$, $E_\mathrm{S}(\cdot)$, and $\rho(\cdot)$ represent the complex amplitudes of the pump light, the Stokes SpBS light and the acoustic wave (density wave), respectively, as functions of position z and time t. The parameters include the group velocity $v_\mathrm{g}=c/n_\mathrm{eff}$, where c is the speed of light in a vacuum and n_eff is the effective group index; α is the attenuation coefficient; j is the imaginary unit; κ is the electrostrictive coupling coefficient; Γ is the phonon decay rate; and $f(z,t)$ is the Langevin noise source responsible for thermal (or quantum) excitation of acoustic waves. The noise $f(z,t)$ is modeled as a complex, zero-mean Gaussian white noise process in both space and time. Here we follow the common assumption that Langevin noise is δ-correlated in time and space, since the underlying microscopic fluctuations occur on extremely short time scales (up to THz) compared with the nanosecond temporal resolution, and on atomic spatial scales (~10^{-10} m in silica) that are much smaller than the micrometer spatial sampling scales (~10^{-6} m) relevant to our analysis.

To intuitively and mathematically analyze SpBS fluctuations, we discretize the light–matter interaction length into N_z ($\rightarrow\infty$) short segments (**Fig. 1** in main text), each with length of Δz ($\rightarrow +0$). The k-th segment is located at $z_k=k\Delta z$, with $k\in[1,N_z]$. In each segment, local thermally activated vibrations are modeled by a stochastic process of Langevin noise $f(t)$, which is expressed as a series of N_t ($\rightarrow\infty$) Dirac delta functions $\delta(\cdot)$ separated by time intervals Δt ($\rightarrow +0$) (**Fig. 1**(i) in main text):

$$f(z_k,t)=A^f(z_k,t)\mathrm{e}^{\mathrm{j}\varphi^f(z_k,t)}=\sum_{i=1}^{N_t}A^f(z_k,t_i)\Delta t\cdot\delta(t-t_i)\cdot\mathrm{e}^{\mathrm{j}\varphi^f(z_k,t_i)} \tag{S4}$$

where $A^f(\cdot)$ and $\varphi^f(\cdot)$ stand for the random amplitude and phase of the Langevin noise, respectively; $t_i=i\Delta t$ is the time of the i-th excitation. Since the acoustic wave propagates much slower than light, it is treated as a local, non-propagating time-dependent process. Substituting Eq. (S4) into Eq. (S3), the local acoustic wave excited within the certain segment z_k satisfies:

$$\frac{\partial\rho(z_k,t)}{\partial t}+\frac{1}{2}\Gamma\rho(z_k,t)=\sum_{i=1}^{N_t}A^f(z_k,t_i)\Delta t\cdot\delta(t-t_i)\cdot\mathrm{e}^{\mathrm{j}\varphi^f(z_k,t_i)} \tag{S5}$$

Applying Laplace transformation[3] to Eq. (S5) yields:

$$s\tilde{\rho}(z_k,s)+\frac{1}{2}\Gamma\tilde{\rho}(z_k,s)=\sum_{i=1}^{N_t}A^f(z_k,t_i)\Delta t\cdot\mathrm{e}^{-t_is}\cdot\mathrm{e}^{\mathrm{j}\varphi^f(z_k,t_i)} \tag{S6}$$

where the ~ hat denotes the Laplace transform; s is the Laplace variable. Rearranging Eq. (S6) yields:

$$\tilde{\rho}(z_k,s)=\frac{\sum_{i=1}^{N_t}A^f(z_k,t_i)\Delta t\cdot\mathrm{e}^{-t_is}\cdot\mathrm{e}^{\mathrm{j}\varphi^f(z_k,t_i)}}{s+\frac{1}{2}\Gamma} \tag{S7}$$

Performing the inverse Laplace transformation to Eq. (S7), the time-domain solution for the local acoustic wave complex amplitude at z_k is obtained:

$$\rho(z_k, t) = \sum_{i=1}^{N_t} A^f(z_k, t_i)\Delta t \cdot \mathrm{e}^{-\frac{1}{2}\Gamma(t-t_i)} u(t-t_i) \cdot \mathrm{e}^{\mathrm{j}\varphi^f(z_k,t_i)} \tag{S8}$$

where $u(\cdot)$ conventionally represents the Heaviside unit step sequence. Including the carrier frequency ω_ρ of the acoustic carrier wave, the full complex acoustic field is:

$$\vec{\rho}(z_k, t) = \rho(z_k, t) e^{\mathrm{j}\omega_\rho t} = \sum_{i=1}^{N_t} A^f(z_k, t_i)\Delta t \cdot \mathrm{e}^{-\frac{1}{2}\Gamma(t-t_i)} u(t-t_i) \cdot \mathrm{e}^{\mathrm{j}\varphi^f(z_k,t_i)} \mathrm{e}^{\mathrm{j}\omega_\rho t} \tag{S9}$$

Eq. (S9) shows that $\vec{\rho}(t)$ can be represented on the time scale as the superposition of N_t waves with time delays at step of Δt (**Fig. 1**(ii) in main text): each wave shares the same angular frequency ω_ρ, but featuring random initial amplitude $A^f(\cdot)\Delta t$ and phase $\varphi^f(\cdot)$ that are both dictated to those of corresponding Langevin noise components given by Eq. (S4). In addition, the amplitude envelope of each wave exhibits an exponential decay trend, with time constant determined by the acoustic lifetime of a certain material.

Substituting Eq. (S9) into Eq. (S2), we derive the total SpBS light field returning to the pump-launching input (first segment, z_1):

$$\overrightarrow{E_{\mathrm{Sp}}}(t) \overset{\mathrm{def}}{=} \overrightarrow{E_{\mathrm{S}}}(z_1, t) = \mathrm{j}\kappa_1 \sum_{k=1}^{N_\mathrm{z}} \vec{\rho}^* \left(z_k, t - \frac{z_k}{v_g}\right) \overrightarrow{E_{\mathrm{P}}} \left(z_k, t - \frac{z_k}{v_\mathrm{g}}\right) \tag{S10}$$

where $\kappa_1 = \sqrt{P_\mathrm{P}/A_\mathrm{eff}}\kappa$, P_P being the power of incident pump light and A_eff is the effective mode area; $E_\mathrm{P}(\cdot)$ is the pump light reaching z_k. Assuming a typical rectangular pump pulse:

$$\overrightarrow{E_{\mathrm{P}}}(z_k, t) = E_\mathrm{P}^0 \Delta t \left[u\left(t - \frac{z_k}{v_\mathrm{g}}\right) - u\left(t - D_\mathrm{P} - \frac{z_k}{v_\mathrm{g}}\right)\right] \mathrm{e}^{-\mathrm{j}\omega_\mathrm{P} t} \tag{S11}$$

where E_P^0, D_P and ω_P denote amplitude, duration, and angular frequency of the incident pump light, respectively. Substituting into Eq. (S10) gives:

$$\overrightarrow{E_{\mathrm{Sp}}}(t) = \mathrm{j}\kappa_1 E_\mathrm{P}^0 \Delta t \sum_{k=1}^{N_\mathrm{P}} \vec{\rho}^* \left(z_k, t - \frac{z_k}{v_\mathrm{g}}\right) \mathrm{e}^{-\mathrm{j}\left[\omega_\mathrm{P}\left(t - \frac{z_k}{v_\mathrm{g}}\right)\right]} \tag{S12}$$

where $N_\mathrm{P} = D_\mathrm{P} v_\mathrm{g}/\Delta z + 1$ is the number of interacting segments. Eq. (S12) straightforwardly indicates that $\overrightarrow{E_{\mathrm{Sp}}}(t)$ is a superposition of time-delayed acoustic responses weighted by the pump field, as shown by **Fig. 1** in the main text.

Substituting Eq. (S9) into Eq. (S12) gives rises to the full expression of SpBS light field:

$$\overrightarrow{E_{\mathrm{Sp}}}(t) = \mathrm{j}\kappa_1 E_\mathrm{P}^0 \Delta t \sum_{k=1}^{N_\mathrm{P}} \sum_{i=1}^{N_t} A^f(z_k, t_i)\Delta t \cdot \mathrm{e}^{-\frac{1}{2}\Gamma\left(t - \frac{z_k}{v_\mathrm{g}} - t_i\right)} u(t - t_i) \mathrm{e}^{-\mathrm{j}\varphi^f(z_k,t_i)} \mathrm{e}^{-\mathrm{j}\left[\omega_{\mathrm{Sp}}\left(t - \frac{z_k}{v_\mathrm{g}}\right)\right]} \tag{S13}$$

where $\omega_{\mathrm{Sp}} = \omega_\mathrm{P} - \omega_\rho$ denotes the angular frequency of $\overrightarrow{E_{\mathrm{Sp}}}(t)$. Though Eq. (S13) is not immediately intuitive, it forms the basis for stochastic analysis of SpBS intensity in **Note S2**.

Supplementary Note S2. Mathematical investigation of the SNR in SpBS intensity fluctuations

The seminal study correctly attributes spontaneous Brillouin scattering (SpBS) intensity fluctuations to the linear amplification of light scattered by thermally generated phonons. Consequently, the signal-to-noise ratio (SNR) is interpreted as unity – reflecting the stochastic equivalence between thermally driven phonon vibrations and thermal light, both of which exhibit a unity SNR[4,5].

Building on this physical insight, we present a rigorous mathematical derivation of the SpBS SNR based exclusively on the analytical expressions derived in **Note S1**, without resorting to approximations or assuming any particular intensity distribution. By integrating a time-domain derivation with a complementary frequency-domain analysis, we offer a comprehensive framework for understanding the statistical properties governing SpBS intensity fluctuations.

1. Analytically validation on the conventional assumption of SNR=1

The derivation on the statistics of $P_{\mathrm{Sp}}(t)$ starts from manipulating $\vec{\rho}(z_k,t)$ expressed by Eq. (S9), due to its close relation with $\overrightarrow{E_{\mathrm{Sp}}}(t)$ as expressed by Eq. (S12). First, the negative exponential function in Eq. (S9) is Fourier expanded to n terms as[6]:

$$\mathrm{e}^{-\frac{1}{2}\Gamma(t-t_i)} = \sum_{x=1}^{n} A_x^{\mathrm{e}} \mathrm{e}^{\mathrm{j}[\omega_x^{\mathrm{e}}(t-t_i)+\varphi_x^{\mathrm{e}}]} = \sum_{x=1}^{n} A_x^{\mathrm{e}} \mathrm{e}^{\mathrm{j}(\omega_x^{\mathrm{e}}t-\omega_x^{\mathrm{e}}t_i+\varphi_x^{\mathrm{e}})} \tag{S14}$$

where A_x^{e}, φ_x^{e}, and ω_x^{e} represent the coefficient, phase, and angular frequency of the x-th term after expansion, respectively. Substituting Eq. (S14) into Eq. (S9), the time-delayed conjugate expression of the acoustic wave can be written as:

$$\begin{aligned}\vec{\rho}^*\left(z_k, t-\frac{z_k}{v_{\mathrm{g}}}\right) &= \sum_{i=1}^{N_t}\left\{A^f(z_k,t_i)\Delta t\,\mathrm{e}^{-\mathrm{j}\varphi^f(z_k,t_i)}\mathrm{e}^{-\mathrm{j}\omega_\rho\left(t-\frac{z_k}{v_{\mathrm{g}}}\right)}\sum_{x=1}^{n}A_x^{\mathrm{e}}\mathrm{e}^{-\mathrm{j}\left[\omega_x^{\mathrm{e}}\left(t-\frac{z_k}{v_{\mathrm{g}}}\right)-\omega_x^{\mathrm{e}}t_i+\varphi_x^{\mathrm{e}}\right]}\right\}\\ &= \sum_{i=1}^{N_t}\left\{A^f(z_k,t_i)\Delta t\sum_{x=1}^{n}A_x^{e}\mathrm{e}^{-\mathrm{j}\left\{(\omega_\rho+\omega_x^{\mathrm{e}})t+\left[\varphi_x^{\mathrm{e}}+\varphi^f(z_k,t_i)-\omega_x^{\mathrm{e}}t_i-(\omega^\rho+\omega_x^{\mathrm{e}})\frac{z_k}{v_{\mathrm{g}}}\right]\right\}}\right\}\end{aligned} \tag{S15}$$

Let $A_{ki}^f = A^f(z_k,t_i)\Delta t$, $\Omega_x = \omega_\rho + \omega_x^e$, and $\phi_{kix} = \varphi_x^e + \varphi^f(z_k,t_i) - \omega_x^e t_i - \Omega_x \frac{z_k}{v_{\mathrm{g}}}$, and by switching the order of summation, Eq. (S15) can be rewritten as:

$$\vec{\rho}^*\left(z_k, t-\frac{z_k}{v_{\mathrm{g}}}\right) = \sum_{i=1}^{N_t}\left\{A_{ki}^f\sum_{x=1}^{n}A_x^{\mathrm{e}}\mathrm{e}^{-\mathrm{j}(\Omega_x t+\phi_{kxi})}\right\} = \sum_{x=1}^{n}\left\{A_x^e\sum_{i=1}^{N_t}A_{ki}^f\mathrm{e}^{-\mathrm{j}(\Omega_x t+\phi_{kix})}\right\} \tag{S16}$$

Let F_{kx} and Φ_{kx} denote the amplitude and phase variables after the first summation, respectively, as[5]:

$$F_{kx}{}^2 = \left[A_x^e\Sigma_{i=1}^{N_t}A_{ki}^f\cos(\phi_{kix})\right]^2 + \left[A_x^e\Sigma_{i=1}^{N_t}A_{ki}^f\sin(\phi_{kix})\right]^2 \tag{S17}$$

$$\Phi_{kx} = \arctan\left[\frac{A_x^e\Sigma_{i=1}^{N_t}A_{ki}^f\sin(\phi_{kix})}{A_x^e\Sigma_{i=1}^{N_t}A_{ki}^f\cos(\phi_{kix})}\right] \tag{S18}$$

leading to a simplified version of Eq. (S16),

$$\vec{\rho}^*\left(z_k, t-\frac{z_k}{v_{\mathrm{g}}}\right) = \sum_{x=1}^{n}F_{kx}\mathrm{e}^{-\mathrm{j}(\Omega_x t+\Phi_{kx})} \xrightarrow{\text{Real}} \sum_{x=1}^{n}F_{kx}\cos(\Omega_x t+\Phi_{kx}) \tag{S19}$$

where the indicator 'Real' located above the arrow denotes taking the real part. Note that Eq. (S19) is similar to the expression of thermal light[5], but differs in that it incorporates a variable carrier frequency Ω_x, rather than a fix carrier frequency in the case of thermal light. This operation may lead to a complex derivation procedure; however, its purpose is to provide a rigorous, step-by-step derivation of the statistical properties of SpBS intensity fluctuations, rather than to pursue the simplest possible formulation.

By substituting Eq. (S19) into Eq. (S12), and switching the order of summation, $\overrightarrow{E_{\mathrm{Sp}}}(t)$ is further stated as:

$$\overrightarrow{E_{\mathrm{Sp}}}(t) = \epsilon \sum_{k=1}^{N_{\mathrm{P}}} \left\{ \sum_{x=1}^{n} F_{kx} \cos(\Omega_x{}' t + \Phi_{kx}{}') \right\} = \epsilon \sum_{x=1}^{n} \left\{ \sum_{k=1}^{N_{\mathrm{P}}} F_{kx} \cos(\Omega_x{}' t + \Phi_{kx}{}') \right\} \quad \text{(S20)}$$

where $\Omega_x{}' = \Omega_x + \omega_{\mathrm{P}}$; $\Phi_{kx}{}' = \Phi_{kx} - \omega_{\mathrm{P}} \frac{z_k}{v_{\mathrm{g}}}$; N_{P} is the number of segments within the pump light duration; $\epsilon = \mathrm{j}\kappa_1 E_{\mathrm{P}}^0 \Delta t$ is a constant with a given pump light. Similar to the calculation procedure from Eqs. (S17)-(S18) to Eq. (S19), we define ξ_x and ψ_x as the amplitude and phase variables after the first summation of Eq. (S20):

$$\xi_x{}^2 = \left[\Sigma_{k=1}^{N_P} F_{kx} \cos(\Phi_{kx}{}')\right]^2 + \left[\Sigma_{k=1}^{N_P} F_{kx} \sin(\Phi_{kx}{}')\right]^2 \quad \text{(S21)}$$

$$\psi_x = \arctan\left[\frac{\Sigma_{k=1}^{N_P} F_{kx} \sin(\Phi_{kx}{}')}{\Sigma_{k=1}^{N_P} F_{kx} \cos(\Phi_{kx}{}')}\right] \quad \text{(S22)}$$

resulting in:

$$\overrightarrow{E_{\mathrm{Sp}}}(t) = \epsilon \sum_{x=1}^{n} \xi_x \cos(\Omega_x{}' t + \psi_x) \quad \text{(S23)}$$

Eq. (S19) and Eq. (S23) show exactly the same formation, indicating that $\overrightarrow{E_{\mathrm{Sp}}}(t)$ and $\vec{\rho}(t)$ actually share same statistical properties. In most practical Brillouin systems, the power envelope of $\overrightarrow{E_{\mathrm{Sp}}}(t)$, i.e., $P_{\mathrm{Sp}}(t)$, is typically analyzed, which is mathematically equivalent to take low-pass filtering on the squared $\overrightarrow{E_{\mathrm{Sp}}}(t)$ that can be expressed as:

$$\overrightarrow{E_{\mathrm{Sp}}}(t)^2 = \epsilon^2 \left\{ \sum_{x=1}^{n} \xi_x \cos(\Omega_x{}' t + \psi_x) \right\}^2$$

$$= \epsilon^2 \left\{ \sum_{x=1}^{n} \xi_x{}^2 \cos^2(\Omega_x{}' t + \psi_x) + \sum_{x \neq y}^{\frac{n(n-1)}{2}} 2\xi_x \xi_y \cos(\Omega_x{}' t + \psi_x) \cos(\Omega_y{}' t + \psi_y) \right\}$$

$$= \epsilon^2 \sum_{x=1}^{n} \frac{1}{2} \xi_x{}^2 [\cos(2\Omega_x{}' t + 2\psi_x) + 1] +$$

$$+\epsilon^2 \sum_{x \neq y}^{\frac{n(n-1)}{2}} \xi_x \xi_y \{\cos[(\Omega_x{}' + \Omega_y{}')t + (\psi_x + \psi_y)] + \cos[(\Omega_x{}' - \Omega_y{}')t - (\psi_x - \psi_y)]\} \quad \text{(S24)}$$

which includes n squared terms and $\frac{n(n-1)}{2}$ cross terms. The squared and cross terms in Eq. (S24) are each simplified by order reduction, allowing for an intuitive distinction between high-frequency and low-frequency components. Then, $P_{\mathrm{Sp}}(t)$ can be expressed as:

$$\begin{aligned} P_{\mathrm{Sp}}(t) &= \epsilon^2 \mathrm{LPF}\left\{\overrightarrow{E_{\mathrm{Sp}}}(t)^2\right\} \\ &= \epsilon^2 \left\{ \sum_{x=1}^{n} \frac{1}{2}\xi_x{}^2 + \sum_{x\neq y}^{\frac{n(n-1)}{2}} \xi_x \xi_y \cos\left[(\Omega_x{}' - \Omega_y{}')t - (\psi_x - \psi_y)\right] \right\} \end{aligned} \tag{S25}$$

where LPF{·} represents the operator for low-pass filtering.

Observing Eq. (S25), it can be found that the statistical characteristics of $P_{\mathrm{Sp}}(t)$ essentially depend on statistical properties of ξ_x that have been expressed by Eq. (S21). Let $a_x = \Sigma_{k=1}^{N_{\mathrm{P}}} F_{kx} \cos(\Phi_{kx}{}')$ and $b_x = \Sigma_{k=1}^{N_{\mathrm{P}}} F_{kx} \sin(\Phi_{kx}{}')$ to rewrite Eq. (S21) as $\xi_x{}^2 = a_x{}^2 + b_x{}^2$ for simplicity here. According to the central limit theorem, as N_{P} is a large number, both a_x and b_x follow the normal distribution. Therefore, the mathematical expectation and variance of a_x can be obtained as:

$$\overline{a_x} = N_{\mathrm{P}} \overline{F_{kx} \cos(\Phi_{kx}{}')} = 0 \tag{S26}$$

$$\mathrm{D}\{a_x\} = N_{\mathrm{P}} \mathrm{D}\{F_{kx} \cos(\Phi_{kx}{}')\} = N_{\mathrm{P}} \overline{F_{kx}{}^2}\, \overline{\cos^2(\Phi_{kx}{}')} = \frac{1}{2} N_{\mathrm{P}} \overline{F_{kx}{}^2} \tag{S27}$$

where D{·} represents the operator for evaluating the variance. Further, the statistical properties of $a_x{}^2$ can be obtained as follows:

$$\overline{a_x{}^2} = \mathrm{D}\{a_x\} \tag{S28}$$

$$\mathrm{D}\{a_x{}^2\} = \overline{a_x{}^4} - \overline{a_x{}^2}^2 = 3\mathrm{D}^2\{a_x\} - \mathrm{D}^2\{a_x\} = 2\mathrm{D}^2\{a_x\} \tag{S29}$$

As it can be derived that $\overline{b_x{}^2} = \overline{a_x{}^2}$ and $\mathrm{D}\{b_x{}^2\} = \mathrm{D}\{a_x{}^2\}$, the statistical properties of $\xi_x{}^2$ can be obtained as follows:

$$\overline{\xi_x{}^2} = \overline{a_x{}^2} + \overline{b_x{}^2} = 2\mathrm{D}\{a_x\} \tag{S30}$$

$$\mathrm{D}\{\xi_x{}^2\} = \mathrm{D}\{a_x{}^2\} + \mathrm{D}\{b_x{}^2\} + 2\mathrm{Cov}\{a_x{}^2, b_x{}^2\} = 4\mathrm{D}^2\{a_x\} \tag{S31}$$

where $\mathrm{Cov}\{a_x{}^2, b_x{}^2\}$ means the covariance of $a_x{}^2$ and $b_x{}^2$, equal to zero. Moreover, we can obtain:

$$\overline{\xi_x{}^4} = \mathrm{D}\{\xi_x{}^2\} + \overline{\xi_x{}^2}^2 = 2\overline{\xi_x{}^2}^2 = 8\mathrm{D}^2\{a_x\} \tag{S32}$$

Based on Eqs. (S25)-(S32), the mathematical expectation of $P_{\mathrm{Sp}}(t)$ can be easily obtained as:

$$\overline{P_{\mathrm{Sp}}(t)} = \epsilon^2 \sum_{x=1}^{n} \frac{1}{2} \overline{\xi_x{}^2} = n\epsilon^2 \mathrm{D}\{a_x\} \tag{S33}$$

And the mathematical expectations of $P_{\mathrm{Sp}}(t)^2$ is:

$$\begin{aligned} \overline{P_{\mathrm{Sp}}(t)^2} &= \epsilon^4 \sum_{x=1}^{n} \frac{1}{4} \overline{\xi_x{}^4} + \epsilon^4 \sum_{x\neq y}^{\frac{n(n-1)}{2}} \overline{\frac{1}{2}\xi_x{}^2 \xi_y{}^2 + \xi_x{}^2 \xi_y{}^2 \cos^2\left[(\Omega_x{}' - \Omega_y{}')t - (\psi_x - \psi_y)\right]} \\ &= \epsilon^4 \sum_{x=1}^{n} \frac{1}{4} \overline{\xi_x{}^4} + \epsilon^4 \sum_{x\neq y}^{\frac{n(n-1)}{2}} \overline{\xi_x{}^2}\, \overline{\xi_y{}^2} = 2n^2 \epsilon^4 \mathrm{D}^2\{a_x\} \end{aligned} \tag{S34}$$

Thus, the variance of $P_{\mathrm{Sp}}(t)$ can be readily derived as:

$$\mathrm{D}\{P_{\mathrm{Sp}}(t)\} = \overline{P_{\mathrm{Sp}}(t)^2} - \overline{P_{\mathrm{Sp}}(t)}^2 = n^2\epsilon^4\mathrm{D}^2\{a_x\} \tag{S35}$$

Finally, the SNR of $P_{\mathrm{Sp}}(t)$, denoted as SNR_{Sp}, is obtained as the ratio of the mathematical expectation of $P_{\mathrm{Sp}}(t)$ to its standard deviation (STD):

$$SNR_{\mathrm{Sp}} = \frac{\overline{P_{\mathrm{Sp}}(t)}}{\sqrt{\mathrm{D}\{P_{\mathrm{Sp}}(t)\}}} = 1 \tag{S36}$$

which agrees well with the value predicted in seminal work[1].

2. Updating the SNR to be B_{m} dependent

Equation (S36) provides a deeper understanding of the spectral characteristics of $P_{\mathrm{Sp}}(t)$, as illustrated in **Fig. 2d** of the main text. Specifically, it shows that the contributions from the direct-current (DC) component (representing the signal) and the alternating-current (AC) component (representing the noise) are equal in power. This perspective allows for an intuitive analysis of how the practical measurement bandwidth B_{m} affects the stochastic behavior of $P_{\mathrm{Sp}}(t)$.

Assuming the AC spectral density follows a Lorentzian profile with bandwidth B_{Sp}, we express it as:

$$L_{\mathrm{or1}}(f) = \frac{a_{Lor1}\left(2B_{\mathrm{Sp}}\right)^2}{\left(2B_{\mathrm{Sp}}\right)^2 + 4f^2}, \qquad f \geq 0 \tag{S37}$$

where f is the frequency variable and a_{Lor1} is the peak value at the center frequency. The noise variance – corresponding to the integrated AC component – can be computed as:

$$\mathrm{D}\{P_{\mathrm{Sp}}(t)\} = \int_0^{B_{\mathrm{m}}} L_{\mathrm{or1}}(f)\mathrm{d}f = \frac{a_{\mathrm{Lor1}}B_{\mathrm{Sp}}}{2}\arctan\left(\frac{B_{\mathrm{m}}}{B_{\mathrm{Sp}}}\right) \tag{S38}$$

Eq. (S38) indicates that B_m defines the effective noise bandwidth. When $B_{\mathrm{m}} > B_{\mathrm{Sp}}$, almost all AC components are captured, whereas if $B_{\mathrm{m}} < B_{\mathrm{Sp}}$, some AC components are truncated, resulting in a reduced noise variance.

According to Eq. (S36), the DC signal component $\overline{P_{\mathrm{Sp}}(t)}$ remains constant (independent of B_{m}) and equals to the total contribution of the full-bandwidth AC component ($B_{\mathrm{m}} \gg B_{\mathrm{Sp}}$), that is, $\sqrt{\pi a_{\mathrm{Lor1}} B_{\mathrm{Sp}}/4}$. Thus, the effective B_{m}-dependent SNR of $P_{\mathrm{Sp}}(t)$ is given by:

$$SNR_{\mathrm{Sp}} = \sqrt{\frac{\pi}{2\arctan\left(\frac{B_{\mathrm{m}}}{B_{\mathrm{Sp}}}\right)}} \tag{S39}$$

As B_{m} increases beyond B_{Sp}, the SNR approaches and eventually saturates at the value of 1.

Supplementary Note S3. Modelling the SNR for FFT-based coherent detection

This note presents the analysis and mathematical derivation of the SNR model for FFT-based coherent detection in the main text. We start by modelling the acquired beating signal between the Stokes SpBS and the lower OLO sideband, based on their respective optical fields reaching the input of C2 (**Fig. 3a** in main text):

$$\text{Stokes SpBS: } \overrightarrow{E_{\mathrm{Sp1}}}(t) = \hat{x}E_{\mathrm{Sp1}}(t)\mathrm{e}^{\mathrm{j}\varphi_{\mathrm{Sp1}}^{x}(t)}\mathrm{e}^{\mathrm{j}2\pi f_{\mathrm{Sp1}}t} \quad \text{(S40)}$$

$$\text{lower OLO sideband: } \overrightarrow{E_{Lo1}}(t) = \hat{x}E_{\mathrm{Lo1}}\mathrm{e}^{\mathrm{j}2\pi f_{\mathrm{Lo1}}t} \quad \text{(S41)}$$

where $\hat{x}$ stands for the polarization direction; $\varphi_{\mathrm{Sp1}}^{x}(t)$ is the random phase difference in $\hat{x}$ polarization direction between the SpBS signal and OLO light reaching the input of C2; E_{Sp1} and E_{Lo1} are amplitudes of the Stokes wave and OLO; j is an imaginary unit; f_{Sp1} and f_{Lo1} represent the carrier frequencies of the Stokes wave and OLO.

After passing through the 2 × 2 optical coupler and the subsequent balanced photodetector (BPD), the resulting beating signal $s(t)$ is derived as:

$$s(t) = 2\mathcal{R}_{\mathrm{p}}\sqrt{P_{\mathrm{Lo}}P_{\mathrm{Sp}}(t)}\cos[2\pi f_{\mathrm{c}}t + \Phi_1(t)] \quad \text{(S42)}$$

where $\mathcal{R}_{\mathrm{p}} \approx 0.95$ A/W is the responsivity of the photodiode; $P_{\mathrm{Sp}}(t) \propto E_{\mathrm{Sp1}}(t)^2$ and $P_{\mathrm{Lo}} \propto {E_{\mathrm{Lo1}}}^2$ are the optical power of the SpBS light and lower OLO sideband, respectively, where $P_{\mathrm{Sp}}(t)$ fluctuates randomly following the statistical property shown by Eq. (S39) in **Note S2**; $f_{\mathrm{c}} = |f_{\mathrm{Sp1}} - f_{\mathrm{Lo1}}|$ is the beating frequency of SpBS and OLO; $\Phi_1(t) = \varphi_{\mathrm{Sp1}}^{x}(t) - \pi/2$ is the random phase difference between the SpBS signal and OLO reaching the BPD.

Note that, during the photoelectric conversion process, the photo-detection noise $e(t)$ – characterized as zero-mean Gaussian white noise – is inevitably superimposed on the signal $s(t)$. Typically, $e(t)$ consists of thermal noise and shot noise (mainly contributed by the OLO), and the total noise power spectral density (PSD) ${\sigma_e}^2$ is given by[7,8]:

$${\sigma_e}^2 \approx {\sigma_T}^2 + 2q\mathcal{R}_{\mathrm{p}}P_{\mathrm{Lo}} \quad \text{(S43)}$$

where ${\sigma_T}^2$ is the PSD of thermal noise (assumed to be flat), and $q = 1.6 \times 10^{-19}$ C is the electron charge. Thereby the total noise variance is expressed as ${\sigma_e}^2 B_e$, where B_e is the noise bandwidth.

Taking into account the abovementioned detection noise, the time-domain response of the beating signal, designated here as $r(t)$, is the linear superposition of the signal $s(t)$ and photo-detection noise $e(t)$. With a sampling rate of f_{s} and a total sampling points number of N_{F}, its discrete-time expression with the variable n is:

$$r(n) = s(n) + e(n) \quad \text{(S44)}$$

where n=1, 2, 3, …, N_{F}. The key operation of FFT-based coherent detection is to perform a discrete-domain fast Fourier transform (FFT) on $r(n)$, obtaining a normalized PSD, which is then used to analyze the SNR performance at the peak of the Brillouin spectrum. Performing the discrete FFT on $r(n)$ results in the discrete frequency-domain expression with the variable k:

$$R(k) = S(k) + E(k) \quad \text{(S45)}$$

where k=1, 2, 3, …, N_{F}. The PSD of $R(k)$, normalized by the points number N_{F}, is given by:

$$\frac{|R(k)|^2}{N_{\mathrm{F}}} = \frac{|S(k) + E(k)|^2}{N_{\mathrm{F}}} = \frac{|S(k)|^2}{N_{\mathrm{F}}} + \frac{|E(k)|^2}{N_{\mathrm{F}}} + \frac{2S_{\mathrm{Re}}(k)E_{\mathrm{Re}}(k) + 2S_{\mathrm{Im}}(k)E_{\mathrm{Im}}(k)}{N_{\mathrm{F}}} \quad \text{(S46)}$$

where $|\cdot|$ represents the modulus of a complex expression; the subscripts Re and Im respectively denote the real and imaginary parts in the complex domain. Eq. (S46) indicates that the normalized PSD of $r(n)$ consists of three components: 1) the normalized PSD of $s(n)$, represented by the first

term on the right-hand side of Eq. (S46), 2) the normalized PSD of $e(n)$, represented by the second term on the right-hand side of Eq. (S46), and 3) the cross term between the normalized PSD of $s(n)$ and $e(n)$, represented by the third term on the right-hand side of Eq. (S46). In the following, the statistical properties of each component are sequentially analyzed.

1) The normalized PSD of $s(n)$. Given the complexity of directly analyzing the PSD of $s(n)$, here an indirect analytical approach is strategically carried out. We first ignore the random-phase term in $s(n)$, thus simplifying it to a single-frequency cosine signal $s_{\mathrm{sim}}(n)$ as:

$$s_{\mathrm{sim}}(n) = A\cos\left[\frac{2\pi f_{\mathrm{c}}(n-1)}{f_{\mathrm{s}}}\right] = A\cos\left[\frac{2\pi(n-1)}{a}\right] \tag{S47}$$

where $A = 2\mathcal{R}_{\mathrm{p}}\sqrt{P_{\mathrm{Lo}}P_{\mathrm{Sp}}(t)}$; $f_{\mathrm{s}} = af_{\mathrm{c}}$, $N_{\mathrm{F}} = b\frac{f_{\mathrm{s}}}{f_{\mathrm{c}}} = ab$, a and b being positive integers, to avoid spectral leakage. This operation ensures that all the signal energy is concentrated at one single frequency point, whose normalized PSD can be conveniently derived, being. Using this single-point PSD, the actual peak value of the normalized PSD of $s(n)$, is later on derived based on the principle of energy conservation.

The following steps focus on obtaining the single-frequency normalized PSD. First, the discrete fast Fourier transformation of $s_{\mathrm{sim}}(n)$ is:

$$S_{\mathrm{sim}}(k) = \sum_{n=1}^{N_{\mathrm{F}}} s_{\mathrm{sim}}(n)\mathrm{e}^{-j\frac{2\pi}{N_{\mathrm{F}}}(n-1)(k-1)} \tag{S48}$$

The center frequency f_{c} corresponds to the k_{c}-th ($k_{\mathrm{c}} \neq 1$) point in the frequency domain, where $k_{\mathrm{c}} = f_{\mathrm{c}}/(f_{\mathrm{s}}/N_{\mathrm{F}}) + 1 = b + 1$, as shown in **Fig. S2**. We can expand $S_{\mathrm{sim}}(k)$ at $k = k_{\mathrm{c}}$ as:

$$\begin{aligned} S_{\mathrm{sim}}(k_{\mathrm{c}}) &= \sum_{n=1}^{N_{\mathrm{F}}} A\cos\left[\frac{2\pi(n-1)}{a}\right]\cos\left[\frac{2\pi(n-1)}{a}\right] \\ &+\mathrm{j}\sum_{n=1}^{N_{\mathrm{F}}} -A\cos\left[\frac{2\pi(n-1)}{a}\right]\sin\left[\frac{2\pi(n-1)}{a}\right] \\ &= N_{\mathrm{F}}\frac{A}{2} + \sum_{n=1}^{N_{\mathrm{F}}}\frac{A}{2}\cos\left(\frac{n-1}{a}4\pi\right) + \mathrm{j}\sum_{n=1}^{N_{\mathrm{F}}} -\frac{A}{2}\sin\left(\frac{n-1}{a}4\pi\right) \end{aligned} \tag{S49}$$

As the last two terms equal to 0, the normalized PSD of $s_{\mathrm{sim}}(n)$ at $k = k_{\mathrm{c}}$ is expressed as:

$$\frac{|S_{\mathrm{sim}}(k_{\mathrm{c}})|^2}{N_F} = \frac{1}{4}N_{\mathrm{F}}A^2 = N_{\mathrm{F}}{\mathcal{R}_{\mathrm{p}}}^2 P_{\mathrm{Lo}}P_{\mathrm{Sp}}(t) \tag{S50}$$

We now proceed to derive the corresponding peak expression for the PSD of the actual beating signal $s(n)$. In contrast to the single-frequency case of $s_{\mathrm{sim}}(n)$, the spectrum of $s(n)$ exhibits a Lorentzian shape with a FWHM of B_{Sp}, as illustrated in **Fig. S2**, which is denoted as $L_{\mathrm{or2}}(f)$ and can be expressed as:

$$L_{\mathrm{or2}}(f) = \frac{|S(k_{\mathrm{c}})|^2}{N_{\mathrm{F}}}\frac{{B_{\mathrm{Sp}}}^2}{{B_{\mathrm{Sp}}}^2 + 4(f - f_{\mathrm{c}})^2} \tag{S51}$$

where f is the frequency variable; f_c is the center frequency; $\frac{|S(k_c)|^2}{N_F}$ is the peak value at the center frequency of the normalized PSD. According to the law of energy conservation, the area under the single-frequency case is equal to that of the Lorentzian-shaped case, and thus we have:

$$\frac{|S_{\text{sim}}(k_c)|^2}{N_F}\delta f = \int_{-\infty}^{+\infty} L_{\text{or2}}(f)\mathrm{d}f = \frac{\pi}{2}\frac{|S(k_c)|^2}{N_F}B_{\text{Sp}} \tag{S52}$$

where δf is the spectral resolution. Then the peak of the normalized PSD of $S(k)$ can be obtained:

$$\frac{|S(k_c)|^2}{N_F} = \frac{2N_F\delta f}{\pi B_{\text{Sp}}}\mathcal{R}_p{}^2 P_{\text{Lo}}P_{\text{Sp}}(t) = \frac{2f_s}{\pi B_{\text{Sp}}}\mathcal{R}_p{}^2 P_{\text{Lo}}P_{\text{Sp}}(t) \tag{S53}$$

whose mean value then can be readily derived as:

$$\overline{\frac{|S(k_c)|^2}{N_F}} = \frac{2f_s}{\pi B_{\text{Sp}}}\mathcal{R}_p{}^2 P_{\text{Lo}}\overline{P_{\text{Sp}}} \tag{S54}$$

And its variance can be derived as:

$$\mathrm{D}\left\{\frac{|S(k_c)|^2}{N_F}\right\} = \frac{4f_s{}^2}{\pi^2 B_{\text{Sp}}{}^2}\mathcal{R}_p{}^4 P_{\text{Lo}}{}^2\mathrm{D}\{P_{\text{Sp}}\} = \frac{4f_s{}^2}{\pi^2 B_{\text{Sp}}{}^2}\mathcal{R}_p{}^4 P_{\text{Lo}}{}^2\frac{\overline{P_{\text{Sp}}}^2}{SNR_{\text{Sp}}{}^2} \tag{S55}$$

The average power of SpBS signal in the amplified spontaneous regime can be expressed separately for the Stokes and anti-Stokes components as follows[1,2,9]:

$$\overline{P_{\text{Sp}}^{\text{S}}} = \beta_1^{\text{S}}\left(\mathrm{e}^{\beta_2 P_p} - 1\right) \tag{S56}$$

$$\overline{P_{\text{Sp}}^{\text{AS}}} = \beta_1^{\text{AS}}\left(1 - \mathrm{e}^{-\beta_2 P_p}\right) \tag{S57}$$

where P_p is the pump power; β_1^{S}, β_1^{AS} and β_2 are constant coefficients and can be expressed as:

$$\beta_1^{\text{S}} = \frac{8\pi\hbar\omega_{\text{S}}(\bar{n}+1)}{ncA_{\text{eff}}\Gamma} \tag{S58}$$

$$\beta_1^{\text{AS}} = \frac{8\pi\hbar\omega_{\text{AS}}\bar{n}}{ncA_{\text{eff}}\Gamma} \tag{S59}$$

$$\beta_2 = \frac{g_0 L}{A_{\text{eff}}} \tag{S60}$$

where $\hbar$ is the reduced Planck constant; ω_{S} and ω_{AS} are the angular frequencies of the Stokes and anti-Stokes light, respectively; $\bar{n}$ is the mean number of phonons per mode of the acoustic field; A_{eff} is the effective mode field area of the fiber; Γ is the phonon (intensity) decay rate; g_0 is the Brillouin gain factor; L is the fiber length. Unless explicitly stated otherwise, $\overline{P_{\text{Sp}}}$ in this Note refers to the average power of the Stokes component, i.e., $\overline{P_{\text{Sp}}} = \overline{P_{\text{Sp}}^{\text{S}}}$.

2) The normalized PSD of $e(n)$. The analysis starts with the discrete fast Fourier transformation of $e(n)$ as:

$$E(k) = \sum_{n=1}^{N_F} e(n)\mathrm{e}^{-j\frac{2\pi}{N_F}(n-1)(k-1)} \tag{S61}$$

And the normalized PSD of $e(n)$ at $k = k_c$ ($k_c \neq 1$) is expressed as:

$$\frac{|E(k_c)|^2}{N_F} = \frac{E_{\text{Re}}(k_c)^2 + E_{\text{Im}}(k_c)^2}{N_F} \tag{S62}$$

where $E_{\mathrm{Re}}(k_{\mathrm{c}})$ and $E_{\mathrm{Im}}(k_{\mathrm{c}})$ stand for the real and imaginary parts of $E(k)$ when $k = k_{\mathrm{c}}$, which can be respectively expressed as:

$$E_{\mathrm{Re}}(k_{\mathrm{c}}) = \sum_{n=1}^{N_{\mathrm{F}}} e(n)\cos\left(\frac{n-1}{N_{\mathrm{F}}}K\pi\right) \tag{S63}$$

$$E_{\mathrm{Im}}(k_{\mathrm{c}}) = \sum_{n=1}^{N_{\mathrm{F}}} -e(n)\sin\left(\frac{n-1}{N_{\mathrm{F}}}K\pi\right) \tag{S64}$$

where $K = 2(k_{\mathrm{c}} + 1)$ is a positive even number. Eq. (S62) indicates that the statistical properties of $|E(k_{\mathrm{c}})|^2$ can be derived by separately characterizing the statistical properties of $E_{\mathrm{Re}}(k_{\mathrm{c}})^2$ and $E_{\mathrm{Im}}(k_{\mathrm{c}})^2$, both indeed sharing same statistical characteristics according to Eqs. (S63) and (S64). Thereby the analysis below only focuses on the real part $E_{\mathrm{Re}}(k_{\mathrm{c}})^2$.

Based on Eq. (S63), we first present the expression for $E_{\mathrm{Re}}(k_{\mathrm{c}})^2$, which contains N_{F} squared terms and $\frac{N_{\mathrm{F}}(N_{\mathrm{F}}-1)}{2}$ cross terms as:

$$E_{\mathrm{Re}}(k_{\mathrm{c}})^2 = \sum_{n=1}^{N_{\mathrm{F}}} e(n)^2\cos^2\left(\frac{n-1}{N_{\mathrm{F}}}K\pi\right) + \sum_{n\neq m}^{\frac{N_{\mathrm{F}}(N_{\mathrm{F}}-1)}{2}} 2e(n)\cos\left(\frac{n-1}{N_{\mathrm{F}}}K\pi\right)e(m)\cos\left(\frac{m-1}{N_{\mathrm{F}}}K\pi\right) \tag{S65}$$

Since the cross terms in Eq. (S65) have a mean value of zero, the expected value of $E_{\mathrm{Re}}(k_{\mathrm{c}})^2$ is given by:

$$\overline{E_{\mathrm{Re}}(k_{\mathrm{c}})^2} = \sum_{n=1}^{N_{\mathrm{F}}} \overline{e(n)^2}\cos^2\left(\frac{n-1}{N_{\mathrm{F}}}K\pi\right) = \frac{1}{2}N_{\mathrm{F}}{\sigma_e}^2 B_e \tag{S66}$$

The variance of $E_{\mathrm{Re}}(k_{\mathrm{c}})^2$ is then calculated as:

$$\begin{aligned}
\mathrm{D}\{E_{\mathrm{Re}}(k_{\mathrm{c}})^2\} &= \mathrm{D}\left\{\sum_{n=1}^{N_{\mathrm{F}}} e(n)^2\cos^2\left(\frac{n-1}{N_{\mathrm{F}}}K\pi\right)\right\} \\
&+\mathrm{D}\left\{\sum_{n\neq m}^{\frac{N_{\mathrm{F}}(N_{\mathrm{F}}-1)}{2}} 2e(n)\cos\left(\frac{n-1}{N_{\mathrm{F}}}K\pi\right)e(m)\cos\left(\frac{m-1}{N_{\mathrm{F}}}K\pi\right)\right\} \\
&+2\mathrm{Cov}\left\{\sum_{n=1}^{N_{\mathrm{F}}} e(n)^2\cos^2\left(\frac{n-1}{N_{\mathrm{F}}}K\pi\right),\ \sum_{n\neq m}^{\frac{N_{\mathrm{F}}(N_{\mathrm{F}}-1)}{2}} 2e(n)\cos\left(\frac{n-1}{N_{\mathrm{F}}}K\pi\right)e(m)\cos\left(\frac{m-1}{N_{\mathrm{F}}}K\pi\right)\right\} \\
&= \sum_{n=1}^{N_{\mathrm{F}}}\cos^4\left(\frac{n-1}{N_{\mathrm{F}}}K\pi\right)\mathrm{D}\{e(n)^2\} + \sum_{n\neq m}^{\frac{N_{\mathrm{F}}(N_{\mathrm{F}}-1)}{2}} 4\cos^2\left(\frac{n-1}{N_{\mathrm{F}}}K\pi\right)\cos^2\left(\frac{m-1}{N_{\mathrm{F}}}K\pi\right)\mathrm{D}\{e(n)e(m)\} \\
&= \left(\frac{1}{2}{N_{\mathrm{F}}}^2+\frac{1}{4}N_{\mathrm{F}}\right)({\sigma_e}^2B_e)^2
\end{aligned} \tag{S67}$$

Then, the mathematical expectation and variance of the normalized PSD of $E(k_{\mathrm{c}})$ can be respectively obtained as:

$$\frac{\overline{|E(k_{\mathrm{c}})|^2}}{N_{\mathrm{F}}} = \frac{\overline{E_{\mathrm{Re}}(k_{\mathrm{c}})^2}}{N_{\mathrm{F}}} + \frac{\overline{E_{\mathrm{Im}}(k_{\mathrm{c}})^2}}{N_{\mathrm{F}}} = {\sigma_e}^2B_e \tag{S68}$$

$$\mathrm{D}\left\{\frac{|E(k_{\mathrm{c}})|^2}{N_{\mathrm{F}}}\right\} = \mathrm{D}\left\{\frac{E_{\mathrm{Re}}(k_{\mathrm{c}})^2}{N_{\mathrm{F}}}\right\} + \mathrm{D}\left\{\frac{E_{\mathrm{Im}}(k_{\mathrm{c}})^2}{N_{\mathrm{F}}}\right\} = \left(1+\frac{1}{2N_{\mathrm{F}}}\right)({\sigma_e}^2B_e)^2 \approx ({\sigma_e}^2B_e)^2 \tag{S69}$$

It should be noted that the above results represent the equivalent PSD, as shown by red square in **Fig. S3**, which spreads the original energy distribution to sampling bandwidth of $f_{\mathrm{s}}/2$. So, the actual PSD under bandwidth of B_e, as shown by green square in **Fig. S3**, should be further derived, whose expected value and variance are given by:

$$\frac{\overline{|E(k_{\mathrm{c}})|^2}}{N_{\mathrm{F}}} = {\sigma_e}^2B_e\frac{f_{\mathrm{s}}}{2B_e} = {\sigma_e}^2\frac{f_{\mathrm{s}}}{2} \tag{S70}$$

$$\mathrm{D}\left\{\frac{|E(k_{\mathrm{c}})|^2}{N_{\mathrm{F}}}\right\} \approx \left({\sigma_e}^2B_e\frac{f_{\mathrm{s}}}{2B_e}\right)^2 = \left({\sigma_e}^2\frac{f_{\mathrm{s}}}{2}\right)^2 \tag{S71}$$

3) The cross term. The mathematical expectation of the cross term between $s(n)$ and $e(n)$, represented by the third term on the right-hand side of Eq. (S46), is given by:

$$\overline{\frac{2}{N_{\mathrm{F}}}[S_{\mathrm{Re}}(k_{\mathrm{c}})E_{\mathrm{Re}}(k_{\mathrm{c}})+S_{\mathrm{Im}}(k_{\mathrm{c}})E_{\mathrm{Im}}(k_{\mathrm{c}})]} = \frac{2}{N_{\mathrm{F}}}\left[\overline{S_{\mathrm{Re}}(k_{\mathrm{c}})}\,\overline{E_{\mathrm{Re}}(k_{\mathrm{c}})}+\overline{S_{\mathrm{Im}}(k_{\mathrm{c}})}\,\overline{E_{\mathrm{Im}}(k_{\mathrm{c}})}\right] = 0 \tag{S72}$$

Its variance is further derived as:

$$\begin{aligned}
\mathrm{D}\left\{\frac{2S_{\mathrm{Re}}(k_{\mathrm{c}})E_{\mathrm{Re}}(k_{\mathrm{c}})+2S_{\mathrm{Im}}(k_{\mathrm{c}})E_{\mathrm{Im}}(k_{\mathrm{c}})}{N_{\mathrm{F}}}\right\} &= \frac{4}{{N_{\mathrm{F}}}^2}\overline{[S_{\mathrm{Re}}(k_{\mathrm{c}})E_{\mathrm{Re}}(k_{\mathrm{c}})+S_{\mathrm{Im}}(k_{\mathrm{c}})E_{\mathrm{Im}}(k_{\mathrm{c}})]^2} \\
= \frac{4}{{N_{\mathrm{F}}}^2}\left[\overline{S_{\mathrm{Re}}(k_{\mathrm{c}})^2}\,\overline{E_{\mathrm{Re}}(k_{\mathrm{c}})^2}+\overline{S_{\mathrm{Im}}(k_{\mathrm{c}})^2}\,\overline{E_{\mathrm{Im}}(k_{\mathrm{c}})^2}+\overline{2S_{\mathrm{Re}}(k_{\mathrm{c}})E_{\mathrm{Re}}(k_{\mathrm{c}})S_{\mathrm{Im}}(k_{\mathrm{c}})E_{\mathrm{Im}}(k_{\mathrm{c}})}\right]
\end{aligned} \tag{S73}$$

Given that $\overline{E_{\mathrm{Re}}(k_{\mathrm{c}})E_{\mathrm{Im}}(k_{\mathrm{c}})} = 0$, Eq. (S73) can be simplified as:

$$\begin{aligned}\mathrm{D}\left\{\frac{2S_{\mathrm{Re}}(k_{\mathrm{c}})E_{\mathrm{Re}}(k_{\mathrm{c}})+2S_{\mathrm{Im}}(k_{\mathrm{c}})E_{\mathrm{Im}}(k_{\mathrm{c}})}{N_{\mathrm{F}}}\right\} &= 4\left[\frac{\overline{S_{\mathrm{Re}}(k_{\mathrm{c}})^2}}{N_{\mathrm{F}}}\frac{\overline{E_{\mathrm{Re}}(k_{\mathrm{c}})^2}}{N_{\mathrm{F}}}+\frac{\overline{S_{\mathrm{Im}}(k_{\mathrm{c}})^2}}{N_{\mathrm{F}}}\frac{\overline{E_{\mathrm{Im}}(k_{\mathrm{c}})^2}}{N_{\mathrm{F}}}\right] \\ &= 2\frac{\overline{|S(k_{\mathrm{c}})|^2}}{N_{\mathrm{F}}}\frac{\overline{|E(k_{\mathrm{c}})|^2}}{N_{\mathrm{F}}} = \frac{2f_{\mathrm{s}}{}^2}{\pi B_{\mathrm{Sp}}}\mathcal{R}_{\mathrm{p}}{}^2 P_{\mathrm{Lo}}\overline{P_{\mathrm{Sp}}}\sigma_e{}^2\end{aligned} \tag{S74}$$

All above-derived statistic parameters – expectation values and variances on the peak of normalized PSD corresponding to the three terms in Eq. (S46) – are summarized in Table S1 to eventually model the SNR of FFT-based coherent detection. As shown in the table, the actual signal term is given by Eq. (S54), while the constant noise bias given by Eq. (S70) remains trivial and can be practically eliminated in measurements by subtracting the PSD obtained in the absence of the pump light.

Consequently, the SNR of the response acquired through FFT-based coherent detection can be expressed as:

$$\begin{aligned}\mathrm{SNR}\{r_{Co}^{FFT}\} &= \frac{\dfrac{\overline{|S(k_{\mathrm{c}})|^2}}{N_{\mathrm{F}}}}{\sqrt{\mathrm{D}\left\{\dfrac{|S(k_{\mathrm{c}})|^2}{N_{\mathrm{F}}}\right\}+\mathrm{D}\left\{\dfrac{2S_{\mathrm{Re}}(k_{\mathrm{c}})E_{\mathrm{Re}}(k_{\mathrm{c}})+2S_{\mathrm{Im}}(k_{\mathrm{c}})E_{\mathrm{Im}}(k_{\mathrm{c}})}{N_{\mathrm{F}}}\right\}+\mathrm{D}\left\{\dfrac{|E(k_{\mathrm{c}})|^2}{N_{\mathrm{F}}}\right\}}} \\ &= \frac{\dfrac{2f_{\mathrm{s}}}{\pi B_{\mathrm{Sp}}}\mathcal{R}_{\mathrm{p}}{}^2 P_{\mathrm{Lo}}\overline{P_{\mathrm{Sp}}}}{\sqrt{\dfrac{4f_{\mathrm{s}}{}^2}{\pi^2 B_{\mathrm{Sp}}{}^2}\mathcal{R}_{\mathrm{p}}{}^4 P_{\mathrm{Lo}}{}^2\dfrac{\overline{P_{\mathrm{Sp}}}^2}{SNR_{\mathrm{Sp}}{}^2}+\dfrac{2f_{\mathrm{s}}{}^2}{\pi B_{\mathrm{Sp}}}\mathcal{R}_{\mathrm{p}}{}^2 P_{\mathrm{Lo}}\overline{P_{\mathrm{Sp}}}\sigma_e{}^2+\left(\sigma_e{}^2\dfrac{f_{\mathrm{s}}}{2}\right)^2}} \\ &= \frac{\mathcal{R}_{\mathrm{p}}{}^2 P_{\mathrm{Lo}}\overline{P_{\mathrm{Sp}}}}{\sqrt{\dfrac{1}{SNR_{\mathrm{Sp}}{}^2}\mathcal{R}_{\mathrm{p}}{}^4 P_{\mathrm{Lo}}{}^2\overline{P_{\mathrm{Sp}}}^2+\dfrac{\pi B_{\mathrm{Sp}}}{2}\mathcal{R}_{\mathrm{p}}{}^2 P_{\mathrm{Lo}}\overline{P_{\mathrm{Sp}}}\sigma_e{}^2+\left(\dfrac{\pi B_{\mathrm{Sp}}}{4}\right)^2\sigma_e{}^4}}\end{aligned} \tag{S75}$$

Note that, The FFT-based coherent detection method inherently yields a constant $SNR_{\mathrm{Sp}} = 1$, corresponding to the case $B_{\mathrm{m}} \geq B_{\mathrm{Sp}}$ in Eq. (S39) as illustrated in **Fig. S4a**. This is because, as shown in **Fig. S4b**, FFT-based detection effectively samples a single frequency point at the center of the Brillouin spectrum – functionally equivalent to applying a narrow bandpass filter (BPF) with bandwidth equal to the FFT spectral resolution δf. As a result, both the spectral amplitude at zero frequency and the total envelope spectral area are reduced by a factor of $\left(\delta f/B_{\mathrm{Sp}}\right)^2$ compared to the full-band case in **Fig. S4a**, thereby maintaining SNR_{Sp}=1.

Consequently, the FFT-based detection scheme inherently represents the $SNR_{\mathrm{Sp}} = 1$ regime and does not capture the bandwidth-dependent behavior of SNR_{Sp}. Although a B_{m}-dependent SNR_{Sp} could be artificially introduced by applying a digital low-pass filter (LPF) with a cutoff bandwidth smaller than δf, we advocate for a more physically grounded approach: the envelope detector-based detection scheme. This alternative, described in detail in the **Methods** section of the main text, enables a systematic investigation of the relationship between B_{m} and SNR_{Sp}.

The foregoing discussion in this note corresponds to the theoretical analysis of coherent detection, whereas direct detection, being more straightforward, has its SNR model directly represented by Eq. (3) in the main text. In **Figs. 3** and **4** of the main text, we present the analytical and experimental results for the Stokes component under coherent and direct detection schemes,

respectively, while the corresponding results for the anti-Stokes component are provided in **Fig. S5**, following a presentation format consistent with that of the main text. As observed in **Fig. S5**, the theoretical predictions (dark solid line) for the anti-Stokes component agree well with the experimental measurements (light circles), corroborating that the anti-Stokes and Stokes signals follow the similar derivation model and share identical stochastic properties. Moreover, the theoretical and experimental results reveal that the coexistence of stimulated and spontaneous processes yields higher power in the Stokes signal compared to the anti-Stokes signal. For brevity and clarity of presentation, only the Stokes results are included in the main text.

Supplementary Note S4. Modelling the SNR of single-pulse BOTDR using polarization-maintaining fiber

In this note, we present a model for the signal-to-noise ratio (SNR) of the distance-domain envelope signal[10] at the Brillouin resonance, as obtained from a frequency-scanning Brillouin optical time-domain reflectometer (BOTDR) implemented with a polarization-maintaining fiber (PMF), as shown in **Fig. 6a** of the main text. Regardless of whether an off-the-shelf envelope detector (ED) or digital post-processing is used, the envelope extraction process can be mathematically described as follows: the beating signal is first passed through a bandpass filter (BPF), squared, and then processed with a low-pass filter LPF{·} to remove high-frequency components.

First, we model the distance-domain photocurrent output from BPF, designated here as $I(z)$, which results from the beating between the SpBS components and the OLO sidebands. Its expression is similar to that in ref. 10, except for the absence of polarization-related terms due to the use of PMF:

$$\begin{aligned} I(z) &= I_s(z) + I_e(z) \\ &= 2\mathcal{R}_\mathrm{p}\left\{\sqrt{P_\mathrm{Lo1}P_\mathrm{Sp1}(z)}\cos[H(z)+\Phi_1(z)] + \sqrt{P_\mathrm{Lo2}P_\mathrm{Sp2}(z)}\cos[H(z)+\Phi_2(z)]\right\} + I_e(z) \end{aligned} \quad (S76)$$

where $I_s(z)$ is the photocurrent of the beating signal output from the BPF; $\mathcal{R}_\mathrm{p} \approx 0.95$ A W^{-1} is the responsivity of the photodiodes; $f_\mathrm{c}(z)$ denotes the frequency difference between the SpBS light and the OLO light; $H(z) = 4\pi f_\mathrm{c}(z) n_\mathrm{eff} z/c$ indicates the carrier phase of the beating signal; $\Phi_1(t)$ (or $\Phi_2(t)$) is the random phase differences between the Stokes (or anti-Stokes) SpBS signal and the lower (or upper) OLO sideband. P_Lo1 and P_Lo2 are the power of the lower and upper OLO sidebands, respectively, with $P_\mathrm{Lo1} = P_\mathrm{Lo2} = P_\mathrm{Lo}/2$. $P_\mathrm{Sp1}(z)$ and $P_\mathrm{Sp2}(z)$ are the power of the Stokes and anti-Stokes SpBS signals, respectively, which fluctuate randomly due to the intrinsic stochastic nature, both with mean value expressed as:

$$\overline{P_\mathrm{Sp1}(z)} = \overline{P_\mathrm{Sp2}(z)} = \frac{\overline{P_\mathrm{Sp}(z)}}{2} = \frac{k_\mathrm{Sp} c P_\mathrm{p} D_\mathrm{p} \mathrm{e}^{-2\alpha z}}{2n_\mathrm{eff}} \quad (S77)$$

where k_Sp is the backscattering coefficient of SpBS, P_p and D_p are the peak power and duration of the incident pump pulse, respectively, n_eff is the effective group index of the propagating mode in the fiber, and α is the fiber attenuation coefficient. $I_e(z)$ in Eq. (S76) is the noise photocurrent at the BPF output, mainly attributed to filtered thermal noise and shot noise. The variance of $I_e(z)$ can be characterized as ${\sigma_e}^2 B_\mathrm{BPF}$, where ${\sigma_e}^2$ is the photo-detection noise PSD as described by Eq. (S43) in **Note S3**, and B_BPF is the BPF bandwidth, approximately equal to the FWHM of the Brillouin spectrum B_Sp.

The output of the BPF then undergoes an envelope extraction process with a LPF bandwidth B_m (generally equal to B_BPF), yielding the power envelope signal:

$$r_\mathrm{Sg}^\mathrm{PM}(z) = \mathrm{LPF}\{I(z)^2\} = \mathrm{LPF}\{I_s(z)^2\} + \mathrm{LPF}\{2I_s(z)I_e(z)\} + \mathrm{LPF}\{I_e(z)^2\} \quad (S78)$$

We then derive the statistical parameters – the expectation value and variance – for each term in Eq. (S78) at every fiber position z, as detailed below.

1) The term of LPF$\{\boldsymbol{I_s(z)^2}\}$. Firstly, squaring $I_s(z)$ in Eq. (S76) gives:

$$I_s(z)^2 = 4\mathcal{R}_\mathrm{p}{}^2 P_\mathrm{Lo1} P_\mathrm{Sp1}(z)\cos^2[H(z)+\Phi_1(z)] + 4\mathcal{R}_\mathrm{p}{}^2 P_\mathrm{Lo2} P_\mathrm{Sp2}(z)\cos^2[H(z)+\Phi_2(z)]$$
$$+8\mathcal{R}_\mathrm{p}{}^2\sqrt{P_\mathrm{Lo1}P_\mathrm{Sp1}(z)P_\mathrm{Lo2}P_\mathrm{Sp2}(z)}\cos[H(z)+\Phi_1(z)]\cos[H(z)+\Phi_2(z)] \tag{S79}$$

After applying the product-to-sum transformation, the terms containing $\cos[2H(z)]$ signify the high-frequency components (typically a few hundred megahertz and beyond B_m), which are filtered out by the low-pass filtering operation, leading to:

$$\mathrm{LPF}\{I_s(z)^2\} = 2\mathcal{R}_\mathrm{p}{}^2 P_\mathrm{Lo1}P_\mathrm{Sp1}(z) + P_\mathrm{Lo2}P_\mathrm{Sp2}(z)$$
$$+4\mathcal{R}_\mathrm{p}{}^2\sqrt{P_\mathrm{Lo1}P_\mathrm{Sp1}(z)P_\mathrm{Lo2}P_\mathrm{Sp2}(z)}\cos[\Delta\Phi(z)] \tag{S80}$$

where $\Delta\Phi(z) = \Phi_1(z) - \Phi_2(z)$. Considering that $\Phi_1(z)$ and $\Phi_2(z)$ follow a uniform distribution over the interval [-π, π], the expectation value of $\cos[\Delta\Phi(z)]$ is considered zero, i.e., $\overline{\cos[\Delta\Phi(z)]} = 0$. This allows us to express the expectation value of $\mathrm{LPF}\{I_s(z)^2\}$, designated as $\mu_{s^2}(z)$, as:

$$\mu_{s^2}(z) = \overline{\mathrm{LPF}\{I_s^\mathrm{BPF}(z)^2\}} = 2\mathcal{R}_\mathrm{p}{}^2\left[P_\mathrm{Lo1}\overline{P_\mathrm{Sp1}(z)} + P_\mathrm{Lo2}\overline{P_\mathrm{Sp2}(z)}\right] = \mathcal{R}_\mathrm{p}{}^2 P_\mathrm{Lo}\overline{P_\mathrm{Sp}(z)} \tag{S81}$$

Then based on Eq. (S39) and the definition $\mathrm{D}\{P_\mathrm{Sp}(z)\} = \overline{P_\mathrm{Sp1}(z)^2} - \overline{P_\mathrm{Sp1}(z)}^2$, the following relations hold:

$$\overline{P_\mathrm{Sp1}(z)^2} = \left(1+\frac{1}{SNR_\mathrm{Sp}{}^2}\right)\overline{P_\mathrm{Sp1}(z)}^2 \tag{S82}$$

$$\overline{P_\mathrm{Sp2}(z)^2} = \left(1+\frac{1}{SNR_\mathrm{Sp}{}^2}\right)\overline{P_\mathrm{Sp2}(z)}^2 \tag{S83}$$

enabling calculating the mean value of $\mathrm{LPF}^2\{I_s(z)^2\}$:

$$\overline{\mathrm{LPF}^2\{I_s(z)^2\}} = 4\mathcal{R}_\mathrm{p}{}^4\left\{P_\mathrm{Lo1}{}^2\overline{P_\mathrm{Sp1}(z)^2} + P_\mathrm{Lo2}{}^2\overline{P_\mathrm{Sp2}(z)^2} + 4P_\mathrm{Lo1}\overline{P_\mathrm{Sp1}(z)}P_\mathrm{Lo2}\overline{P_\mathrm{Sp2}(z)}\right\}$$
$$= 4\mathcal{R}_\mathrm{p}{}^4 P_\mathrm{Lo1}{}^2\left(1+\frac{1}{SNR_\mathrm{Sp}{}^2}\right)\overline{P_\mathrm{Sp1}(z)}^2 + 4\mathcal{R}_\mathrm{p}{}^4 P_\mathrm{Lo2}{}^2\left(1+\frac{1}{SNR_\mathrm{Sp}{}^2}\right)\overline{P_\mathrm{Sp2}(z)}^2$$
$$+16\mathcal{R}_\mathrm{p}{}^4 P_\mathrm{Lo1}\overline{P_\mathrm{Sp1}(z)}P_\mathrm{Lo2}\overline{P_\mathrm{Sp2}(z)}$$
$$= \frac{1}{2}\left(3+\frac{1}{SNR_\mathrm{Sp}{}^2}\right)\mathcal{R}_\mathrm{p}{}^4 P_\mathrm{Lo}{}^2\overline{P_\mathrm{Sp}(z)}^2 \tag{S84}$$

Finally, the variance of $\mathrm{LPF}\{I_s(z)^2\}$, designated as $\sigma_{s^2}(z)^2$, can be computed according to its statistic definition:

$$\sigma_{s^2}(z)^2 = \overline{\mathrm{LPF}^2\{I_s(z)^2\}} - \mu_{s^2}(z)^2 = \frac{1}{2}\left(1+\frac{1}{SNR_\mathrm{Sp}{}^2}\right)\mathcal{R}_\mathrm{p}{}^4 P_\mathrm{Lo}{}^2\overline{P_\mathrm{Sp}(z)}^2 \tag{S85}$$

2) The term of $\mathrm{LPF}\{2I_s(z)I_e(z)\}$. Since the BPF bandwidth is narrower than the system acquisition bandwidth, $I_e(z)$ can be considered as a narrowband random noise, which can be decomposed into its in-phase and quadrature components[11]:

$$I_e(z) = I_e^\mathrm{I}(z)\cos[H(z)] - I_e^\mathrm{Q}(z)\sin[H(z)] \tag{S86}$$

where $I_e^\mathrm{I}(z)$ and $I_e^\mathrm{Q}(z)$ stand for the in-phase and quadrature components of $I_e(z)$, respectively. These two independent components both follow zero-mean normal distributions with identical

variance to $I_e(z)$. By substituting Eq. (S76) and Eq. (S86) into the $2I_s(z)I_e(z)$ and removing high-frequency components, we obtain:

$$\begin{aligned}\mathrm{LPF}\{2I_s(z)I_e(z)\} &= 2\mathcal{R}_\mathrm{p}I_e^\mathrm{I}(z)\sqrt{P_\mathrm{Lo1}P_\mathrm{Sp1}(z)}\cos[\Phi_1(z)] + 2\mathcal{R}_\mathrm{p}I_e^\mathrm{Q}(z)\sqrt{P_\mathrm{Lo1}P_\mathrm{Sp1}(z)}\sin[\Phi_1(z)] \\ &+2\mathcal{R}_\mathrm{p}I_e^\mathrm{I}(z)\sqrt{P_\mathrm{Lo2}P_\mathrm{Sp2}(z)}\cos[\Phi_2(z)] + 2\mathcal{R}_\mathrm{p}I_e^\mathrm{Q}(z)\sqrt{P_\mathrm{Lo2}P_\mathrm{Sp2}(z)}\sin[\Phi_2(z)]\end{aligned} \quad \text{(S87)}$$

The expectation value of $\mathrm{LPF}\{2I_s(z)I_e(z)\}$, designated as $\mu_{2se}(z)$, can be calculated as:

$$\mu_{2se}(z) = 0 \quad \text{(S88)}$$

The variance of $\mathrm{LPF}\{2I_s(z)I_e(z)\}$, designated as $\sigma_{2se}(z)^2$, can then be derived using its statistical definition:

$$\begin{aligned}\sigma_{2se}(z)^2 &= \overline{\mathrm{LPF}^2\{2I_s(z)I_e(z)\}} - \mu_{2se}(z)^2 \\ &= 4\mathcal{R}_\mathrm{p}{}^2\left[P_\mathrm{Lo1}\overline{P_\mathrm{Sp1}(z)} + P_\mathrm{Lo2}\overline{P_\mathrm{Sp2}(z)}\right]\sigma_e{}^2B_\mathrm{BPF} = 2\mathcal{R}_\mathrm{p}{}^2P_\mathrm{Lo}\overline{P_\mathrm{Sp}(z)}\sigma_e{}^2B_\mathrm{BPF}\end{aligned} \quad \text{(S89)}$$

3) The term of LPF$\{I_e(z)^2\}$. By substituting Eq. (S86) into $\mathrm{LPF}\{I_e(z)^2\}$, we obtain:

$$\mathrm{LPF}\{I_e(z)^2\} = \frac{1}{2}I_e^\mathrm{I}(z)^2 + \frac{1}{2}I_e^\mathrm{Q}(z)^2 \quad \text{(S90)}$$

According to the calculation of multiple moments of a normal distribution[12], it can be obtained that:

$$\overline{I_e^\mathrm{I}(z)^2} = \overline{I_e^\mathrm{Q}(z)^2} = \sigma_e{}^2B_\mathrm{BPF} \quad \text{(S91)}$$

and

$$\overline{I_e^\mathrm{I}(z)^4} = \overline{I_e^\mathrm{Q}(z)^4} = 3\sigma_e{}^4B_\mathrm{BPF}{}^2 \quad \text{(S92)}$$

The expectation value and variance of $\mathrm{LPF}\{I_e(z)^2\}$, designated as $\mu_{e^2}(z)$ and $\sigma_{e^2}(z)^2$, respectively, can then be derived as:

$$\mu_{e^2}(z) = \overline{\mathrm{LPF}\{I_e(z)^2\}} = \sigma_e{}^2B_\mathrm{BPF} \quad \text{(S93)}$$

$$\sigma_{e^2}(z)^2 = \mathrm{D}\left\{\frac{1}{2}I_e^\mathrm{I}(z)^2\right\} + \mathrm{D}\left\{\frac{1}{2}I_e^\mathrm{Q}(z)^2\right\} = (\sigma_e{}^2B_\mathrm{BPF})^2 \quad \text{(S94)}$$

All above-derived statistic parameters (expectation values and variances of the three terms in Eq. (S78)) are summarized in Table S2 to eventually model the SNR of the single-pulse BOTDR with PMF. Finally, practically subtracting the bias arising from $\mu_{e^2}(z)$ (i.e., the expectation value of $\mathrm{LPF}\{I_e(z)^2\}$) in actual measurement, the SNR at each fiber position z can be expressed as:

$$\begin{aligned}\mathrm{SNR}\{r_\mathrm{Sg}^\mathrm{PM}(z)\} &= \frac{\mu_{s^2}(z)}{\sqrt{\sigma_{s^2}(z)^2 + \sigma_{2se}(z)^2 + \sigma_{e^2}(z)^2}} \\ &= \frac{\mathcal{R}_\mathrm{p}{}^2P_\mathrm{Lo}\overline{P_\mathrm{Sp}(z)}}{\sqrt{\frac{1}{2}\left(1+\frac{1}{SNR_\mathrm{Sp}{}^2}\right)\mathcal{R}_\mathrm{p}{}^4P_\mathrm{Lo}{}^2\overline{P_\mathrm{Sp}(z)}^2 + 2\mathcal{R}_\mathrm{p}{}^2P_\mathrm{Lo}\overline{P_\mathrm{Sp}(z)}\sigma_e{}^2B_\mathrm{BPF} + \sigma_e{}^4B_\mathrm{BPF}{}^2}}\end{aligned} \quad \text{(S95)}$$

For a 400 m PMF with a 10 ns pulse duration and a pump power of 26 dBm, **Fig. S13** presents the contributions of the three noise terms and the total noise described in the denominator of Eq. (S95) as a function of the sensing distance, along with the corresponding experimental results. The good agreement between the total theoretical noise and the experimental measurements can be clearly observed.

Since the noise behavior is nearly uniform along the short fiber due to negligible loss, we select two pulse durations (10 ns and 100 ns) and plot the mean signal, individual noise standard deviations and SNR at a fixed fiber position as a function of the pulse power, as shown in the first two rows of **Fig. S14** (the last two rows are for the SMF case, described in **Note S5** later). For all fiber types and pulse durations, the vertical axis scales are kept consistent. Following the style used in other experiments in the manuscript, the noise STD subplot explicitly shows the theoretical contributions of each noise term (with “detection noise” corresponding to shot noise). The first two rows of **Fig. S14** demonstrate good agreement between theory and experiment. From these profiles, it is clear that the SpBS noise increases with the pulse power, while larger pulse widths require lower pulse powers for SpBS noise to dominate.

Supplementary Note S5. Modelling the SNR of single-pulse BOTDR using standard single-mode fiber

Here the SNR model of a conventional BOTDR sensing system using standard single model fiber (SMF) is established. The system employs a polarization scrambler (PSc) to alleviate polarization fading (i.e., introducing polarization noise). Compared to the PMF case in **Note S4**, this scenario incorporates polarization-related effects[10,13,14]. Consequently, the distance-domain signal photocurrent output from the BPF is expressed as:

$$I_s(z) = 2\mathcal{R}_{\mathrm{p}}\zeta_{\mathrm{Pol}}(z)\sqrt{P_{\mathrm{Lo1}}P_{\mathrm{Sp1}}(z)}\cos[H(z)+\Phi_1(z)] \\ +2\mathcal{R}_{\mathrm{p}}\zeta_{\mathrm{pol}}(z)\sqrt{P_{\mathrm{Lo2}}P_{\mathrm{Sp2}}(z)}\cos[H(z)+\Phi_2(z)] \quad (S96)$$

where $\zeta_{\mathrm{Pol}}(z) = \sqrt{k_{\mathrm{Pol}}\cos^2[\theta(z)] + \frac{1}{2}(1-k_{\mathrm{Pol}})}$ is a polarization-related term considering the influence of self-polarization-scrambling effect **(Note S6)**. The angle $\theta(z)$ denotes the local relative polarization rotation of the SpBS with respect to the OLO, varying within the range $[0, \pi/2]$, and k_{Pol} is introduced to adopt the statistical properties of $\cos^2[\theta(z)]$ accounting for self-polarization-scrambling effect.

Following a mathematical process similar to that in **Note S4**, we model the SNR behavior of BOTDR response (power envelope signal, designated as $r_{\mathrm{Sg}}^{\mathrm{SMF}}(z)$), incorporating the effects of polarization noise.

1) The term of LPF$\{\boldsymbol{I_s(z)^2}\}$. Based on Eq. (S96), the term of LPF$\{I_s(z)^2\}$ can be expressed as:

$$\mathrm{LPF}\{I_s(z)^2\} = 2\mathcal{R}_{\mathrm{p}}{}^2\zeta_{\mathrm{Pol}}(z)^2\Big\{P_{\mathrm{Lo1}}P_{\mathrm{Sp1}}(z) + P_{\mathrm{Lo2}}P_{\mathrm{Sp2}}(z) \\ +2\sqrt{P_{\mathrm{Lo1}}P_{\mathrm{Sp1}}(z)P_{\mathrm{Lo2}}P_{\mathrm{Sp2}}(z)}\cos[\Delta\Phi(z)]\Big\} \quad (S97)$$

According to the fact that $\cos^2[\theta(z)]$ varies within the range [0, 1], following a uniform distribution over multiple measurements, we can obtain the statistical properties of $\zeta_{\mathrm{Pol}}(z)$ as:

$$\overline{\zeta_{\mathrm{Pol}}(z)^2} = \frac{1}{2} \quad (S98)$$

$$\mathrm{D}\{\zeta_{\mathrm{Pol}}(z)^2\} = k_{\mathrm{Pol}}{}^2\mathrm{D}\{\cos^2[\theta(z)]\} = \frac{1}{12}k_{\mathrm{Pol}}{}^2 \quad (S99)$$

Then, the expectation value of LPF$\{I_s(z)^2\}$, i.e., $\mu_{s^2}(z)$, can be calculated as:

$$\mu_{s^2}(z) = \overline{\mathrm{LPF}\{I_s(z)^2\}} = \mathcal{R}_{\mathrm{p}}{}^2\left[P_{\mathrm{Lo1}}\overline{P_{\mathrm{Sp1}}(z)} + P_{\mathrm{Lo2}}\overline{P_{\mathrm{Sp2}}(z)}\right] = \frac{1}{2}\mathcal{R}_{\mathrm{p}}{}^2P_{\mathrm{Lo}}\overline{P_{\mathrm{Sp}}(z)} \quad (S100)$$

Then, according to the relationships of Eqs. (S82)-(S83), and:

$$\overline{\zeta_{\mathrm{Pol}}{}^4[\theta(z)]} = \mathrm{D}\{\zeta_{\mathrm{Pol}}(z)^2\} + \overline{\zeta_{\mathrm{Pol}}(z)^2}^2 = \frac{k_{\mathrm{Pol}}{}^2+3}{12} \quad (S101)$$

we obtain:

$$
\begin{aligned}
\overline{\mathrm{LPF}^2\{I_s(z)^2\}} &= 4\mathcal{R}_{\mathrm{p}}{}^4\frac{k_{\mathrm{Pol}}{}^2+3}{12}\left\{P_{\mathrm{Lo1}}{}^2\overline{P_{\mathrm{Sp1}}(z)^2}+P_{\mathrm{Lo2}}{}^2\overline{P_{\mathrm{Sp2}}(z)^2}+4P_{\mathrm{Lo1}}\overline{P_{\mathrm{Sp1}}(z)}P_{\mathrm{Lo2}}\overline{P_{\mathrm{Sp2}}(z)}\right\} \\
&= 4\mathcal{R}_{\mathrm{p}}{}^4\frac{k_{\mathrm{Pol}}{}^2+3}{12}\left\{P_{\mathrm{Lo1}}{}^2\left(1+\frac{1}{SNR_{\mathrm{Sp}}{}^2}\right)\overline{P_{\mathrm{Sp1}}(z)}^2\right. \\
&\left.+P_{\mathrm{Lo2}}{}^2\left(1+\frac{1}{SNR_{\mathrm{Sp}}{}^2}\right)\overline{P_{\mathrm{Sp2}}(z)}^2+4P_{\mathrm{Lo1}}\overline{P_{\mathrm{Sp1}}(z)}P_{\mathrm{Lo2}}\overline{P_{\mathrm{Sp2}}(z)}\right\} \\
&= \frac{k_{\mathrm{Pol}}{}^2+3}{24}\left(3+\frac{1}{SNR_{\mathrm{Sp}}{}^2}\right)\mathcal{R}_{\mathrm{p}}{}^4P_{\mathrm{Lo}}{}^2\overline{P_{\mathrm{Sp}}(z)}^2 \qquad \text{(S102)}
\end{aligned}
$$

Therefore, the variance of $\mathrm{LPF}\{I_s(z)^2\}$, i.e., $\sigma_{s^2}(z)^2$ can be calculated according to its statistic definition:

$$
\begin{aligned}
\sigma_{s^2}(z)^2 &= \overline{\mathrm{LPF}^2\{I_s(z)^2\}}-\mu_{s^2}(z)^2 \\
&= \frac{3SNR_{\mathrm{Sp}}{}^2\left(k_{\mathrm{Pol}}{}^2+1\right)+k_{\mathrm{Pol}}{}^2+3}{24SNR_{\mathrm{Sp}}{}^2}\mathcal{R}_{\mathrm{p}}{}^4P_{\mathrm{Lo}}{}^2\overline{P_{\mathrm{Sp}}(z)}^2 \qquad \text{(S103)}
\end{aligned}
$$

2) The term of $\mathrm{LPF}\{2I_s(z)I_e(z)\}$. Similar to the calculation process from Eq. (S86) to Eq. (S89), the expectation value and variance of $\mathrm{LPF}\{2I_s(z)I_e(z)\}$, designated as $\mu_{2se}(z)$ and $\sigma_{2se}(z)^2$, respectively, can be derived based on their respective statistic definition:

$$
\mu_{2se}(z) = 0 \qquad \text{(S104)}
$$

$$
\begin{aligned}
\sigma_{2se}(z)^2 &= \overline{\mathrm{LPF}^2\{2I_s(z)I_e(z)\}}-\mu_{2se}(z)^2 \\
&= 2\mathcal{R}_{\mathrm{p}}{}^2\left[P_{\mathrm{Lo1}}\overline{P_{\mathrm{Sp1}}(z)}+P_{\mathrm{Lo2}}\overline{P_{\mathrm{Sp2}}(z)}\right]\sigma_e{}^2 \\
&= \mathcal{R}_{\mathrm{p}}{}^2P_{\mathrm{Lo}}\overline{P_{\mathrm{Sp}}(z)}\sigma_e{}^2B_{\mathrm{BPF}} \qquad \text{(S105)}
\end{aligned}
$$

3) The term of $\mathrm{LPF}\{I_e(z)^2\}$. Since the statistical parameters of $\mathrm{LPF}\{I_e(z)^2\}$ – namely its expectation and variance – are unaffected by polarization, the analysis of $\mathrm{LPF}\{I_e(z)^2\}$ in this note is consistent with that provided in **Note S4**.

For the SMF case, the statistical parameters of the three terms in an expression similar to Eq. (S78) are summarized in Table S3, and the SNR at each fiber position z can be represented as:

$$
\begin{aligned}
\mathrm{SNR}\{r_{\mathrm{Sg}}^{\mathrm{SMF}}(z)\} &= \frac{\mu_{s^2}(z)}{\sqrt{\sigma_{s^2}(z)^2+\sigma_{2se}(z)^2+\sigma_{e^2}(z)^2}} \\
&= \frac{\frac{1}{2}\mathcal{R}_{\mathrm{p}}{}^2P_{\mathrm{Lo}}\overline{P_{\mathrm{Sp}}(z)}}{\sqrt{\frac{3SNR_{\mathrm{Sp}}{}^2\left(k_{\mathrm{Pol}}{}^2+1\right)+k_{\mathrm{Pol}}{}^2+3}{24SNR_{\mathrm{Sp}}{}^2}\mathcal{R}_{\mathrm{p}}{}^4P_{\mathrm{Lo}}{}^2\overline{P_{\mathrm{Sp}}(z)}^2+\mathcal{R}_{\mathrm{p}}{}^2P_{\mathrm{Lo}}\overline{P_{\mathrm{Sp}}(z)}\sigma_e{}^2B_{\mathrm{BPF}}+\sigma_e{}^4B_{\mathrm{BPF}}{}^2}} \qquad \text{(S106)}
\end{aligned}
$$

For a 1.9 km SMF with pulse durations of 10 ns and 100 ns, the mean signal, individual noise standard deviations, and SNR profiles as a function of the pulse power are shown in the last two rows of **Fig. S14**. Compared with the PMF case (first two rows of **Fig. S14**), the noise exhibits a similar trend, and the overall SNR is as expected lower due to the combination effect of polarization noise and SpBS noise.

Supplementary Note S6. Analysis of the self-polarization-scrambling effect

Polarization fading is a typical phenomenon in SMF-based BOTDR systems, manifesting as distance-domain signal fluctuations due to the random state-of-polarization (SOP) mismatch between the spontaneous Brillouin scattering (SpBS) signal propagating through the single-mode fiber (SMF) and the local oscillator (OLO)[13]. Mathematically, this effect is commonly described by a random polarization-related term $\cos^2[\theta(z)]$, which follows a uniform distribution over the interval [0,1], as illustrated by the simulated pink curves in **Fig. S15**. This leads to envelope signal fluctuations ranging from zero (when the SOPs of SpBS and OLO are orthogonal) to a maximum (when they are aligned) in standard SMFs.

Here, we identify and analyze a mitigating mechanism – the self-polarization-scrambling effect – arising from the non-uniform SOP across the pump pulse duration caused by fiber birefringence. This introduces a weak scrambling of polarization within the pulse itself, effectively averaging out polarization fluctuations and thereby reducing the severity of polarization fading. Mathematically, this effect can be described by a modified polarization-related expression $\zeta_{\text{Pol}}(z)^2 = k_{\text{Pol}} \cos^2[\theta(z)] + \frac{1}{2}(1 - k_{\text{Pol}})$, which reduces the variance of $\cos^2[\theta(z)]$ by a factor of ${k_{\text{Pol}}}^2$, $k_{\text{Pol}} \in [0, 1]$ being a spatial-resolution-dependent parameter introduced to quantify the degree of scrambling. A wider spatial resolution (SR) results in stronger SOP averaging and thus a smaller k_{Pol}, as shown by the simulated blue curves in **Fig. S15**.

1. Experimental quantification of k_{Pol} for different spatial resolutions (SRs)

We experimentally quantify k_{Pol} for SRs of 1 m, 2 m, 6 m, and 10 m by evaluating how much polarization fading is mitigated under each condition. Using a standard BOTDR setup[10] with a 1.9 km-long SMF and deliberately removing the polarization scrambler (PSc) to preserve polarization fading, we acquire BOTDR traces for each SR. The pulse peak power is fixed at 31 dBm, and each trace is averaged 1,000 times to ensure high SNR and negligible detection noise.

The resulting BOTDR traces are shown in **Fig. S16a–d**, each displaying two blue dashed lines indicating the upper and lower signal bounds. A red dashed line is also plotted, representing the theoretical maximum in the absence of self-polarization-scrambling, calculated by doubling the local mean of each trace. The value of k_{Pol} is then computed as the ratio between the peak-to-peak span of the blue dashed lines and the corresponding red dashed line. For the examined SRs of 1 m, 2 m, 6 m, and 10 m, the corresponding k_{Pol} values are characterized as 0.88, 0.83, 0.78 and 0.69.

2. Layered SNR behavior with a polarization scrambler (PSc)

Here we clarify that, Eq. (S106) that incorporates k_{Pol} to account for the self-polarization-scrambling effect, provides an accurate estimation of the minimum expected SNR, a standard metric used to qualify distributed sensing systems. This lower bound is used for the comparison shown in **Fig. 6e** of the main text.

However, as illustrated by **Fig. S16e-h**, which shows the SNR profiles for the four SRs when a polarization scrambler is included, the actual SNR at certain fiber positions may exceed this predicted minimum. While these localized SNR increases are not critical from a sensing-performance perspective, they are theoretically noteworthy. This phenomenon occurs when the pump pulse passes through fiber segments exhibiting sharp SOP transitions due to birefringence. In these regions, additional SOP averaging – on top of that induced by the PSc – leads to further suppression of polarization noise[14]. The corresponding SNR histograms, shown in **Fig. S16e-h** are illustrated by **Fig. S16i-l**, indicate that most data conform to the expected distribution defined by Eq. (S106), following a standard normal profile.

Supplementary Note S7. BOTDR based on polarization diversity coherent receiver

In this note, we demonstrate that even when employing a polarization-diversity coherent receiver (PDCR)[15] – designed to mitigate polarization-induced noise by combining BOTDR responses from orthogonal states of polarization (SOPs) – the improvement in signal-to-noise ratio (SNR) remains limited due to the fundamental constraint imposed by spontaneous Brillouin scattering (SpBS) noise.

Replacing the standard detection scheme used in **Note S6** with a PDCR configuration, we conducted BOTDR measurements at a spatial resolution (SR) of 2 m. The envelope signal for each PDCR channel was extracted via digital post-processing of the detected beat signals, with 2,500 trace averages applied. The resulting envelope traces for the two orthogonal polarization channels are shown as pink and blue curves in **Fig. S17a**, along with their combined (summed) envelope, represented by the green curve. Corresponding SNR profiles for these traces are plotted in **Fig. S17b**.

Focusing on the green curve, which reflects the effective SNR of the PDCR-based BOTDR, we observe only a marginal difference compared to the case using a polarization scrambler (PSc), as shown in **Fig. S16f**. In both cases, the overall SNR is limited not by polarization fading, but by the intrinsic noise of the SpBS process.

Supplementary Note S8. Modelling and experimental verification of the SNR in coded-pulse BOTDR using standard SMF

In this note, we demonstrate that the proposed SpBS noise framework is not limited to standard single-pulse BOTDR, but can also be extended to evaluate the performance of more advanced BOTDR techniques, such as coded-pulse BOTDR, thereby revising the conventional assumptions regarding their performance limits.

While pulse coding has been widely regarded as an effective strategy to enhance SNR in comparison to single-pulse approaches, our theoretical analysis and experimental verification reveal that, in the BOTDR context, the actual improvement is limited. This is due to the combined influence of spontaneous Brillouin scattering (SpBS) noise and polarization-induced noise, both of which fundamentally constrain the achievable SNR. Thus, contrary to common expectations, pulse coding yields only marginal performance gains in BOTDR systems employing standard single-mode fiber (SMF).

1. SNR model of coded-pulse BOTDR

Here we leverage our SpBS noise framework to establish the SNR model of coded-pulse BOTDR. Since the fact that all ‘0’ elements in the coded sequence do not contribute to the signal carrying Brillouin sensing information, only the contributions of the ‘1’ elements are considered here. For aperiodic codes[16-19], as the fiber attenuation over the duration of the coding sequence is almost negligible, the total optical field of the coded SpBS, including Stokes $\overrightarrow{E_{\mathrm{Sp1}}}(t)$ and anti-Stokes $\overrightarrow{E_{\mathrm{Sp2}}}(t)$, can be treated as a superposition of the fields contributed by each ‘1’ element:

$$\overrightarrow{E_{\mathrm{Sp1}}}(t) \approx \sum_{i=1}^{N_1} \overrightarrow{E_{\mathrm{Sp1}}^{i}}(t)$$
$$= \sum_{i=1}^{N_1} \left\{ \hat{x} \cos[\theta_i(t)]\, e^{j\varphi_{\mathrm{Sp1}}^{xi}(t)} + \hat{y} \sin[\theta_i(t)]\, e^{j\varphi_{\mathrm{Sp1}}^{yi}(t)} \right\} E_{\mathrm{Sp1}}^{i}(t) e^{\mathrm{j}2\pi f_{\mathrm{Sp1}}(t) t} \quad \text{(S107)}$$

$$\overrightarrow{E_{\mathrm{Sp2}}}(t) \approx \sum_{i=1}^{N_1} \overrightarrow{E_{\mathrm{Sp2}}^{i}}(t)$$
$$= \sum_{i=1}^{N_1} \left\{ \hat{x} \cos[\theta_i(t)]\, e^{j\varphi_{\mathrm{Sp2}}^{xi}(t)} + \hat{y} \sin[\theta_i(t)]\, e^{j\varphi_{\mathrm{Sp2}}^{yi}(t)} \right\} E_{\mathrm{Sp2}}^{i}(t) e^{\mathrm{j}2\pi f_{\mathrm{Sp2}}(t) t} \quad \text{(S108)}$$

where $\hat{x}$ and $\hat{y}$ stand for the polarization direction of the OLO light and its orthogonal direction, respectively; N_1 represents the number of “1”s elements in the coded pulse sequence; i is the index of each ‘1’ element after removing all ‘0’ elements in the coded sequence; $\overrightarrow{E_{\mathrm{Sp1}}^{i}}(t)$ and $\overrightarrow{E_{\mathrm{Sp2}}^{i}}(t)$ are the fields of the Stokes and anti-Stokes components contributed by the i-th ‘1’ element, respectively. The local relative polarization rotation ($\theta_i(t)$), the random phase differences ($\varphi_{\mathrm{Sp1}}^{xi}(t)$, $\varphi_{\mathrm{Sp1}}^{yi}(t)$, $\varphi_{\mathrm{Sp2}}^{xi}(t)$, $\varphi_{\mathrm{Sp2}}^{yi}(t)$), and the SpBS amplitudes ($E_{\mathrm{Sp1}}^{i}(t)$, $E_{\mathrm{Sp2}}^{i}(t)$) are i-dependent.

Following a derivation similar to that in ref. 10, and considering the self-polarization-scrambling effect as discussed in Note S6, the BPF output signal photocurrent in distance domain can be obtained as:

$$I_s(z) = 2\mathcal{R}_\mathrm{p}\sum_{i=1}^{N_1}\zeta_\mathrm{Pol}^i(z)\left\{\sqrt{P_\mathrm{Lo1}P_\mathrm{Sp1}^i(z)}\cos\left[H(z)+\Phi_1^i(z)\right]\right.$$
$$\left.+\sqrt{P_\mathrm{Lo2}P_\mathrm{Sp2}^i(z)}\cos\left[H(z)+\Phi_2^i(z)\right]\right\} \stackrel{\mathrm{def}}{=} 2\mathcal{R}_\mathrm{p}\sum_{i=1}^{N_1}\Upsilon_i(z) \quad \text{(S109)}$$

where $\zeta_\mathrm{Pol}^i(z) = \sqrt{k_\mathrm{Pol}\cos^2[\theta_i(z)] + \frac{1}{2}(1-k_\mathrm{Pol})}$, similar with that in Eq. (S96) of Note S5. $\Phi_1^i(t) = \varphi_\mathrm{Sp1}^{xi}(t) - \pi/2$, $\Phi_2^i(t) = \varphi_\mathrm{Sp2}^{xi}(t) - \pi/2$. $P_\mathrm{Sp1}^i(z) \propto E_\mathrm{Sp1}^i(z)^2$ and $P_\mathrm{Sp2}^i(z) \propto E_\mathrm{Sp2}^i(z)^2$ represent the Stokes and anti-Stokes SpBS optical powers, respectively, having approximately equal mathematical expectations, that is, $\overline{P_\mathrm{Sp1}^i(z)} = \overline{P_\mathrm{Sp2}^i(z)} = \overline{P_\mathrm{Sp}^i(z)}/2$. The mathematical expectation of the total SpBS power corresponding to each element '1', $\overline{P_\mathrm{Sp}^i(z)}$, can be expressed by Eq. (S77). $\Upsilon_i(z)$ is used to denote the term inside the summation for the convenience of the following mathematical derivation:

$$\Upsilon_i(z) = \zeta_\mathrm{Pol}^i(z)\sqrt{P_\mathrm{Lo1}P_\mathrm{Sp1}^i(z)}\cos\left[H(z)+\Phi_1^i(z)\right]$$
$$+\zeta_\mathrm{Pol}^i(z)\sqrt{P_\mathrm{Lo2}P_\mathrm{Sp2}^i(z)}\cos\left[H(z)+\Phi_2^i(z)\right] \quad \text{(S110)}$$

For the coded-pulse BOTDR system, we further model the SNR behavior of the decoded response (decoded power envelope signal, designated as $r_\mathrm{Decode}^\mathrm{SMF}(z)$) taking into account the polarization noise. We start with the analysis on the SNR of the coded response $r_\mathrm{Code}^\mathrm{SMF}(z)$, following a mathematical process similar to that in **Note S4** and **Note S5**.

1) The term of LPF$\{I_s(z)^2\}$. Squaring Eq. (S109), we get:

$$I_s(z)^2 = 4{\mathcal{R}_\mathrm{p}}^2\left\{\sum_{i=1}^{N_1}\Upsilon_i(z)\right\}^2 = 4{\mathcal{R}_\mathrm{p}}^2\sum_{i=1}^{N_1}\Upsilon_i(z)^2 + 4{\mathcal{R}_\mathrm{p}}^2\sum_{i\neq k}^{N_1(N_1-1)/2}2\Upsilon_i(z)\Upsilon_k(z) \quad \text{(S111)}$$

Since both summation and low-pass filtering are linear processes, LPF$\{I_s(z)^2\}$ can be expressed as:

$$\mathrm{LPF}\{I_s(z)^2\} = 4{\mathcal{R}_\mathrm{p}}^2\mathrm{LPF}\left\{\sum_{i=1}^{N_1}\Upsilon_i(z)^2\right\} + 4{\mathcal{R}_\mathrm{p}}^2\mathrm{LPF}\left\{\sum_{i\neq k}^{\frac{N_1(N_1-1)}{2}}2\Upsilon_i(z)\Upsilon_k(z)\right\}$$
$$= 4{\mathcal{R}_\mathrm{p}}^2\sum_{i=1}^{N_1}\mathrm{LPF}\{\Upsilon_i(z)^2\} + 4{\mathcal{R}_\mathrm{p}}^2\sum_{i\neq k}^{N_1(N_1-1)/2}\mathrm{LPF}\{2\Upsilon_i(z)\Upsilon_k(z)\} \quad \text{(S112)}$$

Considering that the covariance of the last two terms in Eq. (S112) is zero, the expectation value and variance of LPF$\{I_s(z)^2\}$, designated as $\mu_{s^2}(z)$ and $\sigma_{s^2}(z)^2$, respectively, can be expressed as:

$$\mu_{s^2}(z) = \overline{\mathrm{LPF}\{I_s(z)^2\}} = 4{\mathcal{R}_\mathrm{p}}^2\sum_{i=1}^{N_1}\overline{\mathrm{LPF}\{\Upsilon_i(z)^2\}} + 4{\mathcal{R}_\mathrm{p}}^2\sum_{i\neq k}^{\frac{N_1(N_1-1)}{2}}\overline{\mathrm{LPF}\{2\Upsilon_i(z)\Upsilon_k(z)\}} \quad \text{(S113)}$$

$$\sigma_{s^2}(z)^2 = \mathrm{D}\{\mathrm{LPF}\{I_s(z)^2\}\}$$
$$= 16\mathcal{R}_\mathrm{p}{}^4\left\{\sum_{i=1}^{N_1}\mathrm{D}\{\mathrm{LPF}\{\Upsilon_i(z)^2\}\} + \sum_{i\neq k}^{\frac{N_1(N_1-1)}{2}}\mathrm{D}\{\mathrm{LPF}\{2\Upsilon_i(z)\Upsilon_k(z)\}\}\right\} \quad \text{(S114)}$$

This necessitates to derive the statistical properties of $\mathrm{LPF}\{\Upsilon_i(z)^2\}$ and $\mathrm{LPF}\{2\Upsilon_i(z)\Upsilon_k(z)\}$.

Firstly, $\Upsilon_i(z)^2$ with a low-pass filtering process can be expressed as :

$$\mathrm{LPF}\{\Upsilon_i(z)^2\} = \frac{1}{2}\zeta_\mathrm{Pol}^i(z)^2 P_\mathrm{Lo1}P_\mathrm{Sp1}^i(z) + P_\mathrm{Lo2}P_\mathrm{Sp2}^i(z)$$
$$+\zeta_\mathrm{Pol}^i(z)^2\sqrt{P_\mathrm{Lo1}P_\mathrm{Sp1}^i(z)P_\mathrm{Lo2}P_\mathrm{Sp2}^i(z)}\cos\left[\Phi_1^i(z) - \Phi_2^i(z)\right] \quad \text{(S115)}$$

where $\zeta_\mathrm{Pol}^i(z)^2$ follows the same statistical properties described in Eq. (S98), Eq. (S99) and Eq. (S101). Substituting the mathematical expectations of $P_\mathrm{Sp1}^i(z)$ and $P_\mathrm{Sp2}^i(z)$, ($\overline{P_\mathrm{Sp1}(z)}$ and $\overline{P_\mathrm{Sp2}(z)}$), yielding:

$$\overline{\mathrm{LPF}\{\Upsilon_i(z)^2\}} = \frac{1}{4}\left[P_\mathrm{Lo1}\overline{P_\mathrm{Sp1}(z)} + P_\mathrm{Lo2}\overline{P_\mathrm{Sp2}(z)}\right] = \frac{1}{8}P_\mathrm{Lo}\overline{P_\mathrm{Sp}(z)} \quad \text{(S116)}$$

Similar with Eqs. (S82) and (S83), there is a relationship of $\overline{P_\mathrm{Sp1}^i(z)^2} = \left(1 + \frac{1}{SNR_\mathrm{Sp}{}^2}\right)\overline{P_\mathrm{Sp1}(z)}^2$ and $\overline{P_\mathrm{Sp2}^i(z)^2} = \left(1 + \frac{1}{SNR_\mathrm{Sp}{}^2}\right)\overline{P_\mathrm{Sp2}(z)}^2$, thus the mathematical expectation of $\mathrm{LPF}^2\{\Upsilon_i(z)^2\}$ can be calculated as:

$$\overline{\mathrm{LPF}^2\{\Upsilon_i(z)^2\}} = \frac{1}{4}\frac{k_\mathrm{Pol}{}^2+3}{12}\left\{P_\mathrm{Lo1}{}^2\left(1 + \frac{1}{SNR_\mathrm{Sp}{}^2}\right)\overline{P_\mathrm{Sp1}(z)}^2\right.$$
$$\left.+P_\mathrm{Lo2}{}^2\left(1 + \frac{1}{SNR_\mathrm{Sp}{}^2}\right)\overline{P_\mathrm{Sp2}(z)}^2 + 4P_\mathrm{Lo1}\overline{P_\mathrm{Sp1}(z)}P_\mathrm{Lo2}\overline{P_\mathrm{Sp2}(z)}\right\}$$
$$= \frac{k_\mathrm{Pol}{}^2+3}{384}\left(3 + \frac{1}{SNR_\mathrm{Sp}{}^2}\right)P_\mathrm{Lo}{}^2\overline{P_\mathrm{Sp}(z)}^2 \quad \text{(S117)}$$

Therefore, the variance of $\mathrm{LPF}\{\Upsilon_i(z)^2\}$ can be calculated as:

$$\mathrm{D}\{\mathrm{LPF}\{\Upsilon_i(z)^2\}\} = \overline{\mathrm{LPF}^2\{\Upsilon_i(z)^2\}} - \overline{\mathrm{LPF}\{\Upsilon_i(z)^2\}}^2$$
$$= \left[\frac{k_\mathrm{Pol}{}^2+3}{384}\left(3 + \frac{1}{SNR_\mathrm{Sp}{}^2}\right) - \frac{1}{64}\right]P_\mathrm{Lo}{}^2\overline{P_\mathrm{Sp}(z)}^2 \quad \text{(S118)}$$

Next, the mathematical expectation and variance of $\mathrm{LPF}\{2\Upsilon_i(z)\Upsilon_k(z)\}$ are calculated as:

$$\overline{\mathrm{LPF}\{2\Upsilon_i(z)\Upsilon_k(z)\}} = 0 \quad \text{(S119)}$$

$$\mathrm{D}\{\mathrm{LPF}\{2\Upsilon_i(z)\Upsilon_k(z)\}\} = \overline{\mathrm{LPF}^2\{2\Upsilon_i(z)\Upsilon_k(z)\}} - \overline{\mathrm{LPF}\{2\Upsilon_i(z)\Upsilon_k(z)\}}^2$$
$$= \frac{1}{8}\left[P_\mathrm{Lo1}\overline{P_\mathrm{Sp1}(z)} + P_\mathrm{Lo2}\overline{P_\mathrm{Sp1}(z)}\right]^2 = \frac{1}{32}P_\mathrm{Lo}{}^2\overline{P_\mathrm{Sp}(z)}^2 \quad \text{(S120)}$$

Finally, based on Eq. (S113) and Eq. (S114), $\mu_{s^2}(z)$ and $\sigma_{s^2}(z)^2$ can be obtained as:

$$\mu_{s^2}(z) = 4\mathcal{R}_\mathrm{p}{}^2\sum_{i=1}^{N_1}\frac{1}{8}P_\mathrm{Lo}\overline{P_\mathrm{Sp}(z)} = \frac{1}{2}N_1\mathcal{R}_\mathrm{p}{}^2P_\mathrm{Lo}\overline{P_\mathrm{Sp}(z)} \quad \text{(S121)}$$

$$\sigma_{s^2}(z)^2 = \frac{1}{4}\mathcal{R}_\mathrm{p}{}^4\sum_{i=1}^{N_1}\left[\frac{k_\mathrm{Pol}{}^2+3}{6}\left(3+\frac{1}{SNR_\mathrm{Sp}{}^2}\right)-1\right]P_\mathrm{Lo}{}^2\overline{P_\mathrm{Sp}(z)}^2 + \frac{1}{2}\mathcal{R}_\mathrm{p}{}^4\sum_{i\neq k}^{\frac{N_1(N_1-1)}{2}}P_\mathrm{Lo}{}^2\overline{P_\mathrm{Sp}(z)}^2 = \frac{N_1}{24}\left(3k_\mathrm{Pol}{}^2-3+\frac{k_\mathrm{Pol}{}^2+3}{SNR_\mathrm{Sp}{}^2}+6N_1\right)\mathcal{R}_\mathrm{p}{}^4P_\mathrm{Lo}{}^2\overline{P_\mathrm{Sp}(z)}^2 \tag{S122}$$

2) The term of LPF$\{2I_s(z)I_e(z)\}$. According to Eq. (S86) and Eq. (S109), $2I_s(z)I_e(z)$ can be expressed as:

$$2I_s(z)I_e(z) = 4\mathcal{R}_\mathrm{p}\sum_{i=1}^{N_1}\Upsilon_i(z)I_e^I(z)\cos[H(z)] - 4\mathcal{R}_\mathrm{p}\sum_{i=1}^{N_1}\Upsilon_i(z)I_e^Q(z)\sin[H(z)] \tag{S123}$$

Considering that both summation and low-pass filtering are linear processes, the following equation holds:

$$\mathrm{LPF}\{I_s(z)I_e(z)\} = 4\mathcal{R}_\mathrm{p}\mathrm{LPF}\left\{\sum_{i=1}^{N_1}\Upsilon_i(z)I_e^I(z)\cos[H(z)]\right\} + 4\mathcal{R}_\mathrm{p}\mathrm{LPF}\left\{\sum_{i=1}^{N_1}\Upsilon_i(z)I_e^Q(z)\sin[H(z)]\right\} = 4\mathcal{R}_\mathrm{p}\sum_{i=1}^{N_1}\mathrm{LPF}\{\Upsilon_i(z)I_e^I(z)\cos[H(z)]\} + 4\mathcal{R}_\mathrm{p}\sum_{i=1}^{N_1}\mathrm{LPF}\{\Upsilon_i(z)I_e^Q(z)\sin[H(z)]\} \tag{S124}$$

The expectation value and variance of LPF$\{2I_s(z)I_e(z)\}$, designated as $\mu_{2se}(z)$ and $\sigma_{2se}(z)^2$, respectively, can be expressed as:

$$\mu_{2se}(z) = \overline{\mathrm{LPF}\{I_s(z)I_e(z)\}} = 4\mathcal{R}_\mathrm{p}\sum_{i=1}^{N_1}\overline{\mathrm{LPF}\{\Upsilon_i(z)I_e^I(z)\cos[H(z)]\}} + 4\mathcal{R}_\mathrm{p}\sum_{i=1}^{N_1}\overline{\mathrm{LPF}\{\Upsilon_i(z)I_e^Q(z)\sin[H(z)]\}} \tag{S125}$$

$$\sigma_{2se}(z)^2 = \mathrm{D}\{\mathrm{LPF}\{I_s(z)I_e(z)\}\} = 16\mathcal{R}_\mathrm{p}{}^2\left\{\sum_{i=1}^{N_1}\mathrm{D}\left\{\mathrm{LPF}\{\Upsilon_i(z)I_e^\mathrm{I}(z)\cos[H(z)]\}\right\} + \sum_{i=1}^{N_1}\mathrm{D}\left\{\mathrm{LPF}\{\Upsilon_i(z)I_e^\mathrm{Q}(z)\sin[H(z)]\}\right\}\right\} \tag{S126}$$

We then proceed to derive the statistics of $\mathrm{LPF}\{\Upsilon_i(z)I_e^\mathrm{I}(z)\cos[H(z)]\}$ and $\mathrm{LPF}\{\Upsilon_i(z)I_e^\mathrm{Q}(z)\sin[H(z)]\}$. We obtain:

$$\overline{\mathrm{LPF}\{\Upsilon_i(z)I_e^\mathrm{I}(z)\cos[H(z)]\}} = 0 \tag{S127}$$

and

$$\mathrm{D}\left\{\mathrm{LPF}\{\Upsilon_i(z)I_e^\mathrm{I}(z)\cos[H(z)]\}\right\} = \overline{\mathrm{LPF}^2\{\Upsilon_i(z)I_e^\mathrm{I}(z)\cos[H(z)]\}} - \overline{\mathrm{LPF}\{\Upsilon_i(z)I_e^\mathrm{I}(z)\cos[H(z)]\}}^2 = \frac{1}{16}\sigma_e{}^2B_\mathrm{BPF}\left[P_\mathrm{Lo1}\overline{P_\mathrm{Sp1}(z)} + P_\mathrm{Lo2}\overline{P_\mathrm{Sp2}(z)}\right] = \frac{1}{32}P_\mathrm{Lo}\overline{P_\mathrm{Sp}(z)}\sigma_e{}^2B_\mathrm{BPF} \tag{S128}$$

By analogy, it can be concluded that $\mathrm{LPF}\{\Upsilon_i(z) I_e^Q(z)\sin[H(z)]\}$ possesses the same mathematical expectation and variance as $\mathrm{LPF}\{\Upsilon_i(z) I_e^I(z)\cos[H(z)]\}$. Based on Eqs. (S125) and (S126), we have:

$$\mu_{2se}(z) = 0 \tag{S129}$$

$$\sigma_{2se}(z)^2 = N_1 \mathcal{R}_\mathrm{p}{}^2 P_\mathrm{Lo} \overline{P_\mathrm{Sp}(z)} \sigma_e{}^2 B_\mathrm{BPF} \tag{S130}$$

3) The term of LPF$\{\boldsymbol{I_e(z)^2}\}$. The statistical parameters of $\mathrm{LPF}\{I_e(z)^2\}$ – namely its expectation and variance – are the same as the case of single-pulse BOTDR in **Note S4** and **Note S5**.

The statistical parameters of the SMF-based coded-pulse BOTDR (expectation values and variances) derived above are summarized in Table S4. Up to this point, we have derived the SNR of the coded response:

$$\mathrm{SNR}\{r_\mathrm{Code}^\mathrm{SMF}(z)\} = \frac{\frac{1}{2} N_1 \mathcal{R}_\mathrm{p}{}^2 P_\mathrm{Lo} \overline{P_\mathrm{Sp}(z)}}{\sqrt{\frac{N_1}{24}\left(3k_\mathrm{Pol}{}^2 - 3 + \frac{k_\mathrm{Pol}{}^2 + 3}{SNR_\mathrm{Sp}{}^2} + 6N_1\right)\mathcal{R}_\mathrm{p}{}^4 P_\mathrm{Lo}{}^2 \overline{P_\mathrm{Sp}(z)}^2 + N_1 \mathcal{R}_\mathrm{p}{}^2 P_\mathrm{Lo} \overline{P_\mathrm{Sp}(z)} \sigma_e{}^2 B_\mathrm{BPF} + \sigma_e{}^4 B_\mathrm{BPF}{}^2}} \tag{S131}$$

The SNR of the decoded response can then be obtained via compressing the noise STD by a factor of $\sqrt{N_1/2}$ [18,19]:

$$\begin{aligned}\mathrm{SNR}\{r_\mathrm{Decode}^\mathrm{SMF}(z)\} &= \frac{\frac{1}{2} \mathcal{R}_\mathrm{p}{}^2 P_\mathrm{Lo} \overline{P_\mathrm{Sp}(z)}}{\sqrt{\frac{2}{N_1}}\sqrt{\frac{N_1}{24}\left(3k_\mathrm{Pol}{}^2 - 3 + \frac{k_\mathrm{Pol}{}^2 + 3}{SNR_\mathrm{Sp}{}^2} + 6N_1\right)\mathcal{R}_\mathrm{p}{}^4 P_\mathrm{Lo}{}^2 \overline{P_\mathrm{Sp}(z)}^2 + N_1 \mathcal{R}_\mathrm{p}{}^2 P_\mathrm{Lo} \overline{P_\mathrm{Sp}(z)} \sigma_e{}^2 B_\mathrm{BPF} + \sigma_e{}^4 B_\mathrm{BPF}{}^2}} \\ &\le \sqrt{\frac{3}{\left(3 + \frac{1}{SNR_\mathrm{Sp}{}^2}\right) k_\mathrm{Pol}{}^2 - 3 + \frac{3}{SNR_\mathrm{Sp}{}^2} + 6N_1}}\end{aligned} \tag{S132}$$

Eq. (S132) reveals that, under a given k_Pol value, the SNR upper limit of the decoded BOTDR is predicted to decrease as N_1 increases, and is always lower than the SNR upper limit of the single-pulse sensing system shown by Eq. (S106). This is because the decoded SNR performance of coded-pulse BOTDR is severely constrained by the intrinsic fluctuation of SpBS and polarization noise.

The coding gain, typically defined as the SNR improvement provided by the coding scheme in comparison to the single-pulse scheme, can be expressed as:

$$
\begin{aligned}
G_c &= \frac{\mathrm{SNR}\{r_{\mathrm{Decode}}^{\mathrm{SMF}}(z)\}}{\mathrm{SNR}\{r_{\mathrm{Sg}}^{\mathrm{SMF}}(z)\}} \\
&= \sqrt{\frac{N_1}{2}}\sqrt{\frac{\frac{(3SNR_{\mathrm{Sp}}{}^2+1)k_{\mathrm{Pol}}{}^2+3SNR_{\mathrm{Sp}}{}^2+3}{24SNR_{\mathrm{Sp}}{}^2}\mathcal{R}_{\mathrm{p}}{}^4P_{\mathrm{Lo}}{}^2\overline{P_{\mathrm{Sp}}(z)}^2+\mathcal{R}_{\mathrm{p}}{}^2P_{\mathrm{Lo}}\overline{P_{\mathrm{Sp}}(z)}\sigma_e{}^2B_{\mathrm{BPF}}+\sigma_e{}^4B_{\mathrm{BPF}}{}^2}{\frac{N_1}{24}\left(3k_{\mathrm{Pol}}{}^2-3+\frac{k_{\mathrm{Pol}}{}^2+3}{SNR_{\mathrm{Sp}}{}^2}+6N_1\right)\mathcal{R}_{\mathrm{p}}{}^4P_{\mathrm{Lo}}{}^2\overline{P_{\mathrm{Sp}}(z)}^2+N_1\mathcal{R}_{\mathrm{p}}{}^2P_{\mathrm{Lo}}\overline{P_{\mathrm{Sp}}(z)}\sigma_e{}^2B_{\mathrm{BPF}}+\sigma_e{}^4B_{\mathrm{BPF}}{}^2}} \\
&= \begin{cases} \sqrt{\dfrac{(3SNR_{\mathrm{Sp}}{}^2+1)k_{\mathrm{Pol}}{}^2+3SNR_{\mathrm{Sp}}{}^2+3}{2(3SNR_{\mathrm{Sp}}{}^2+1)k_{\mathrm{Pol}}{}^2+12N_1SNR_{\mathrm{Sp}}{}^2-6SNR_{\mathrm{Sp}}{}^2+6}}, & \text{for high SpBS signal level} \\ \sqrt{\dfrac{N_1}{2}}, & \text{for low SpBS signal level} \end{cases}
\end{aligned}
\tag{S133}
$$

It can be concluded that the coding gain exhibits distinct behavior across two characteristic sensing regions:

1) Region I – High SpBS signal power: This region corresponds to scenarios where the spontaneous Brillouin scattering (SpBS) signal power is sufficiently high such that the combined effect of SpBS noise and polarization noise, represented by the first noise term in the numerator of Eq. (S131), dominates. Under this condition, no positive coding gain can be realized due to the joint constraints imposed by both noise sources. In fact, SNR degrades with increasing code length N_1, given fixed values of k_{Pol} and SNR_{Sp}. This situation typically arises near the input end of long fibers or along the full length of shorter fibers.
2) Region II – Low SpBS signal power: In this region, the SpBS signal power is sufficiently low that photodetection noise, represented by the third term in the numerator of Eq. (S131), becomes the dominant factor. Since SpBS and polarization noise scale with signal magnitude, they become negligible here, allowing for a positive coding gain. In the ideal case, the gain approaches $\sqrt{N_1/2}$. This condition usually occurs near the far end of long fibers, where signal attenuation significantly reduces SpBS power, or when the pump pulse duration or power is set low.

2. Experimental verification

To experimentally assess the influence of SpBS noise on coded-pulse BOTDR, we employed a genetic-algorithm-optimized coding scheme[19] within a standard coherent detection setup over a 50 km-long standard SMF. Each codeword comprises pulses with a 20 ns base duration, digitally upsampled to 200 ns before injection into the fiber. The leading pulse was amplified to the modulation instability (MI) threshold using an EDFA[20]. We used a 5 dBm dual-sideband OLO and tested three distinct N_1 values: 8, 20, and 45. At the receiver, we implemented a physical envelope detection scheme: The hybrid SpBS-OLO signal was captured by a 400-MHz bandwidth BPD, then sequentially processed through: 1) an electrical amplifier, 2) a 50-MHz bandpass filter, and 3) a physical envelope detector, before digitization at 500 MSa s^{-1} using an ADC. The beat frequency was carefully tuned to match the center frequency of the bandpass filter. Each trace was obtained by averaging 2,048 acquisitions within a 530 μs sampling window. Twenty such traces were collected per code length to ensure robust SNR estimation. A single-pulse measurement under identical conditions served as the baseline.

Figure S18a-c shows SNR measurements for N_1 = 8, 20, and 45, which closely align with theoretical predictions. The results confirm that SNR improvements from coding are significantly less than conventionally expected due to the limiting effects of SpBS noise, especially at higher N_1. However, as the sensing distance increases and attenuation suppresses SpBS and polarization noise, the SNR gradually approaches the photodetection-limited regime. These findings highlight the critical role of SpBS noise in coded-pulse BOTDR and underscore the need for more careful optimization strategies that account for its fundamental limitations.

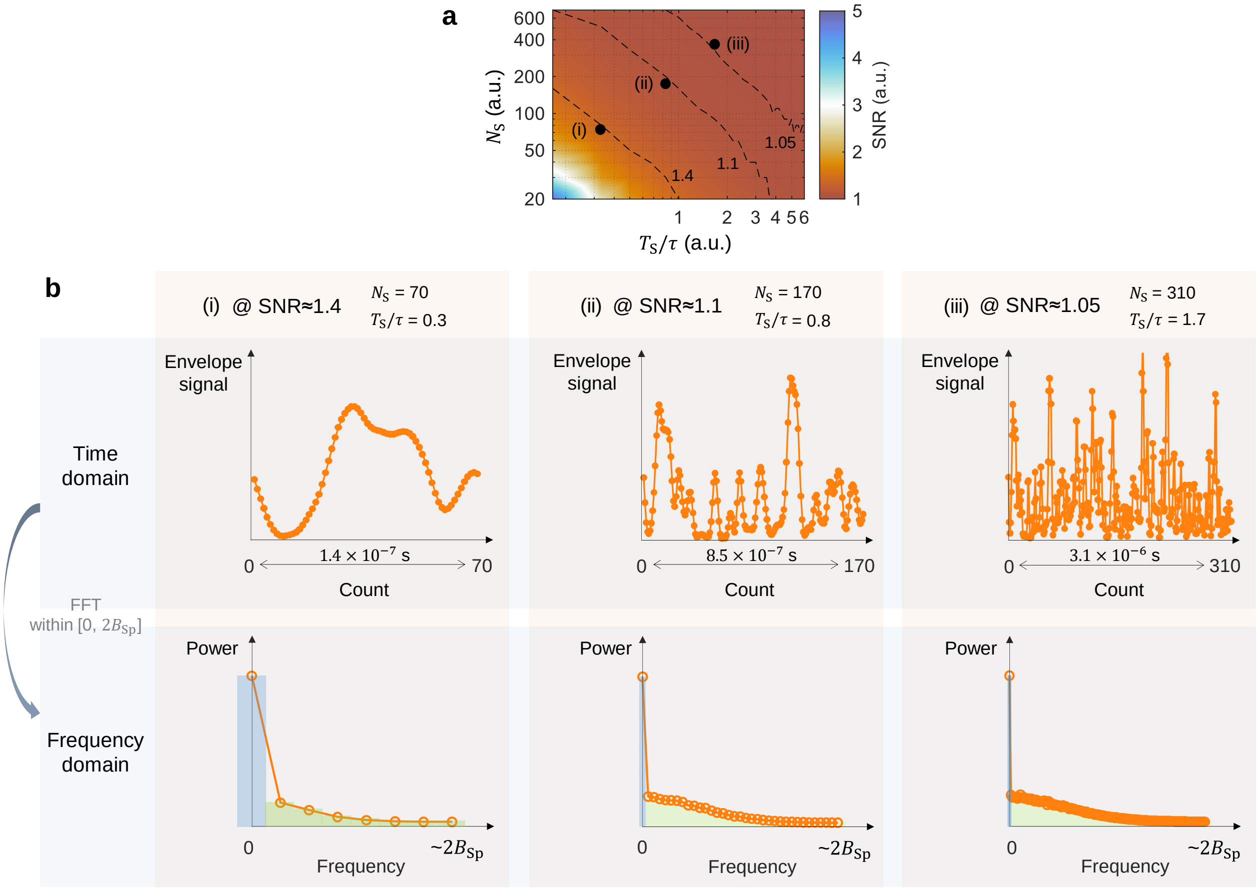

Fig. S1. Time–frequency characterization across different sampling conditions. $\boldsymbol{T_S}$: Sampling interval. $\boldsymbol{N_S}$: Total number of samples. $\boldsymbol{\tau}$: acoustic lifetime. **a** **Fig. 2f** of the original manuscript. Theoretical 2D SNR map as a function of $\boldsymbol{T_S}$ and $\boldsymbol{N_S}$, with $\boldsymbol{\tau}$ = 6 ns. Dashed contour lines (black dashed lines) on the map indicate SNR levels of 1.4, 1.1, and 1.05. The three black dots illustrate the selected sampling conditions corresponding to three different SNR levels. **b** Inset (i)-(iii) showcase the envelope signal in time domain and corresponding power envelope spectra in frequency domain under the sampling conditions indicated by the three black dots in **a**. The frequency-domain plots in the second row are excerpted from **Fig. 2g** of the original text, highlighting the DC (blue shading) and AC (green shading) contributions within spectral range [0, $\boldsymbol{2B_{Sp}}$]. In the case of oversampling and small acquisition number of points (e.g., $\boldsymbol{N_S}$ = 70, $\boldsymbol{T_S/\tau}$ = 0.3), the temporal samples are highly correlated, and the short acquisition window reduces spectral resolution. This limits the number of AC components and yields an artificially inflated SNR, defined by the DC-to-AC ratio. In contrast, increasing the number of points and the sampling interval extends the temporal window, provides more decorrelated samples, improves spectral resolution, and leads to a more realistic SNR estimation.

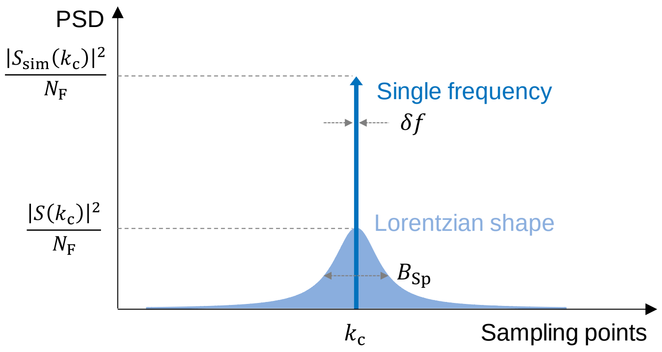

Fig. S2. Schematic diagram of single-frequency and Lorentzian spectra.

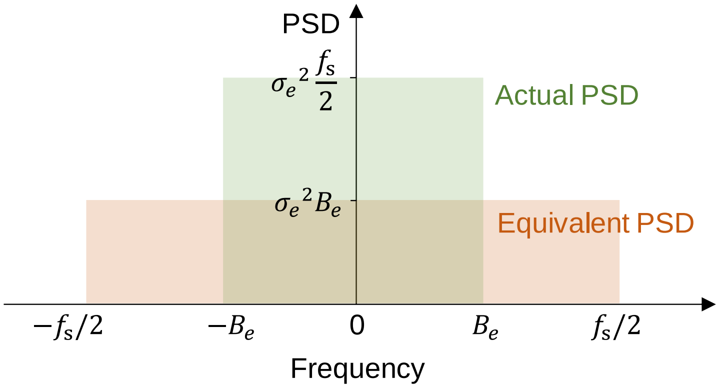

Fig. S3. Schematic diagram illustrating the equivalent and actual noise power spectral density (PSD).

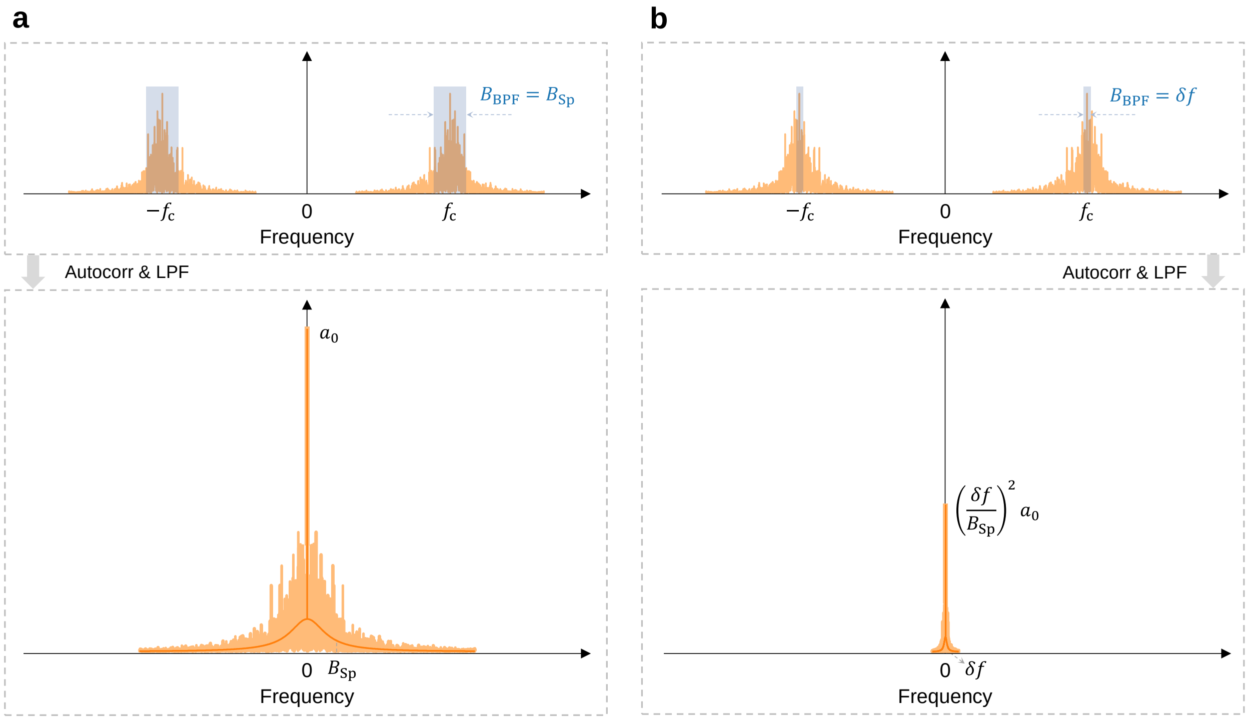

Fig. S4. Spectral interpretation on SNR behavior of FFT-based coherent detection. a Spectrum of $\overrightarrow{\boldsymbol{E}_{\mathbf{Sp}}}(\boldsymbol{t})$ with a BPF of bandwidth $\boldsymbol{B}_{\mathbf{BPF}} = \boldsymbol{B}_{\mathbf{Sp}}$, and its corresponding power envelope spectrum. **b** Spectrum of $\overrightarrow{\boldsymbol{E}_{\mathbf{Sp}}}(\boldsymbol{t})$ with a BPF of bandwidth $\boldsymbol{B}_{\mathbf{BPF}} = \boldsymbol{\delta f}$, and its corresponding power envelope spectrum.

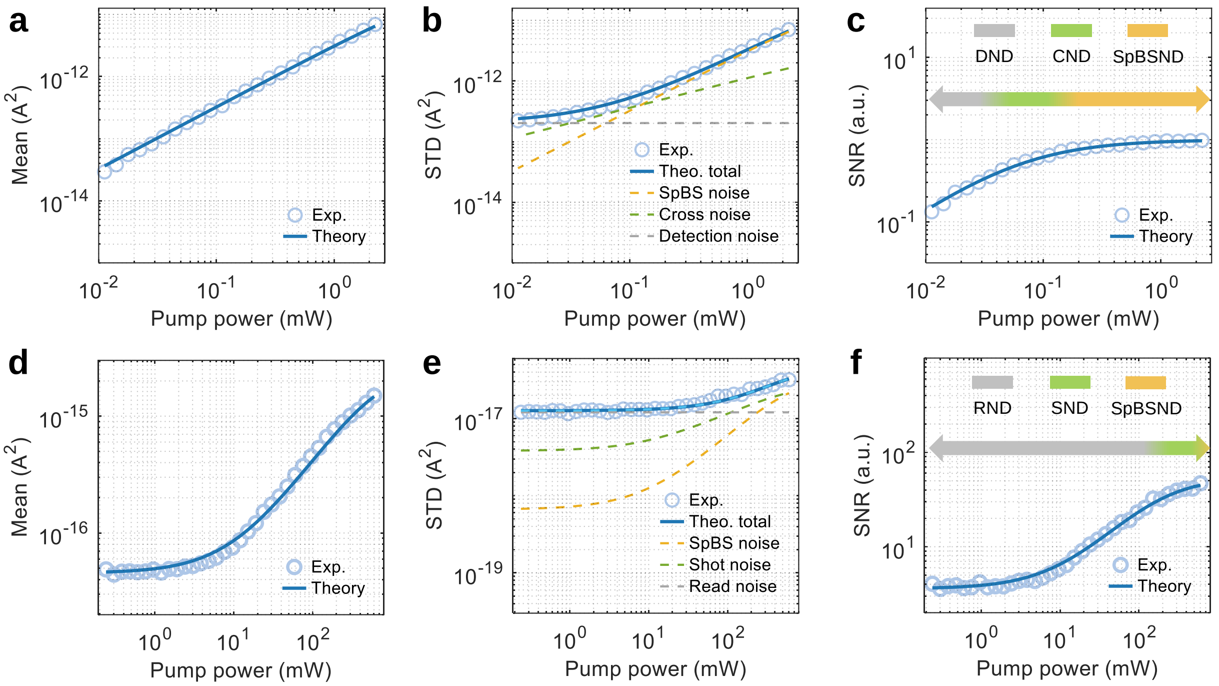

Fig. S5. Results for the anti-Stokes signal. Comparison between the theoretically calculated and experimentally measured mean signal, noise standard deviation and SNR for (**a-c**) the coherent detection scheme and (**d-f**) the direct detection scheme with measurement bandwidth B_{m}= 50 kHz. RND: read noise domination; SND: shot noise domination; SpBSND: SpBS noise domination. The experimental data used here are obtained from the same experiments as in **Fig. 2** of the main text and analyzed using the same theoretical methods, with the difference that both measurements and analyses here are performed for the anti-Stokes signal.

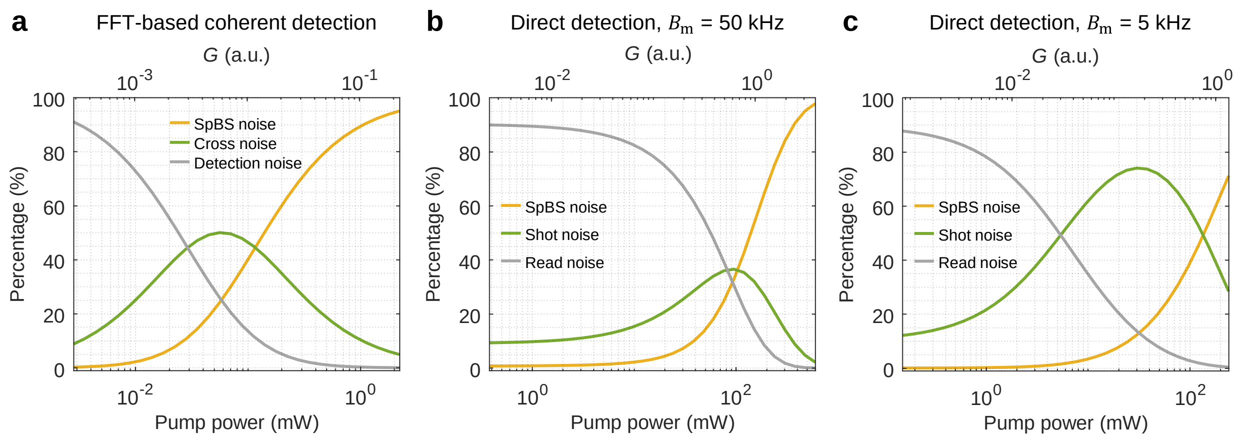

Fig. S6. Relative contributions of individual noise terms as functions of pump power and system gain *G*. Using the same data (with pump present) as in **Figs. 3e**, **4b**(ii) and **4c**(ii) of the main text, these plots show the theoretical relative contributions of different noise variances to the total noise variance under varying pump powers and system gains *G*. The color scheme is consistent with **Figs. 3e**, **4b**(ii) and **4c**(ii) of the main text. **a** SpBS noise, cross noise, and detection noise in the FFT-based coherent detection experiment. **b** SpBS noise, shot noise, and read noise in direct detection with system bandwidth $\boldsymbol{B}_{\mathbf{m}}$= 50 kHz. **c** Same as **b**, but with $\boldsymbol{B}_{\mathbf{m}}$= 5 kHz.

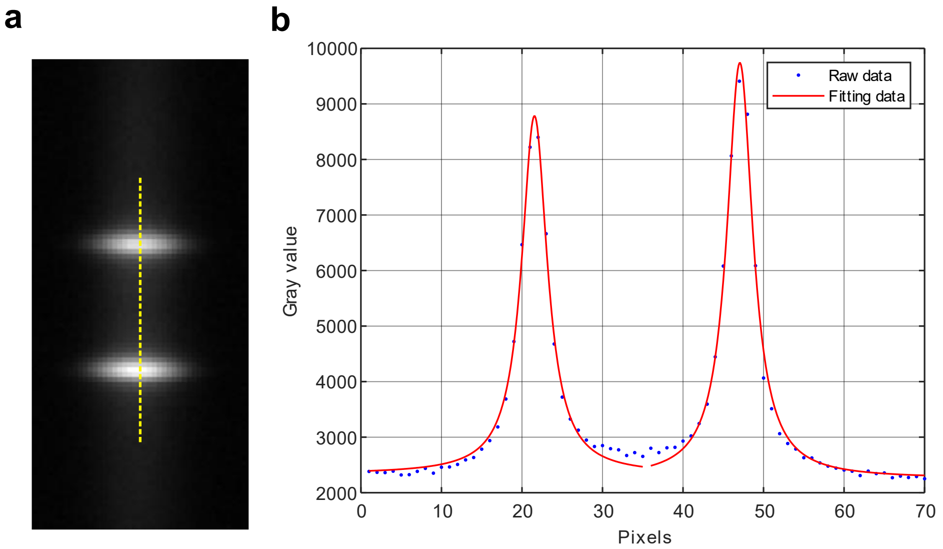

Fig. S7. Raw data and Lorentzian fit of the Brillouin signal from a 10 m fiber acquired via direct detection. a sCMOS image of the Brillouin spectrum from a 10 m-long polarization-maintaining fiber. Two distinct peaks are visible, corresponding (from top to bottom) to the anti-Stokes and Stokes Brillouin signals. Note that due to the flipped image orientation in our detection setup, the shorter-wavelength Stokes signal appears at the bottom of the image. **b** Intensity profile (gray value plot) extracted along with the longitudinal yellow dashed line in panel a, along with corresponding Lorentzian fits to the anti-Stokes and Stokes peaks.

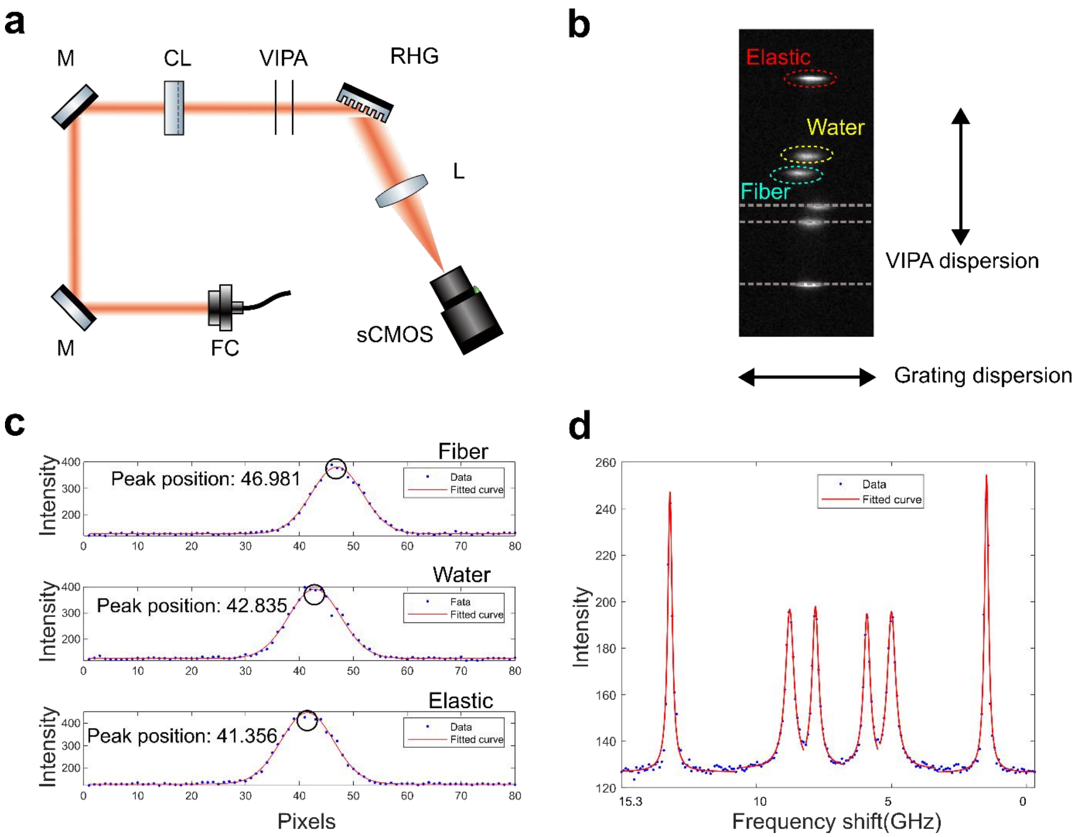

Fig. S8. Quantitative measurement of fiber frequency shift using VIPA with grating spectrometer. a Schematic of the VIPA spectrometer setup incorporating a 2D dispersive grating. FC: fiber collimator; M: mirror; CL: cylindrical lens; VIPA: Virtually Imaged Phased Array; RHG: reflective holographic grating; L: lens; sCMOS: scientific Complementary Metal–Oxide–Semiconductor camera. **b** Image acquired from sCMOS showing elastic scattering and Brillouin signals from water and fiber, measured using the VIPA with grating spectrometer. **c** Horizontal gray-value profiles extracted from the image in b along three gray dashed lines, corresponding (from top to bottom) to the anti-Stokes Brillouin signal of the fiber, the anti-Stokes signal of water, and the elastic scattering peak. Based on the known Brillouin frequency shift of water (5.07 GHz at 780 nm pump laser) and the linear dispersion of the grating, the relative horizontal displacement of the fiber and water signals with respect to the elastic peak (Δfiber / Δwater ≈ 3.8) suggests that the fiber's Brillouin shift lies between approximately 19.3 GHz and 35 GHz. **d** Simultaneous plots of the three signal groups (raw and fitted data) along the VIPA dispersion axis. Using the known dispersion characteristics of the VIPA, the 15.3 GHz free spectral range (FSR) of the VIPA, and the 5.07 GHz Brillouin shift of water under 780 nm laser excitation, the Brillouin frequency shift of the fiber is accurately determined to be 21.7 GHz.

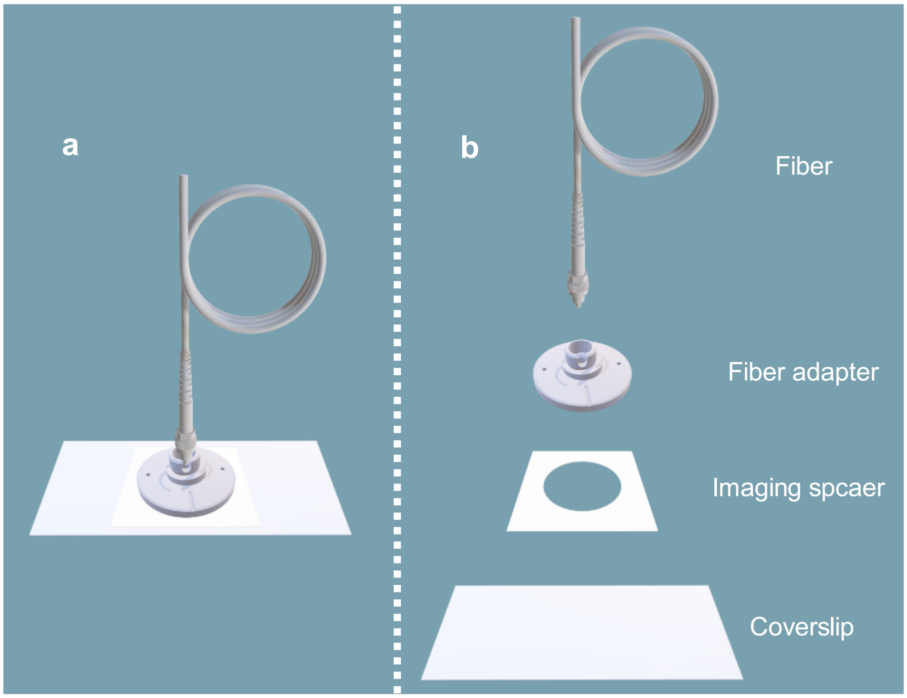

Fig. S9. Schematic of the fiber sample used for Brillouin imaging. a Overview schematic illustrating the full composition and structure of the fiber sample. **b** Enlarged view highlighting the internal subdivisions and specific regions of interest within the fiber sample.

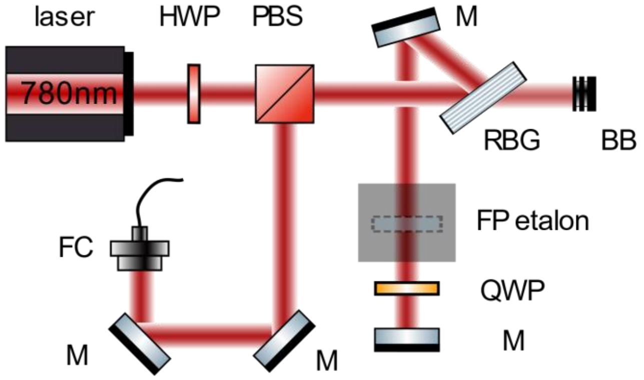

Fig. S10. Setup of the ASE-filtered module with temperature-controlled FP etalon. The setup comprises a double-pass Reflective Bragg grating (RBG) and a temperature-controlled Fabry–Pérot (FP) etalon to form a narrowband filter (<250 MHz), effectively suppressing amplified spontaneous emission (ASE) noise from the 780 nm pump laser. HWP: half-wave plate; QWP: quarter-wave plate; PBS: polarizing beam splitter; M: mirror; RBG: Reflective Bragg grating; BB: beam block; FC: fiber coupler.

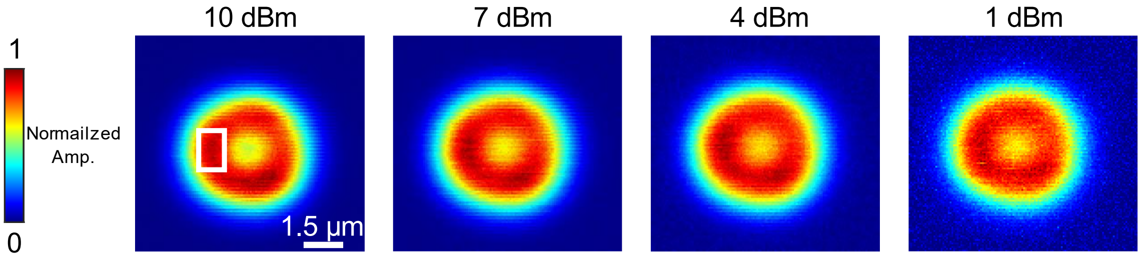

Fig. S11. Brillouin signal amplitude of a fiber sample using the VIPA spectrometer. Brillouin signal amplitude images of a 1 km, 1550 nm fiber sample acquired under varying pump powers in dBm. White square in the images marks the region with the strongest signal from the fiber sample.

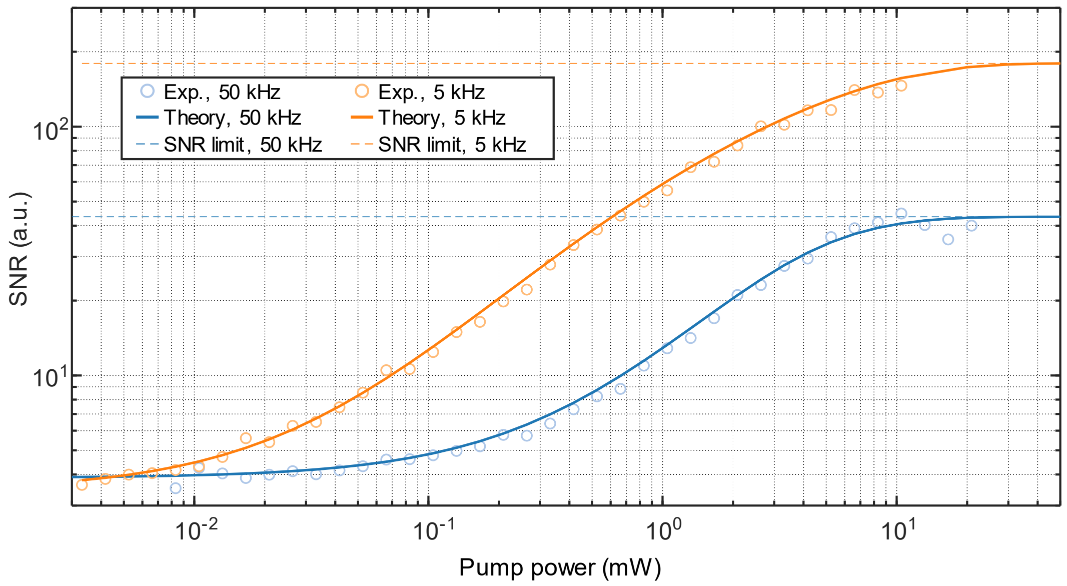

Fig. S12. Brillouin signal SNR analysis of a fiber sample using the VIPA spectrometer. Theoretical and experimental SNR analysis of the fiber's Brillouin signal under different detection bandwidths. The sCMOS camera acquisition mode was set to low gain for all measurements.

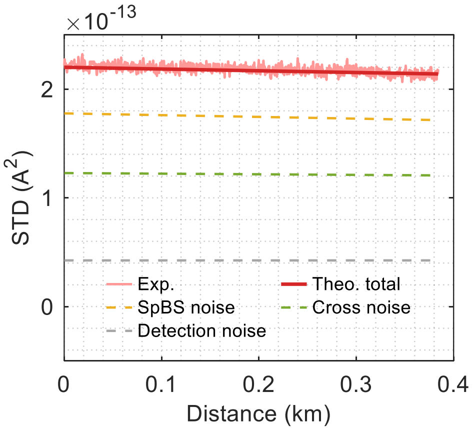

Fig. S13. Noise standard deviations along the sensing distance. Standard deviations of SpBS noise, cross noise, detection noise, and total noise versus sensing distance for a 400 m PMF, with a 10 ns pulse duration and a 26 dBm pump power. The data used here are the same as those presented in **Fig. 6c** of the main text.

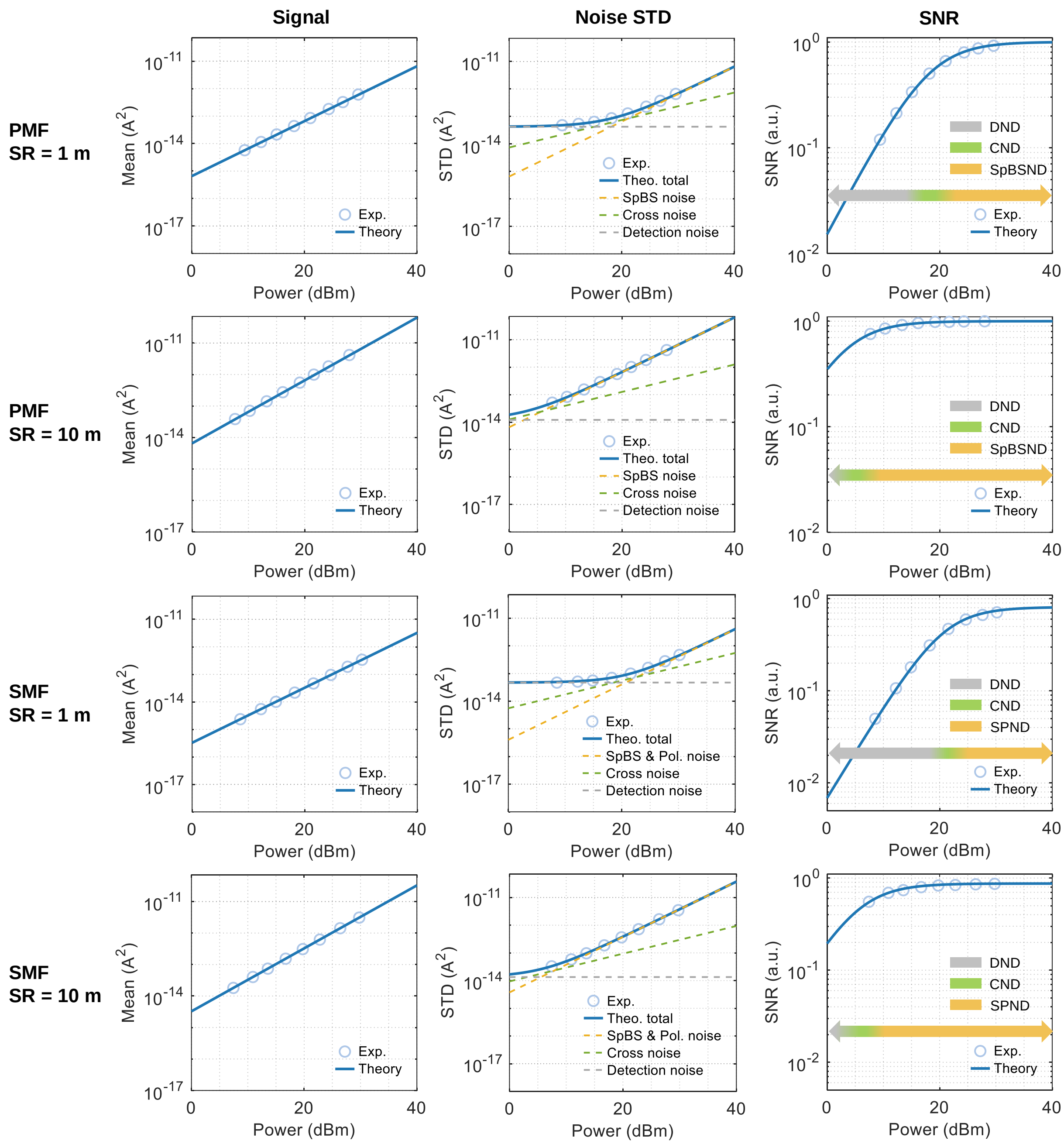

Fig. S14. Supplementary results for single-pulse BOTDR. Comparison between theoretical predictions and experimental measurements of mean signal, noise standard deviation, and SNR for different fiber types and pulse durations: 400 m PMF with 10 ns and 100 ns pulses, and 1.9 km SMF with 10 ns and 100 ns pulses. DND: detection noise domination; CND: cross-term noise domination; SPND: SpBS and polarization noise domination. The data here are identical to those in **Fig. 6** of the main text, and, as in **Fig. 6**, are plot as a function of the pump power. The vertical axis scale of each metric is kept consistent. Following the presentation style of the main text, the theoretical contributions of individual noise terms are plotted in the noise STD subplot, while in the SNR subplot, the contributions of each noise term are distinguished using colored bars.

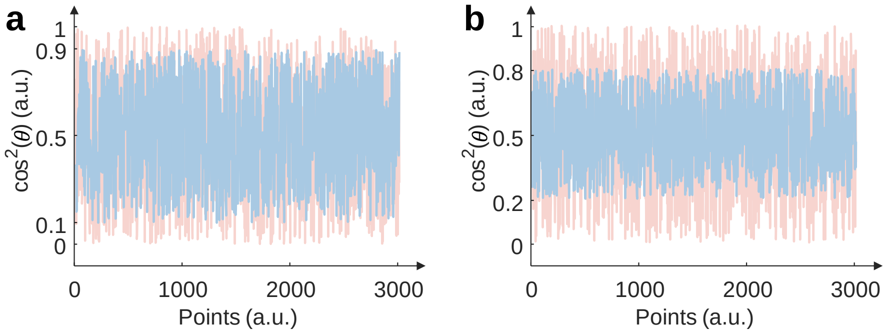

Fig. S15. Simulated fluctuation characteristics of $\cos^2(\theta)$ affected by the self-polarization-scrambling effect. The blue curves illustrate the cases of **a** k_{Pol} = 0.8 and **b** k_{Pol} = 0.6, while the pink curves represent the reference case where k_{Pol} = 1.

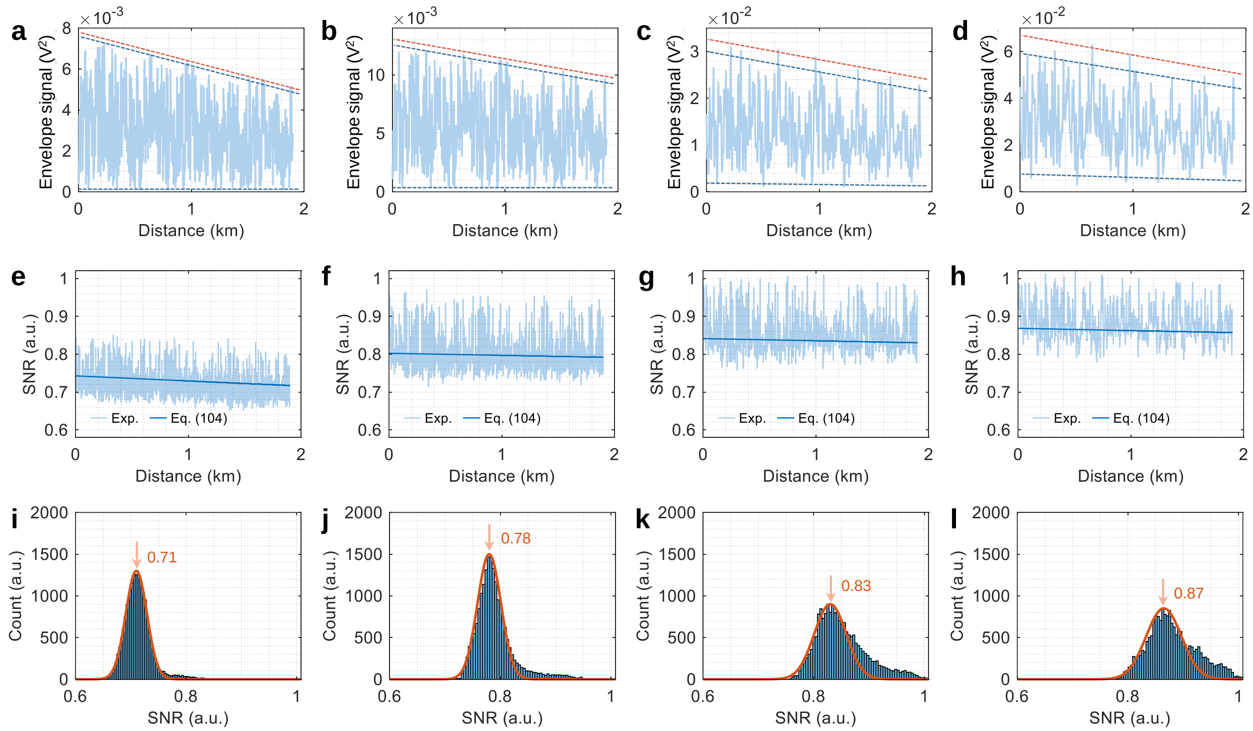

Fig. S16. Experimental results of the analysis on the self-polarization-scrambling effect. a-b Measured BOTDR envelope signals (1000 trace averaging) along 1.9 km SMF without PSc, illustrating the degree of self-polarization-scrambling effect under SRs of 1 m, 2 m, 6 m and 10 m, respectively. The two blue dashed lines indicate the actual upper and lower limits of the envelope signal fluctuation, while the red dashed line indicates the maximum value of the envelope signal in the absence of self-polarization-scrambling effect. **e-h** Light bule curves show the measured SNR profiles along 1.9 km standard SMF with PSc, under SRs of 1 m, 2 m, 6 m and 10 m, respectively. Dark blue curves show the corresponding SNR values predicted by Eq. (S106). **i-l** Distribution histograms of the SNR under SRs of 1 m, 2 m, 6 m and 10 m, respectively. The red line represents the fitting result for the primary Gaussian-like histogram.

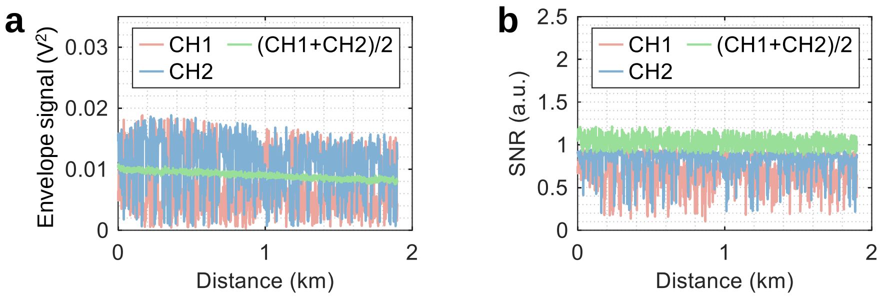

Fig. S17. Experimental results of BOTDR based on polarization diversity coherence receiver (PDCR). a Envelope signals traces and **b** SNR traces of PDCR before and after the combination of the two channels for the 2 m SR case.

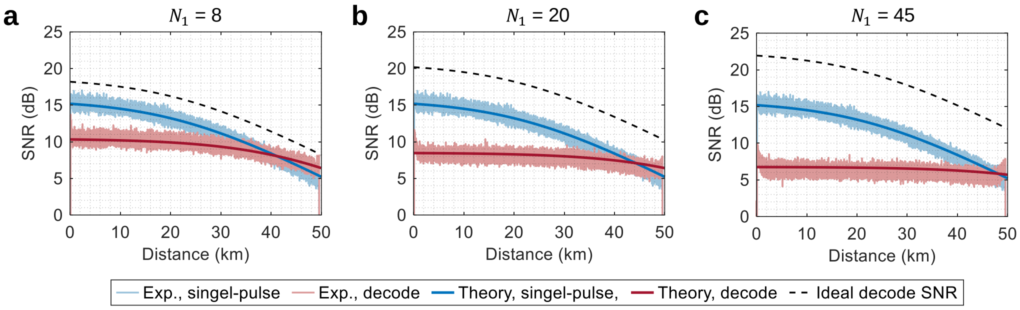

Fig. S18. SNR behaviour of coded-pulse BOTDR along a 50 km standard SMF. a $\boldsymbol{N_1}$ = 8, **b** $\boldsymbol{N_1}$ = 20, and **c** $\boldsymbol{N_1}$ = 45. The three subplots share a single-pulse SNR curve as a reference. The duration of each coded pulse is 20 ns, matching that of the single pulse. For different coding bit numbers, the peak power of the first coded pulse is set equal to that of the single pulse, which is optimized to the modulation instability threshold.

Table S1. Expectation values and variances of the three terms in Eq. (S46).

Term	Expectation	Variance
$\frac{\lvert \boldsymbol{S}(\boldsymbol{k}_{\mathbf{c}})\rvert^2}{\boldsymbol{N}_{\mathbf{F}}}$	$\frac{2f_\mathrm{s}}{\pi B_\mathrm{Sp}}\mathcal{R}_\mathrm{p}{}^2 P_\mathrm{Lo}\overline{P_\mathrm{Sp}}$<br>Eq. (S54)	$\frac{4{f_\mathrm{s}}^2}{\pi^2 {B_\mathrm{Sp}}^2}\mathcal{R}_\mathrm{p}{}^4 {P_\mathrm{Lo}}^2 \frac{\overline{P_\mathrm{Sp}}^2}{{SNR_\mathrm{Sp}}^2}$<br>Eq. (S55)
$\frac{\lvert \boldsymbol{E}(\boldsymbol{k}_{\mathbf{c}})\rvert^2}{\boldsymbol{N}_{\mathbf{F}}}$	${\sigma_e}^2 \frac{f_\mathrm{s}}{2}$<br>Eq. (S70)	$\left({\sigma_e}^2 \frac{f_\mathrm{s}}{2}\right)^2$<br>Eq. (S71)
**Cross term**	0<br>Eq. (S72)	$\frac{2{f_\mathrm{s}}^2}{\pi B_\mathrm{Sp}}\mathcal{R}_\mathrm{p}{}^2 P_\mathrm{Lo}\overline{P_\mathrm{Sp}}{\sigma_e}^2$<br>Eq. (S74)

Table S2. Expectation values and variances of the three terms in Eq. (S78).

Term	Expectation	Variance
$\mathbf{LPF}\{\boldsymbol{I_s}(\boldsymbol{z})^{\mathbf{2}}\}$	$\mathcal{R}_{\mathrm{p}}{}^2 P_{\mathrm{Lo}} \overline{P_{\mathrm{Sp}}(z)}$ Eq. (S81)	$\frac{1}{2}\left(1+\frac{1}{SNR_{\mathrm{Sp}}{}^2}\right)\mathcal{R}_{\mathrm{p}}{}^4 P_{\mathrm{Lo}}{}^2 \overline{P_{\mathrm{Sp}}(z)}^2$ Eq. (S85)
$\mathbf{LPF}\{\mathbf{2}\boldsymbol{I_s}(\boldsymbol{z})\boldsymbol{I_e}(\boldsymbol{z})\}$	0 Eq. (S88)	$2\mathcal{R}_{\mathrm{p}}{}^2 P_{\mathrm{Lo}} \overline{P_{\mathrm{Sp}}(z)} \sigma_e{}^2 B_{\mathrm{BPF}}$ Eq. (S89)
$\mathbf{LPF}\{\boldsymbol{I_e}(\boldsymbol{z})^{\mathbf{2}}\}$	$\sigma_e{}^2 B_{\mathrm{BPF}}$ Eq. (S93)	$(\sigma_e{}^2 B_{\mathrm{BPF}})^2$ Eq. (S94)

Table S3. Expectation values and variances of the three terms in an expression similar to Eq. (S78) for the SMF case.

Term	Expectation	Variance
$\mathbf{LPF}\{\boldsymbol{I_s(z)^2}\}$	$\frac{1}{2}\mathcal{R}_{\mathrm{p}}{}^2 P_{\mathrm{Lo}}\overline{P_{\mathrm{Sp}}(z)}$ Eq. (S100)	$\frac{3SNR_{\mathrm{Sp}}{}^2\left(k_{\mathrm{Pol}}{}^2+1\right)+k_{\mathrm{Pol}}{}^2+3}{24SNR_{\mathrm{Sp}}{}^2}\mathcal{R}_{\mathrm{p}}{}^4 P_{\mathrm{Lo}}{}^2\overline{P_{\mathrm{Sp}}(z)}^2$ Eq. (S103)
$\mathbf{LPF}\{\boldsymbol{2I_s(z)I_e(z)}\}$	0 Eq. (S104)	$\mathcal{R}_{\mathrm{p}}{}^2 P_{\mathrm{Lo}}\overline{P_{\mathrm{Sp}}(z)}\sigma_e{}^2 B_{\mathrm{BPF}}$ Eq. (S105)
$\mathbf{LPF}\{\boldsymbol{I_e(z)^2}\}$	$\sigma_e{}^2 B_{\mathrm{BPF}}$ Eq. (S93)	$\sigma_e{}^4 B_{\mathrm{BPF}}{}^2$ Eq. (S94)

Table S4. Expectation values and variances of the three terms in an expression similar to Eq. (S78) for the coded-pulse case.

Terms	Expectation	Variance
$\mathbf{LPF}\{\boldsymbol{I_s}(\boldsymbol{z})^{\mathbf{2}}\}$	$\frac{1}{2}N_1\mathcal{R}_{\mathrm{p}}{}^2P_{\mathrm{Lo}}\overline{P_{\mathrm{Sp}}(z)}$ Eq. (S121)	$\frac{N_1}{24}\left(3k_{\mathrm{Pol}}{}^2-3+\frac{k_{\mathrm{Pol}}{}^2+3}{SNR_{\mathrm{Sp}}{}^2}+6N_1\right)\mathcal{R}_{\mathrm{p}}{}^4P_{\mathrm{Lo}}{}^2\overline{P_{\mathrm{Sp}}(z)}^2$ Eq. (S122)
$\mathbf{LPF}\{\mathbf{2}\boldsymbol{I_s}(\boldsymbol{z})\boldsymbol{I_e}(\boldsymbol{z})\}$	0 Eq. (S129)	$N_1\mathcal{R}_{\mathrm{p}}{}^2P_{\mathrm{Lo}}\overline{P_{\mathrm{Sp}}(z)}\sigma_e{}^2B_{\mathrm{BPF}}$ Eq. (S130)
$\mathbf{LPF}\{\boldsymbol{I_e}(\boldsymbol{z})^{\mathbf{2}}\}$	$\sigma_e{}^2B_{\mathrm{BPF}}$ Eq. (S93)	$(\sigma_e{}^2B_{\mathrm{BPF}})^2$ Eq. (S94)

REFERENCES

1. Boyd, R. W., Rząewski, K. & Narum, P. Noise initiation of stimulated Brillouin scattering. *Phys. Rev. A* **42**, 5514–5521 (1990).
2. Boyd, R. W. *Nonlinear Optics*, 3rd edn (Academic Press, 2008).
3. Beugnot, J.-C., Tur, M., Mafang, S. F. & Thévenaz, L. Distributed Brillouin sensing with sub-meter spatial resolution: modeling and processing. *Opt. Express* **19**, 7381–7397 (2011).
4. Loudon, R. *The Quantum Theory of Light*, 3rd ed. (Oxford Univ. Press, Oxford, New York, 2000).
5. Goodman, J. W. *Statistical Optics*, 2nd ed. (Wiley, Hoboken, NJ, 2015).
6. Proakis, J. G. & Manolakis, D. G. *Digital Signal Processing: Principles, Algorithms, and Applications*, 3rd edn (Prentice Hall, 1996).
7. Wang, S., Yang, Z., Soto, M. A. & Thévenaz, L. Study on the signal-to-noise ratio of Brillouin optical-time domain analyzers. *Opt. Express* **28**, 19864–19876 (2020).
8. Saleh, B. E. A. & Teich, M. C. *Fundamentals of Photonics*, 3rd ed. (Wiley, Hoboken, NJ, 2019).
9. Thévenaz, L. *Advanced Fiber Optics: Concepts and Technology* (EPFL Press, Lausanne, Boca Raton, 2011).
10. Jin, S., Yang, Z., Hong, X. & Wu, J. Analytical signal-to-noise ratio model on frequency-scanned Brillouin optical time-domain reflectometry. *J. Lightwave Technol.* **42**, 5786–5796 (2024).
11. Yariv, A. & Yeh, P. *Photonics: Optical Electronics in Modern Communications*, 6th ed. (Oxford Univ. Press, New York, 2007).
12. Johnson, R. A., Miller, I. & Freund, J. E. *Miller & Freund's Probability and Statistics for Engineers*, 9th ed. (Pearson, Boston, 2017).
13. van Deventer, M. O. & Boot, A. J. Polarization properties of stimulated Brillouin scattering in single-mode fibers. *J. Lightwave Technol.* **12**, 585–590 (1994).
14. Gao, X. *et al.* Impact of optical noises on unipolar-coded Brillouin optical time-domain analyzers. *Opt. Express* **29**, 22146–22158 (2021).
15. Jostmeier, T., Marx, B., Buntebarth, C., Rath, A. & Hill, W. Long-distance BOTDR interrogator with polarization-diverse coherent detection and power evaluation. In *Optical Fiber Sensors Conference 2020 Special Edition, paper T3.21.* https://doi.org/10.1364/OFS.2020.T3.21 (2020).
16. Song, H. Y. & Golomb, S. W. Some new constructions for simplex codes. IEEE Trans. Inform. Theory 40, 504–507 (1994).
17. Golay, M. Complementary series. *IEEE Trans. Inform. Theory* **7**, 82–87 (1961).
18. Yang, Z., Li, Z., Zaslawski, S., Thévenaz, L. & Soto, M. A. Design rules for optimizing unipolar coded Brillouin optical time-domain analyzers. *Opt. Express* **26**, 16505–16523 (2018).
19. Sun, X. *et al.* Genetic-optimised aperiodic code for distributed optical fibre sensors. *Nat. Commun.* **11**, 5774 (2020).
20. Alem, M., Soto, M. A. & Thévenaz, L. Analytical model and experimental verification of the critical power for modulation instability in optical fibers. *Opt. Express* **23**, 29514–29532 (2015).
